# A Time-Gated Raman Spectroscopy and Mass Spectrometry Comparative Chemical Analysis of Plasticizers

**DOI:** 10.1021/acspolymersau.6c00065

**Published:** 2026-07-13

**Authors:** Julie M. Rieland, Yamil Simón-Manso, Adam Zuber, Anthony P. Kotula, Kalman B. Migler, William E. Wallace, Kathryn L. Beers

**Affiliations:** † Materials Science and Engineering Division, 10833 National Institute of Standards and Technology, Gaithersburg, Maryland 20899, United States of America; ‡ Mass Spectrometry Data Center, Biomolecular Measurement Division, National Institute of Standards and Technology (NIST), Gaithersburg, Maryland 20899, United States of America; § Material Measurement Laboratory, National Institute of Standards and Technology, Gaithersburg, Maryland 20899, United States of America

**Keywords:** chemometrics, plasticizers, polymers, time-gated Raman spectroscopy, mass spectrometry

## Abstract

Quantitative chemical characterization of postconsumer plastics is challenging because of the large number of polymer chemistries and additives used in formulating plastic products. Plasticizers are an important family of additives used to lower the glass transition temperature and enhance flexibility in polymer formulations. However, their identification and quantification are difficult in recycling and end-of-life processes. This study critically assesses two analytical techniques for their suitability in plasticizer identification: mass spectrometry (high resolution, selectivity, and sensitivity) and time-gated Raman spectroscopy (nondestructive, quicker, and minimal sample preparation). Here, we generate searchable libraries for the identification of 91 common plasticizers using these two techniques, along with the development of reliable tools to measure their spectral similarity. We then analyze the reliability of identifications based on time-gated Raman and/or liquid chromatography–mass spectrometry with atmospheric pressure chemical ionization, explicitly highlighting the advantages and disadvantages of each technique for a given plasticizer chemical family. We have applied these tools to identify and quantify plasticizers in a NIST Standard Reference Material (SRM 2860, Phthalates in Polyvinyl Chloride).

## Graphical abstract

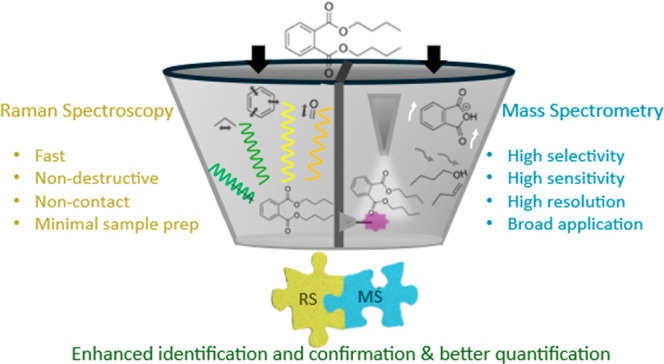

## 1 Introduction

Plastics are highly complex and variable materials, with one report identifying over 10,000 unique chemical substances (e.g., monomers, additives, process aids) actively used in plastics production.^1^ This complexity compounds when considering the recovery, separation, and reprocessing of postconsumer plastics and the characterization of degraded materials in mismanaged plastics. Characterization of plastic composition is relevant for sortation during recycling, quality assurance, and understanding plastics leaked into the environment. Several analytical techniques are used for assessing plastic composition such as near-infrared (NIR) spectroscopy and mass spectrometry (MS). NIR is commonly used to identify polymer type during recycling processes. However, in vibrational spectroscopy techniques, like NIR, the presence of plastic additives can result in crowded, overlapping spectral features that can be difficult to deconvolute and can hinder polymer identification. Certain additives, like pigments, can also undergo competing phenomena during spectroscopy, such as fluorescence and adsorption, which can overwhelm the desired signal and/or limit outgoing light via scattering and emission processes. MS is commonly employed for more granular chemical composition assessment, particularly of small molecules and additives.^2,3^ When identifying polymer composition in environmentally recovered plastics, a suite of techniques including Raman spectroscopy, Fourier-transform infrared (FTIR) spectroscopy, and thermal analysis are often used in tandem to overcome challenges in identification as a result of degradation and fluorescent pigments.^4–6^ There is a need for approaches that enable accurate plastic composition characterization at higher-throughput and lower cost.^7^

A particular challenge is a lack of accessible spectral data that adequately addresses the needs of industry and researchers. Agnostic of analytical technique, there tend to be two types of open databases: (1) pure components (e.g., homopolymer, plasticizers, pigments, etc.)^8–10^ and (2) postconsumer and found objects (e.g., marine debris, lab-weathered samples, assorted postconsumer packaging, etc.).^11–15^ While both types of databases have their uses, pure-component spectral libraries offer greater opportunities for characterization beyond bulk-polymer identification. However, currently there is limited availability of high-quality, pure-component databases that cater to polymer-relevant additives and chemicals of concern, hindering the advancement of chemometric and nontargeted analysis for polymeric systems. The limited availability of such centralized databases contributes to duplication of effort, consuming research time and resources. We also note that generating clean and reproducible spectra of “pure” homopolymers presents more of a challenge than small molecule compounds due to their inherent structural diversity (e.g., molecular mass, dispersity, crystallinity) and the necessity of adding stabilizers and other additives during synthesis. Beyond molecular structure, other aspects of polymer samples can significantly impact spectral features depending on the analytical technique applied.^16–18^ For example, in vibrational spectroscopy, measurements are sensitive to surface properties that impact interactions with incident light such as form factor (nurdle, powder, fragment, fiber, foam) and surface roughness.

This paper will first compare the emerging technique of time-gated Raman spectroscopy with the more established hyphenated liquid chromatography– mass spectrometry with atmospheric pressure chemical ionization (particularly LC-APCI-timsTOF MS/MS) for plastic composition analysis and their potential for analyzing plasticizers in plastics. Next, a study of two parallel libraries of 91 common plasticizers applying both techniques will be described and the results discussed. Finally, we applied these tools to identify plasticizers in a NIST Standard Reference Material (SRM 2860, Phthalates in Polyvinyl Chloride) and provided a perspective on the advantages and disadvantages of each technique and their combined usage.

## 2 Background

### 2.1 Time-Gated Raman Spectroscopy

Raman spectroscopy (RS) is a vibrational spectroscopy technique that measures the energy of inelastically scattered photons resulting from monochromatic incident light inducing a change in the virtual energy states of sample molecules (e.g., rotational, vibrational, and electronic energy states). For a sample to be Raman-active, it must be able to undergo a change in electric-dipole polarization. This contrasts with infrared (IR) spectroscopy, which depends on a change in the electric dipole moment. Therefore, molecules that are strongly Raman active are often weak in IR and vice versa. Due to this distinction, Raman spectroscopy excels in the measurement of molecular features such as electronically neutral bonds (e.g., C–C, C–H, C=C) and aromatic rings which are common in polymer and plasticizer structures.

Herein, we demonstrate time-gated Raman Spectroscopy (TGRS)^19^ as an advanced analytical technique that shows promise in characterizing samples with moderate fluorescence.^20^ TGRS is a time-resolved method that captures the evolution of spectral signal over several nanoseconds. In principle, this allows the temporal deconvolution of the near-instantaneous Raman scattering from the longer time-scale fluorescence emission, where lifetimes can differ by up to 2 orders of magnitude. By using a pulsed laser and carefully controlling the delay and width of the detection gate, TGRS can significantly suppress fluorescence, ambient light, and even thermal emission, yielding a much clearer Raman spectrum with an improved signal-to-noise ratio.

TGRS offers significant advantages for analyzing plasticizers and polymers, especially in challenging real-world scenarios, such as the fluorescence suppression in pigmented and weathered plastics.^20^ TGRS greatly improves our ability to identify polymers and their additives in the presence of moderate fluorescence, which is often difficult with conventional Raman techniques. Common black pigments such as graphite and sulfur black cause significant interference to Raman spectroscopy through the competing effects of fluorescence^21^ and strong absorbance of the incident light. We have observed moderate success in characterizing such samples using TGRS (unpublished). On the other hand, Raman spectroscopy is inherently highly specific, providing unique “fingerprints” for different polymers and their blends. TGRS is valuable for identifying and characterizing recovered plastics from various environments (e.g., marine litter) and has exciting potential for industrial applications such as quality assessment, recycling sortation, and characterization of environmental samples.

An intrinsic limitation of vibrational spectroscopy techniques is the handling of complex chemical mixtures. In principle, the Raman spectra of a sample should be a linear combination of the individual component spectra weighted with considerations toward the relative amount of each component, Raman scattering cross-section,^22,23^ and impacts from chemical interactions (e.g., van der Waals, π–π stacking) that may shift or suppress bands. Various chemometric methods are used to effectively deconvolute the spectra of simple mixtures.^22,24^ However, challenges remain for deconvoluting more complex systems that may have several component species as well as fluorescence contributions. In the following discussion, we differentiate between RS as a class of techniques and TGRS to distinguish aspects of the results that are unique to the capabilities of TGRS compared to results that may feasibly be replicated on a continuous wave Raman instrument.

### 2.2 Mass Spectrometry

Mass spectrometry (MS) is a powerful analytical technique used to identify and quantify molecules on the basis of the mass-to-charge ratio (*m*/*z*) of their ions.^25^ Its application to plasticizers and polymer additives has revolutionized our understanding of these materials, offering detailed insights into their composition, structure, degradation, and loadings.^26^ At its core, MS involves three main steps: (1) ionization: the sample molecules are converted into charged ions. This is a crucial step as it determines which types of molecules can be analyzed and the nature of the resulting fragments. (2) Mass analysis: the ions are separated based on their *m*/*z* ratio. Different mass analyzers (e.g., quadrupole, time-of-flight (TOF), ion traps, Fourier transform ion cyclotron resonance (FT-ICR)) achieve this separation through varying electric and/or magnetic fields. (3) Detection: Separated ions are converted into a measurable signal by detectors such as electron multipliers, Faraday cups, or scintillation detectors, and their abundances are recorded, generating a mass spectrum depicting ion abundance versus *m*/*z*. In addition, the mass analyzers may include multistage ion separation known as tandem mass spectrometry (MS/MS or MS^*n*^), which involves multiple, sequential stages of ion isolation, fragmentation of mass-selected ions in a buffer gas, and analysis to improve structural identification and reduce interference. These mass spectra provide a unique “fingerprint” for identification by comparing it to reference mass spectral libraries.^27,28^

MS is usually coupled through hyphenated techniques that provide different types of information and are suitable for different sample types, including gas and liquid chromatography, ion mobility, and others. Because plasticizers are often volatile or can be made volatile through derivatization, GC–MS (gas chromatography–mass spectrometry) is excellent for both qualitative identification and quantitative analysis of volatile small-molecule plasticizers. For less volatile or thermally labile plasticizers, LC–MS (liquid chromatography–mass spectrometry) is the preferred method. LC separates the plasticizers, and the eluent is then introduced into the mass spectrometer for detection. This is particularly useful for analyzing plasticizer mixtures or those with higher molecular masses. A more in-depth discussion of other relevant MS techniques as they apply to plasticizers and polymer samples is presented in the Supporting Information (SI IX).^29^

### 2.3 Plasticizers

Plasticizers are substances added to a range of products to make them softer, more flexible, and easier to process. They can also provide other properties such as temperature stabilization (e.g., trimellitates) and flame retardancy (e.g., organophosphates). Plasticizers have been designed with a range of structures to meet the miscibility needs of diverse polymer chemistries and to facilitate the requirements of the polymer industry (e.g., glass transition temperature suppression, low volatility, and cost). Modification in chemical structure impacts both their functionality and compatibility with different polymers.

Phthalate esters are among the most widely used plasticizers, accounting for over 55% of global plasticizer consumption in 2020.^30^ They are especially common in the preparation of poly­(vinyl chloride) (PVC), where they can account for upward of 50% by mass.^30^ However, some phthalates have raised health and environmental concerns, leading to restrictions in their application and production.^31–33^ Nonphthalate plasticizers are gaining popularity as theoretically safer alternatives, although toxicology studies are often limited or absent for plasticizers that are not expected to come into immediate contact with food or children’s products. Readers who are interested in the types of applications and formulations using different plasticizers are directed to Wypych 2023 ^30,34^ and the International Council of Chemical Associations (ICCA) Plastic Additives Database.^35^

Many plasticizers share similar chemical moieties, such as aliphatic pendant chains, ester groups, and aromatic rings, which respectively increase the free volume between polymer chains, contribute to compatibility through hydrogen bonding and van der Waals interactions, and enhance compatibility through induction forces and π-electron displacement.^36,37^ Structurally, plasticizers can be categorized along two axes: esters versus nonesters, and aliphatic versus aromatic structures. From an analytical perspective, it is useful to group plasticizers by their core structural units (e.g., phthalates, adipates, phosphates), as compounds with similar structures tend to exhibit similar properties and yield similar spectral signatures.

Figure 1 introduces a general overview of plasticizer chemical structures included in this work. The presented structures are generalized to facilitate explanation and reading of the results. Exact representation of the aliphatic ester di (2-ethylhexyl) adipate (DEHA); aromatic ester, di (2-ethylhexyl) phthalate (DEHP); and a nonester plasticizer, tricresyl phosphate (TCPh) are presented in Figure 2 along with their atmospheric pressure chemical ionization (APCI) MS and TGRS spectra. Detailed lists of the plasticizers and their structures are available in Tables S2 and S3 respectively.

**Figure 1:**
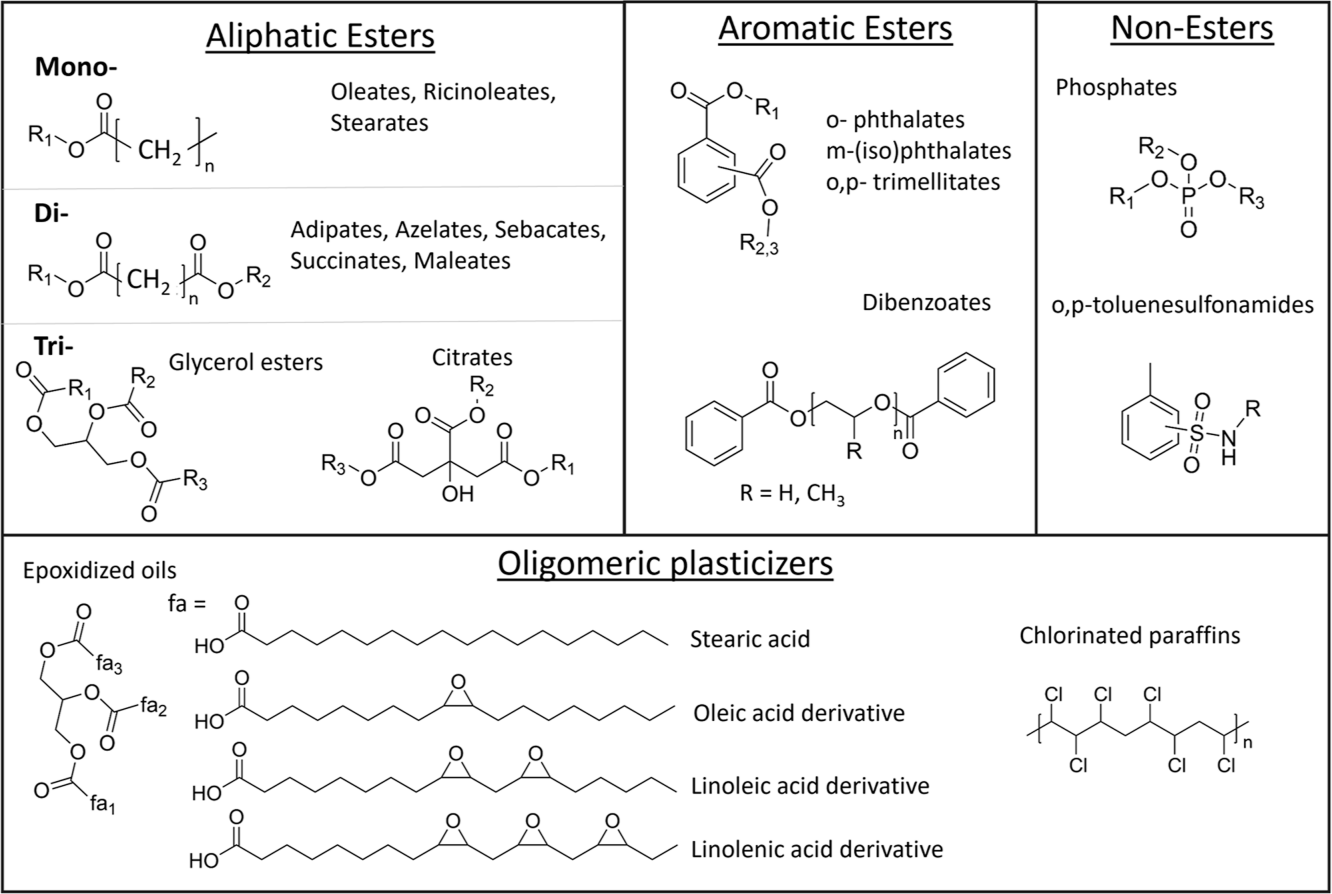
Major plasticizer families included in this publication.

**Figure 2:**
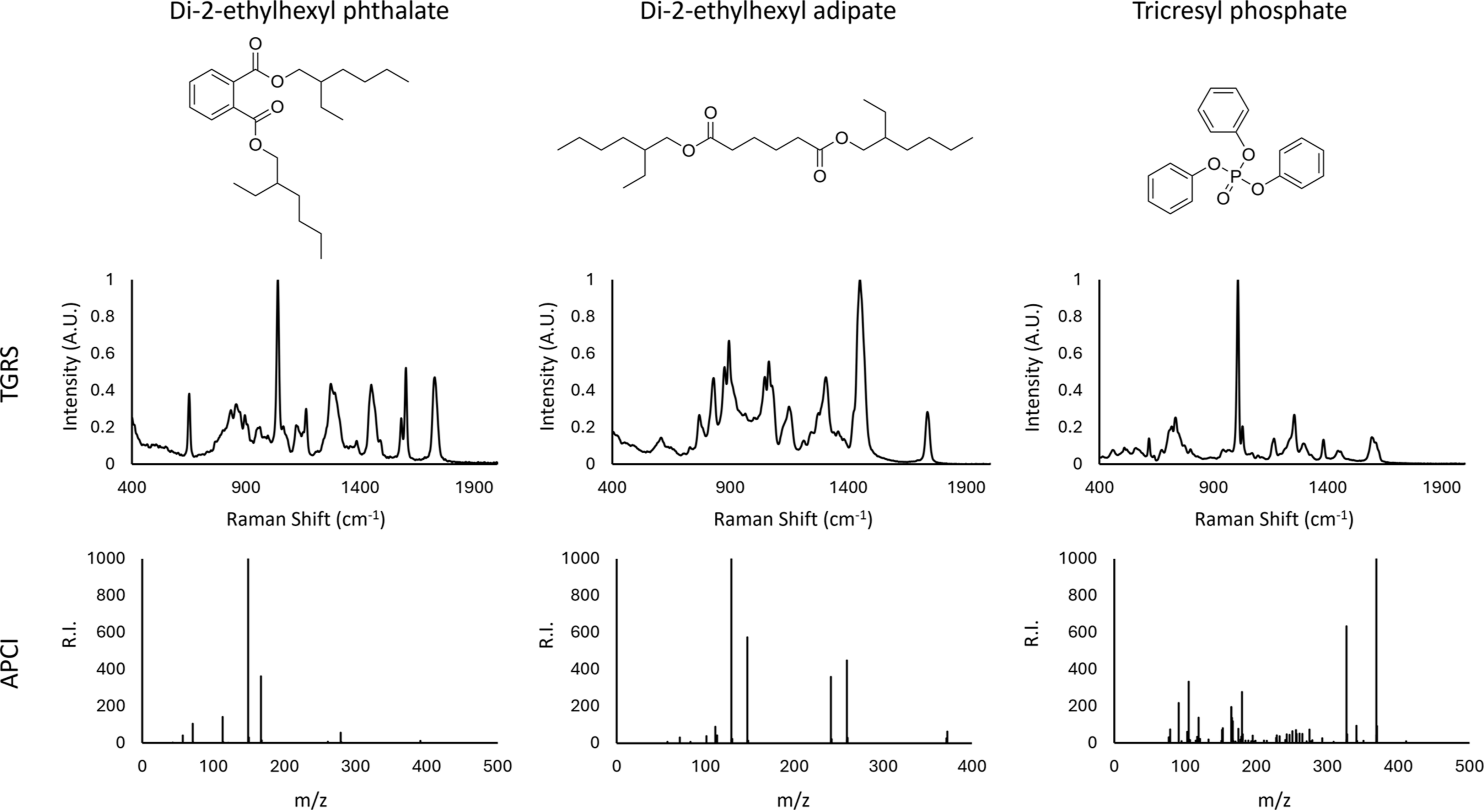
Examples of three plasticizers illustrating three major categories, depicting their structures, time-gated Raman and APCI–LC–MS/MS spectra. The axis R.I. refers to relative intensity.

### 2.4 Raman Spectroscopy and Mass Spectrometry for the Analysis of Plasticizers

MS methods are widely used for industrial plasticizer analysis^3^ but are not suitable for in-line applications. While vibrational spectroscopy has been applied for the identification and semiquantification of plasticizers in polymer extracts,^2,3^ especially at higher plasticizer loadings,^38,39^ these techniques are not widely used. Herein, we propose RS as an attractive technique for direct analysis of polymer specimens due to its comparatively rapid, nondestructive, noncontact nature, and capacity to measure through optically transparent container walls. TGRS in particular stands out for applications in process analytical technology for its additional fluorescence suppression and insensitivity to ambient light.

While both Raman and MS are common techniques in the polymer analysis toolbox, formal comparison between the two are rare.^5,40,41^ We identified a range of application-based comparison studies performed in diverse fields such as museum studies,^42^ forensics,^43^ microplastics,^5^ biology,^40,41,44^ and food quality assurance.^45^ These studies largely focus on leveraging MS as a higher-resolution technique to assess and validate RS as a faster, cheaper, nondestructive, and more portable alternative. Collectively, these studies found that Raman methods were generally effective at performing the desired measurements.

## 3 Materials and Methods

### 3.1 Materials

This work used a collection containing 91 common plasticizers from Scientific Polymer Products Inc.[Fn fn1] A list of the plasticizers is given in Table S2 of the Supporting Information.

Water and acetonitrile (HPLC–MS grade) with 0.1% formic acid were purchased from Honeywell-Burdick & Jackson (Muskegon, MI, USA).

NIST standard reference material (SRM) 2860 was used as a reference sample for the application of the respective libraries. SRM 2860 consists of three samples of shredded PVC with varying phthalate plasticizer loadings. The SRM certificate also indicates the inclusion of an unspecified amount of bis­(2-ethylhexyl) adipate (DOA) that was used to disperse the phthalates within the PVC during processing. The jars are labeled as BLANK containing trace residues of bis­(2-ethylhexyl) phthalate (DEHP) and a volume of DOA; level I containing approximately 0.1 wt % each of di-*n*-butyl phthalate (DBP), benzyl butyl phthalate (BBP), diisononyl phthalate (DINP), and diisodecyl phthalate (DIDP) for a total of ≈0.5 wt % phthalate loading; and level II containing approximately 2 wt % each of DBP, BBP, DEHP, DINP, DIDP, and di-*n*-octyl phthalate (DNOP) for a total of ≈10 wt % phthalate loading. For the purpose of this paper, the control blank will be referred to as “BLANK”, level I will be called “0.5%”, and level II will be called “10%”.

### 3.2 Time-Gated Raman Measurements

TGRS measurements were collected using a PicoRaman spectrometer (Timegate Instruments[Fn fn1]) using a 532 nm pulsed laser with an average power of 10 mW, and a time duration of (13–39) s or (100–300) subacquisitions in the companion software. The spectrometer was coupled via a fiber-optic probe, and measurements were collected in an enclosed chamber secured by an interlock system. Liquid samples were measured in aluminum sample pans with ≈2 mm of liquid depth to the bottom of the pan. Solid and powder plasticizer samples were compacted in a pellet press to yield a smooth, continuous surface. For select plasticizers with poor signal-to-noise ratios (SNR), subacquisition and power level settings (8–15) mW were varied to maximize signal. Specific collection conditions for each spectra as well as the raw and processed data can be accessed in the Supporting Information (Table S4) and the NIST public data repository,^10^ respectively. A polystyrene sample was used to calibrate the wavenumber range between measurement sessions (Supporting Information V).^46^

### 3.3 Time-Gated Raman Library Building

The Raman library was made by collecting multiple spectra of each plasticizer following the procedure outlined above. The number of spectra taken of each compound were typically in the range of 5–20 measurements, to optimize the collection conditions (e.g., working distance, laser power, and collection time) for a maximum signal-to-background-ratio (SBR) (Supporting Information V). The Raman bands of the pure plasticizer species were generally consistent between runs; however, the intensity of the background noise and the relative contribution of the fluorescence varied. The two spectra with the highest SBR for each plasticizer are included in the Raman library to allow calculation of the signal-to-noise-ratio (SNR). For samples with comparatively low SNR (<50), three spectra are included to provide greater detail on the variability of sample noise. The library includes for each plasticizer: (1) the unprocessed TGRS spectra consisting of 3-dimensional (3D) spectral data with axes: normalized intensity, Raman shift (cm^–1^), and time (ns); and (2) processed data consisting of flattened 2D spectra (Raman Shift, time) obtained by averaging spectral data between (5.5 and 5.8) ns of detector time. The data was processed and visualized in Python JupyterLab, and the processing code is available in the companion GitHub repository to facilitate processing of the data under different time-gating conditions (e.g., modulating fluorescence contributions in the calculated spectra).^47^

### 3.4 Mass Spectrometry Measurements

For tandem mass library building, we utilized LC–APCI–MS/MS in a Bruker timsTOF Pro mass spectrometer equipped with a VIP-HESI-APCI electrospray source (Bruker, Billerica, Massachusetts). Most plasticizers are liquids, and solutions were prepared at micromolar concentrations. Typically, 20 μL of the plasticizer were pipetted and dissolved in methanol/water/formic acid (98:2:0.1, *v*/*v*/*v*), yielding concentrations of 50–250 μM. Plasticizers ionize very efficiently, and these low concentrations for the mass spectral library avoid both, signal saturation and instrument contamination. Tandem mass spectra were acquired in the positive mode. We use a 100 μL sample loop and a small C18 cartridge in the flow path to increase back pressure and ensure infusion experiments are long enough to yield a substantial number of spectra for creating a consensus spectrum; otherwise, ionization may be poor. The APCI tandem mass spectral library can be accessed through the NIST public data repository.^48^ The MS/MS fragmentation spectra were measured at different collision energies with a cutoff of 5000 counts. The timsTOF resolution for MS/MS was 50,000. N_2_ gas (99.999%) was used as the collision gas. A comprehensive description of MS settings is provided, along with Supporting Information (Table S6).

Quantitation experiments for NIST SRM 2860 were performed by dissolving the polymer in tetrahydrofuran (THF) and precipitating it with *n*-hexane. The THF/hexane solutions after centrifugation and separation of the solid polymer were analyzed using LC–MS gradient separations using reversed-phase chromatography, an ACQUITY UPLC CSH C18 column, 130 Å (13.0 nm), 1.7 μm, 2.1 mm × 100 mm, and mobile phase A: water and mobile phase B: acetonitrile (both containing 0.1% formic acid). The chromatographic separation has been adapted from previous methods.^49,50^ The timetable and other LC and MS settings are given with the Supporting Information (SI XIII).

### 3.5 Mass Spectrometry Library Building

The usual tandem mass spectrometry library building procedures require, but are not limited to, acquiring multiple spectra of the plasticizer, usually dozens or hundreds, to generate a consensus spectrum^51^ from a sample of the pure compound. In the current MS/MS library, most nonsmall-molecule plasticizers were excluded because they are mixtures that yield contaminated MS/MS spectra (e.g., chloroparaffins and epoxidized oils). We have also supplemented the APCI plasticizer library with spectral data already present in the NIST Mass Spectrometry Data Center library including electrospray ionization (ESI) data where available and spectra of other plasticizers and relevant precursors that were not included in the Scientific Polymer Products Inc. kit to discuss fragmentation trends more effectively. The fragmentation pattern of the consensus spectrum was examined using in-house tools, such as the MS interpreter (see Supporting Information VII), to identify unassigned peaks, meaningless neutral losses, and other spectral artifacts that would be used to accept or reject its inclusion in the library. Figures S1−S3 of the Supporting Information illustrate a simplified scheme of the experimental and data-processing procedures employed in this work.

After these procedures, the spectra were manually interpreted to understand the fragmentation mechanisms. Finally, the spectrum and all related metadata, such as name, synonyms, structures, etc., were included in a library using in-house software with the NIST format and managed using the NIST browser, MS Search. These libraries can be modified using the browser, specifically for adding or deleting spectra. The MS search browser can also be used for building new spectral libraries. Software and manuals can be downloaded for free from the NIST Mass Spectrometry Data Center webpage (Supporting Information VII).^49^ To use the library, the user must follow the instructions in the manual, install the MS Search browser on their computer, and add the library to the “MSSEARCH” folder. The browser allows multiple identity and similarity searches. For this work, the most relevant are Identity: MS/MS and similarity: MS/MS hybrid,^52^ both of which are easily accessible from the library search tab of the browser (see Supporting Information for details).

## 4 Results and Discussion

### 4.1 Raman and MS Analysis by Plasticizer Family

Molecules with similar structural features tend to exhibit similar spectral features, which can assist in their identification. Since Raman spectroscopy directly investigates the structural connectivity of molecules, we identified a strong “family resemblance” between plasticizers with the same core functional features. A schematic mapping of characteristic Raman spectral features is presented in Figure 3 showing how characteristic bands vary across the different chemistries, highlighting strong similarities both within core functional group families (e.g., adipates), and across families with similar chemistries in the core functional group (e.g., aliphatic esters) (Figure 1). On the other hand, the fragmentation patterns of plasticizers during tandem mass spectroscopy are relatively straightforward to interpret due to their simple chemical structures, which mainly consist of a polar segment with attached hydrocarbon chains and the absence of complex rearrangement reactions. In the case of esters, the breaking of the ester bond determines their fragmentation.

**Figure 3:**
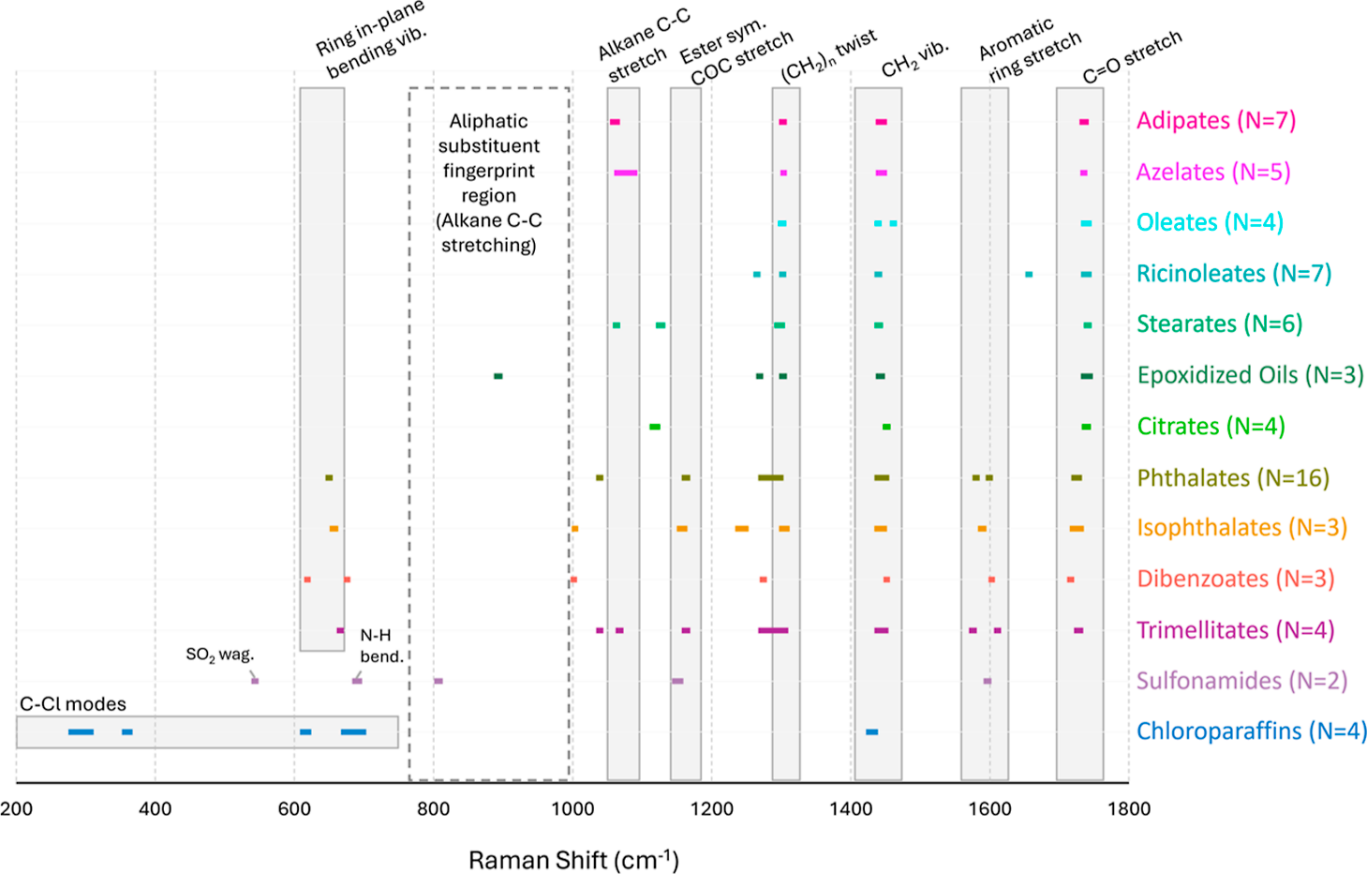
Schematic of Raman band ranges as a measure of peak location for peaks common across each spectral family. *N* values (e.g., (*N* = 7)) indicate the number of species used to inform the range. Gray boxes indicate the general range of each vibrational band indicated.

Below, we introduce the common categories of plasticizer families considered in this study. We also consider the characteristic analytical features of each family with respect to both Raman spectroscopy (RS) and mass spectrometry (MS). For brevity, the full discussion of spectral characterization is only included for the first plasticizer family (Oleates) and the remainder can be found in the Supporting Information (SI X). Raman peak assignments were assigned based on Socrates.^53^ While a TGRS instrument using a 532 nm excitation was used to collect the described data set, the plasticizers tended to have minimal to moderate fluorescence and should be generally replicable on a conventional, continuous-wave Raman. Families that demonstrate elevated fluorescence are indicated in the appended resources, and a qualitative description of fluorescence is included in Table S7 alongside characteristic peak assignments for each family of plasticizers.

#### 4.1.1 Aliphatic Esters

The most numerous category of plasticizers in this study are the aliphatic esters derived from mono-, di-, and trisubstituted organic acids. Common plasticizers in this group include the monoesters: oleates and stearates; diesters: adipates and azelates, and triesters: citrates as well as glycol and glycerol esters. We also note that many of the precursor monoalkyl carboxylic acids are primary components in oils frequently used as epoxy fatty acid plasticizers (section: Nonesters). These include linoleic acid, oleic acid, stearic acid, and palmitic acid.^34^

Example of spectral overview analysis developed for each plasticizer family:

Oleates (*N* = 4) are the esters of oleic acid (C_18_H_33_O_2_). It is classified as a monounsaturated omega-9 fatty acid.

RS: the primary Raman peaks associated with oleates include (CH_2_)_*n*_ twisting (1299–1303) cm^–1^, CH_2_ vibration (1438–1441) cm^–1^, *cis*-dialkyl alkenes C=C stretching (1653–1657) cm^–1^, carbonyl stretching (1735–1742) cm^–1^. Where the specified range indicates the range of peak-maximum locations observed across the oleate plasticizers included in the library. The presented peaks are shared across all plasticizers in the family, while unique peaks were excluded. Unique peaks are associated with substituent chemistries and are generally found in regions associated with C–C, CH_2_, and aromatic bond modes.

MS: in tandem mass spectrometry, the fragmentation of oleates depends on the size of the alkyl chain of the alcohol. In short chains, such as those found in methyl and ethyl oleate, the most intense peaks result from the breaking of the ester bond, producing acylium ions at *m*/*z* 265 and the loss of the alcohol. The acylium ion undergoes a subsequent loss of water, producing a peak at *m*/*z* 247. If the alcohol chain is larger, the oleate usually also undergoes loss of the alkyl chain of the alcohol, producing a peak of protonated oleic acid at *m*/*z* 283. These peaks are present at very low collision energies. At higher energies, multiple peaks related to the fragmentation of the oleic acid chain appear that are common to most oleates, e.g., peaks at *m*/*z* 223, *m*/*z* 177, *m*/*z* 163, *m*/*z* 135, and many others. However, none of these peaks pinpoint directly to the position of the unsaturation, which requires special techniques if necessary.

For in-depth spectral analysis of the remaining plasticizer families covered within this work, please refer to Supporting Information X. A tabulated presentation of Raman peak assignments by family is also provided in Supporting Information XI.

#### 4.1.2 Aromatic Esters

Aromatic esters are the most commonly applied category of plasticizers largely as a result of the prevalent use of phthalates (*ortho*-phthalic derivatives) in PVC. Other common aromatic ester plasticizers include isophthalates (*meta*-phthalic acid derivatives), trimellitates (1,2,4-tri ester substituted benzene), and dibenzoates.

Within the investigated *ortho*-phthalates, we highlight two outliers as representatives for general outlier trends observed across the different plasticizer categories: notably, deviations in spectral trends with room temperature solids and methyl substituted plasticizers. Figure 4 depicts a cosine similarity heatmap, visualizing similarity between species in the library. While most included samples exist as room temperature liquids, several compounds exist as solids at room temperature and were measured in their solid form. These compounds are marked with an asterisk (*) in Figure 4. We also observe that across plasticizer categories and families, many of the methyl-substituted species exist as room temperature solids. In the case of the room temperature solid, dicyclohexyl phthalate, it has a blue-shifted carbonyl peak at 1715 cm^–1^, and numerous sharp, medium intensity bands in the range (200–1430) cm^–1^. It should be noted that Raman spectra of compounds will vary based on form factor.^24^ For example, cyclohexyl phthalate, as a room-temperature crystalline solid, will likely differ from the spectral contributions of cyclohexyl phthalate as a room-temperature amorphous constituent dissolved in a solid substrate.

**Figure 4:**
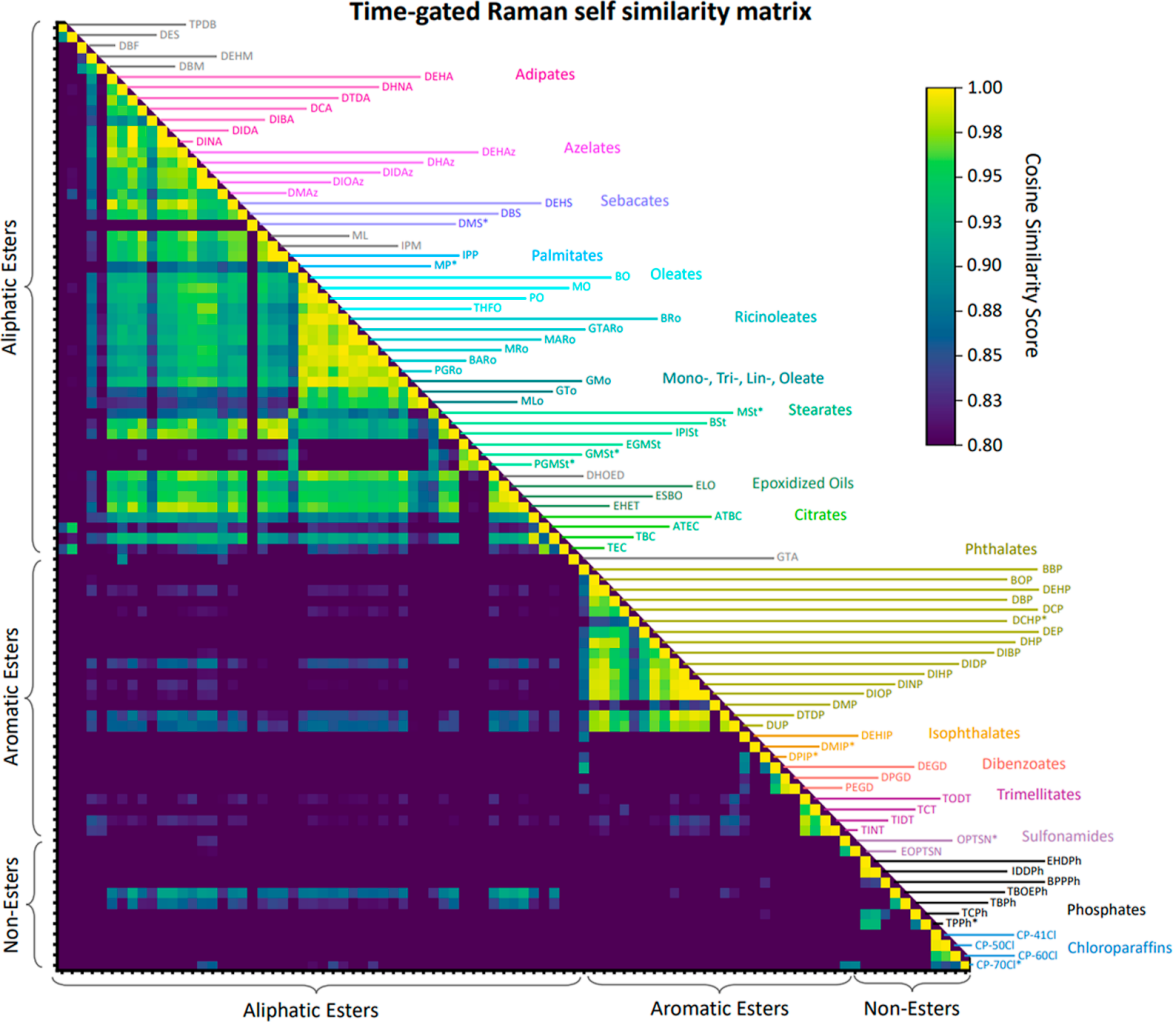
Time-gated Raman plasticizer library self-similarity matrix. A score of 1 indicates identical spectra and a 0 means they are orthogonal. Asterisks (*) indicate samples measured in a solid state. Scale bar was selected to maximize contrast.

#### 4.1.3 Nonesters

Nonester plasticizers include organophosphates, sulfonamides, and the oligomeric plasticizers, chloroparaffins, and epoxidized oils. Many of these species fall into the category of secondary plasticizers incorporated in conjunction with a primary plasticizer(s) to achieve a variety of effects including low temperature flexibility, cost reduction via dilution, flame retardancy, and improved temperature/UV stability.^30,34,54^

Organophosphates and chloroparaffins stand out on this list for their flame-retardant properties. In the case of organophosphates, there is a strong deviation in both the bestowed properties as well as spectral trends between aryl and alkyl substituted species. Namely, aryl-phosphates provide flame retardant properties and negatively affect plasticization, while alkyl-phosphates are poor flame retardants, but offer good low temperature plasticization.^34^ We note that peaks associated with the core phosphate structure are weak in Raman scattering, with spectra largely dominated by the alkyl/aryl pendant chemistries. Chloroparaffins on the other hand, stand out as oligomeric, flame-retardant plasticizers that are regulated in some regions for their documented toxicity and bioaccumulation.^55–57^ They are still generated in high volume and used for applications such as flame retardants, metallurgy, and some plastics applications (e.g., PVC, PU, and rubbers).^34,55^ The chloroparaffin samples yielded significant fluorescence interference under Raman measurement, with the 70% chloride sample exhibiting the most fluorescence interference among all samples in the library.

In the present APCI–LC–MS/MS library, the oligomeric plasticizers are excluded. Tandem mass spectral libraries are usually restricted to small molecules, however, this does not mean that mass spectrometry cannot be used to analyze oligomeric plasticizers. In fact, MS is commonly used for characterizing macromolecules to determine features such as molecular mass distributions (of both lower and high-molecular mass polymers),^29,58,59^ end groups, and characterizing repeating monomer units. However, applying it to complex mixtures of natural or industrially compounded polymers requires component separation work that is beyond the purpose of this paper, and thus excluded from the MS plasticizer data set.

### 4.2 Spectral Similarity Analysis

The use of spectral similarity metrics to determine the identity or composition of a query sample against a reference library is common across many different types of instrumentation and measurements. In these methods, a range of algorithms are applied including correlation-based methods (e.g., cosine similarity, Spearman’s rank correlation, Pearson correlation), Euclidean metrics, and multivariate techniques such as principal component analysis (PCA).^60^ Herein, cosine similarity (CS) calculations (eq 1) were performed to assess both the MS and Raman data sets, since it is the algorithm applied within the NIST Mass Spectral Library. TGRS calculations were performed using preprocessed data (calibration, background correction, normalization) using an unweighted CS calculation.

MS similarities were calculated using a weighted dot product CS calculation developed for this paper and described below. First, each abundance (*I*) is transformed into a “weight” of the type *w* = (*m*/*z*)^*m*^ *I* ^*n*^, where *n* = 1/2 and *m* = 0 resulting in *w* = *I* ^1/2^. Let **a**_**L**_ and **a**_**U**_ represent “vectors” of the library and unknown weights. Their magnitudes are used to normalize the result to a value between 0 (orthogonal) and 1 (colinear). Then, let **m**_**L**_ and **m**_**U**_ denote equal-length lists of weights for the subset of peaks returned by the matching algorithm^52,61^

1 $$\cos\theta =\frac{m_{L}\cdot m_{U}}{∥a_{L}∥∥a_{U}∥}=\frac{\sum_{match}w_{L}w_{U}}{\sqrt{\sum_{\text{all}}w_{L}^{2}}\sqrt{\sum_{\text{all}}w_{U}^{2}}}=\frac{\sum_{match}I_{L}^{1/2}I_{U}^{1/2}}{\sqrt{\sum_{\text{all}}I_{L}}\sqrt{\sum_{\text{all}}I_{U}}}$$

Equation 1 is used for “forward” searches. In a “reverse” search, nonmatching unknown peaks are assumed to be contaminants, so m_U_ replaces a_U_, and the result is closer to unity. In the NIST MS software, the “match factor” (MF) is calculated from cos θ by applying various empirical adjustments and rounding to an integer on a 999-point scale.

An MS/MS identity search uses all peaks in the spectra to compare the query and reference spectra as explained above. For similarity hybrid searches, the parent ion mass constraint is removed, combining conventional, direct peak matching with the logical equivalent of neutral-loss matching.^52^ Figure S3 illustrates the hybrid search of an unknown compound. In the absence of the parent ion mass (*m*/*z* 419) constraint, the algorithm matches a set of phthalates with reasonable neutral losses and assigns a corresponding score. In this case, matching the phthalate core and the delta mass, i.e., the mass difference. Multiple matches to phthalates is a confirmation of the type of compound.

To more directly compare the APCI–MS and TGRS libraries, self-similarity matrices (SSMs) of CS scores were constructed to visualize spectral spread within the respective plasticizer data sets (Figures 4 and 5). The SSM heatmaps demonstrate distinct features of the two methods with respect to individual strengths and prospective use cases. First, Figure 4, depicting the TGRS SSM, shows the strong resemblance not just within individual families of plasticizers, but also across the broader categories of plasticizers with similar chemical features (e.g., aliphatic esters vs aromatic esters). This is indicative of the significant spectral contribution of characteristic Raman bands associated with the core functional units of a given plasticizer, which tend to be consistent across family members.

**Figure 5:**
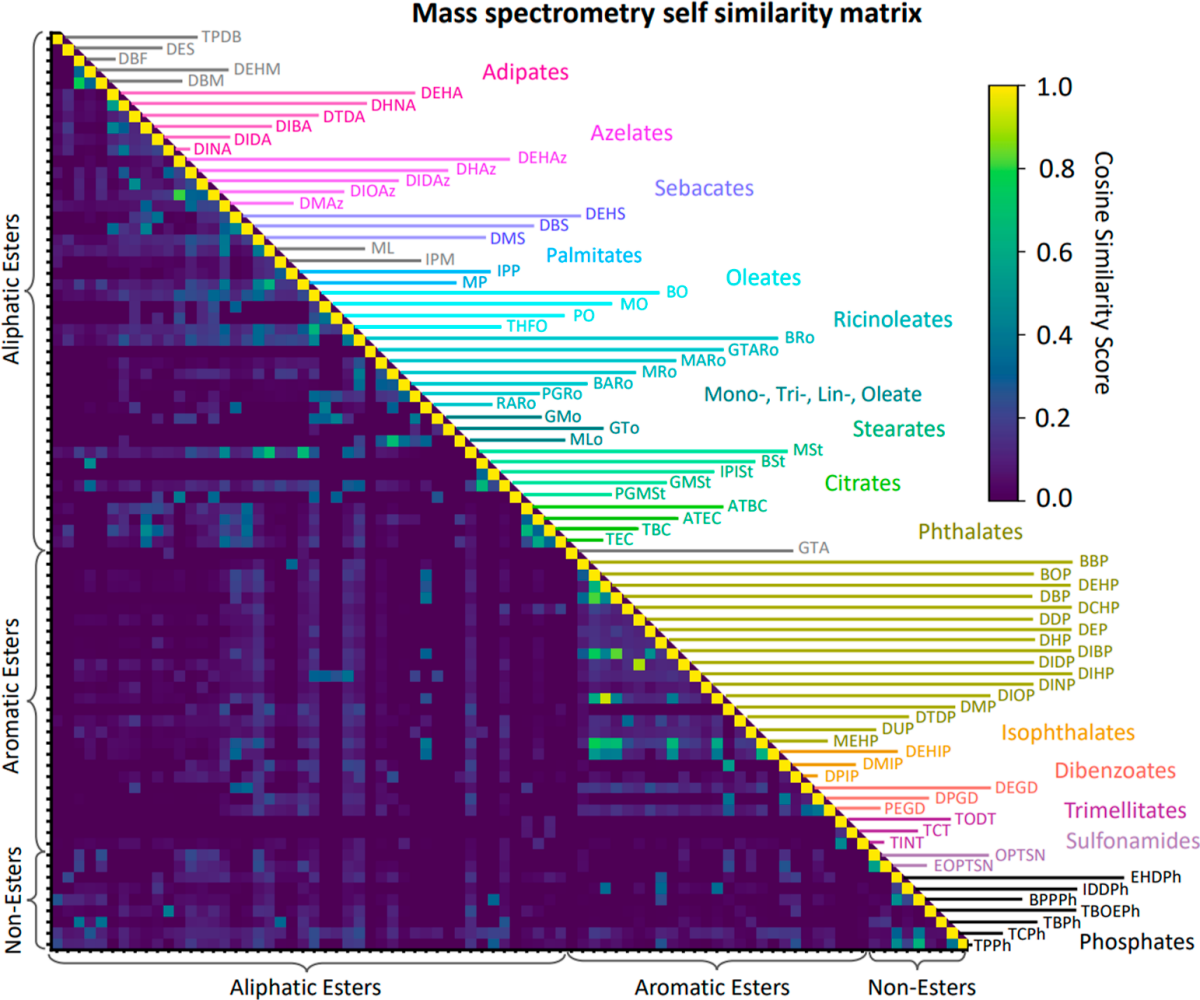
Mass spectrometry plasticizer library self-similarity matrix. A score of 1 means the spectra are identical and a score of 0 means vectors are orthogonal. Note that the scale bar differs from Figure 4.

In contrast, the MS SSM is more similar to an identity matrix, showing the strong uniqueness of each spectrum in the library with only weak association between plasticizers with similar structures (Figure 5). In isolation from a thoroughly populated library that can contextualize fragmentation patterns, it may be challenging to precisely identify the core chemical functionality of a target compound.

While the Raman spectra of each compound in the present library is unique and distinct from all other compounds, the degree of uniqueness varies based on how similar the chemical structures are. Together, Figures 4 and 5 show the strength of MS analytical techniques for exact material identification when adequate libraries are available. However, in the absence of an adequate library of compounds, Raman methods offer a promising opportunity to identify the category or family of plasticizers present. The limited sample preparation required for RS, and rapid data collection times position RS as a useful screening tool.

The measurement versatility of the Raman method compared to MS is also demonstrated through the inclusion of oligomeric plasticizers, such as the chloroparaffins and epoxidized oil families, which are known to be polydisperse and contain isomer mixtures that can be difficult to measure reproducibly using MS.

### 4.3 Application to the Analysis of Plasticizers in Polymer Samples

We have used NIST SRM 2860 to compare the nontarget identification efficacy of the respective methods as well as to test the effectiveness of the libraries in identifying multiple plasticizers in a complex material. This SRM is intended primarily for use in validating methods for determining six phthalate esters in PVC via GC–MS.

For the TGRS analysis of plasticizers contained in PVC SRM 2860, CS was applied to the Raman data for two wavenumber regions: (200–2000) cm^–1^ and (1640–1800) cm^–1^ (Further discussion available in Supporting Information XII). The compared spectra were subject to preprocessing steps including calibration, normalization, and background-subtraction.^22^ The analysis also includes residual spectra of the 0.5% and 10% loadings determined by subtracting the SRM BLANK (nominally pure PVC) from the 0.5% and 10% loadings spectra respectively (Figure S4). Figure 6 depicts a subset of the CS analysis performed along the longer spectral range (200 cm^–1^ to 2000 cm^–1^), comparing the direct SRM spectra and the 0.5% and 10% residuals (Res.) to the phthalates in the library. The CS analysis indicates elevated similarity with phthalate plasticizers compared to other plasticizers in the library, especially in the 10% loading condition and its residual. This suggests the presence of phthalates in the 10% loading condition, as confirmed by the certificate. By isolating the non-PVC peaks in the residual, a strong spectral similarity is observed between the 10% residual and characteristic phthalate bands (Figure S4). The 0.5% residual shows only two bands that are both present in the BLANK and unrelated to plasticizer content. The absence of plasticizer bands in the 0.5% residue is attributed to the plasticizer loading approaching the expected limit of detection of the TGRS as well as a trace presence of DEHP and DEHA in the BLANK (visible as the carbonyl peak at 1730 cm^–1^), which was subtracted off in calculation of the residual. The full library comparison is available in Figure S5.

**Figure 6:**
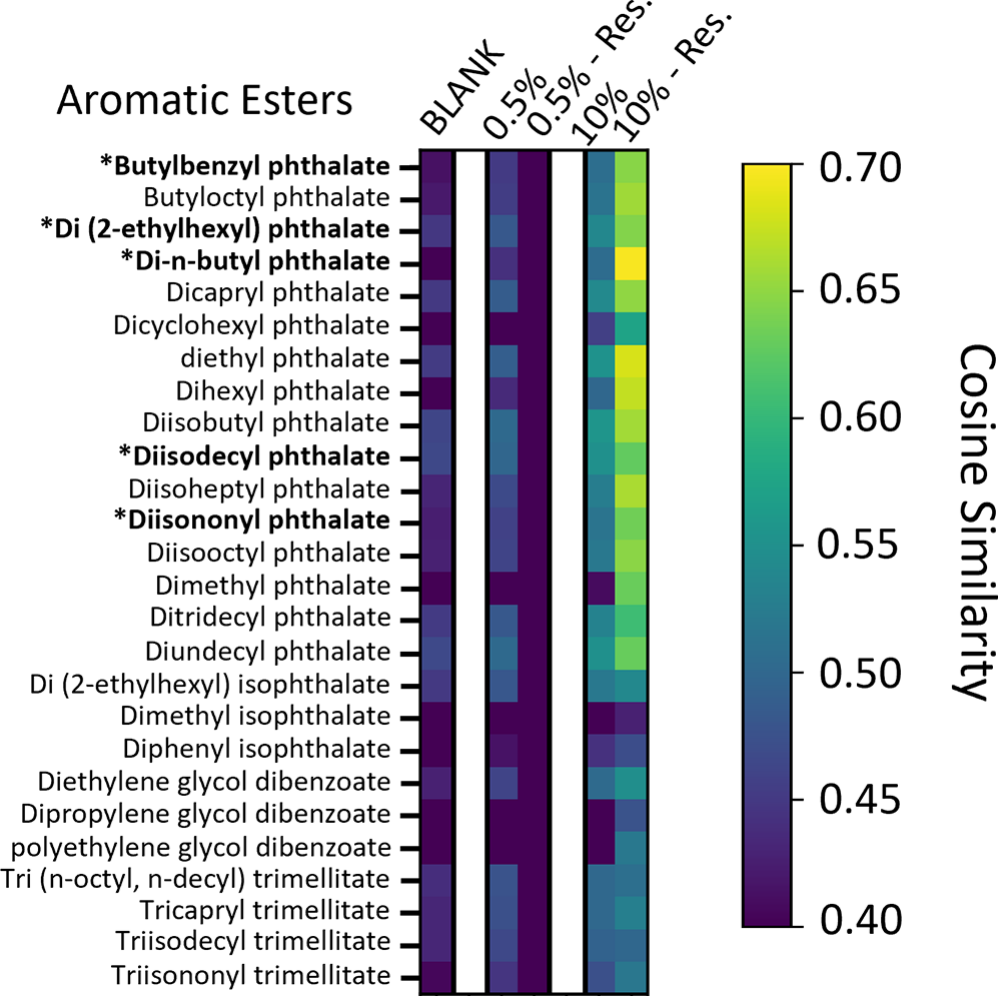
TGRS cosine similarity analysis of PVC SRM 2860 with phthalate plasticizers comparing spectral range (200–2000) cm^–1^. (*) indicates the phthalate is present in the 10% loading, and the (&) indicates the phthalate is present in the 0.5% loading. Di-*n*-octyl phthalate is also present in the 10% loading but not included in the present library.

Performing CS across only the carbonyl band region (1640–1800) cm^–1^ is sufficient to demonstrate high similarity with phthalates, although other plasticizers share similar ranges for carbonyl peak location (e.g., maleates, adipates, and ricinoleates) leading to false positive identification in this limited spectral range (Figures S6 and S7). Similar analysis performed by Nørbygaard and Bergon on PVC-phthalate samples with much higher plasticizer loadings (>20 wt %) demonstrated the capacity for both qualitative and quantitative characterization of generic phthalate peaks based on calibration curves of normalized Raman peak intensities.^38^ In this study we present analysis based only on CS methods. We speculate that applying more advanced chemometrics techniques such as PCA to the TGRS analysis would yield improved results and we are currently exploring such methods.

MS analysis of SRM 2860 was performed on the 0.5% loading sample to demonstrate the qualitative and quantitative capabilities. An extraction-based method was applied by dissolving and reprecipitating the polymer according to the conditions specified in the Materials and Methods section; other details are provided in the Supporting Information. Briefly, a calibration curve was constructed by measuring peaks and their corresponding areas for a set of external standards. Figure S9 shows the extracted chromatogram and the external standards calibration curve for DEHP. To our knowledge, APCI–LC–MS on a timsTOF has not been used for this type of quantitation before. Usually, exact quantitation requires measuring multiple reaction monitoring (MRM) transitions using a triple quad mass analyzer. The dynamic range for quantitation is relatively large, ranging from ng/g to μg/g. We observed a lack of linearity below 1 ng/g as well as at high concentrations, but the latter is irrelevant because the sample can be diluted to a quantifiable level. The quantitation of DEHP yields a value of 1502 μg/g, within the margin of error reported in the Certificate of Analysis.^62^ All the other plasticizers in SRM 2860 were also identified and semiquantified (Supporting Information XIII).

While the identification of plasticizers in PVC via TGRS shows promise as discussed above, there are still some challenges in applying this approach for unknown samples. When the plasticizer and polymer share common structural features, the overlap in spectral features can yield false positives if the entire continuous spectrum is analyzed. In these cases, it may be necessary to limit the spectral range included for analysis. For example, in preliminary work assessing controlled loadings of dilute plasticizers in PDMS, we do not analyze the spectral regions where the polymeric Si–O bands dominate. Alternatively, in the case of polyolefins, false positives may result for plasticizers containing large aliphatic sections because the signal from the C–C backbone of the polymer chain will overlap with contributions from plasticizer backbone and pendant chains. Thus, the spectral regions utilized for identification may need to be informed by both the polymer and plasticizer structure when they share structural features.

### 4.4 Comparing Both Techniques, Advantages, and Disadvantages for the Combined Use

RS and MS show promising benefits as complementary measurement tools whether as sequential or hyphenated techniques.^63^ In Table 1 we compare a range of relevant features of RS and MS in both the practical logistics of performing measurements as well as considerations toward analytical capabilities, precision, and accuracy. As previously identified in the literature, RS techniques offer particular benefits through their minimal sample preparation requirements and comparatively rapid data collection, while being a noncontact, and nondestructive method. In contrast, MS methods excel in measurement resolution, accuracy, and precision, but require substantially more sample preparation and measurement time.

**Table 1:**
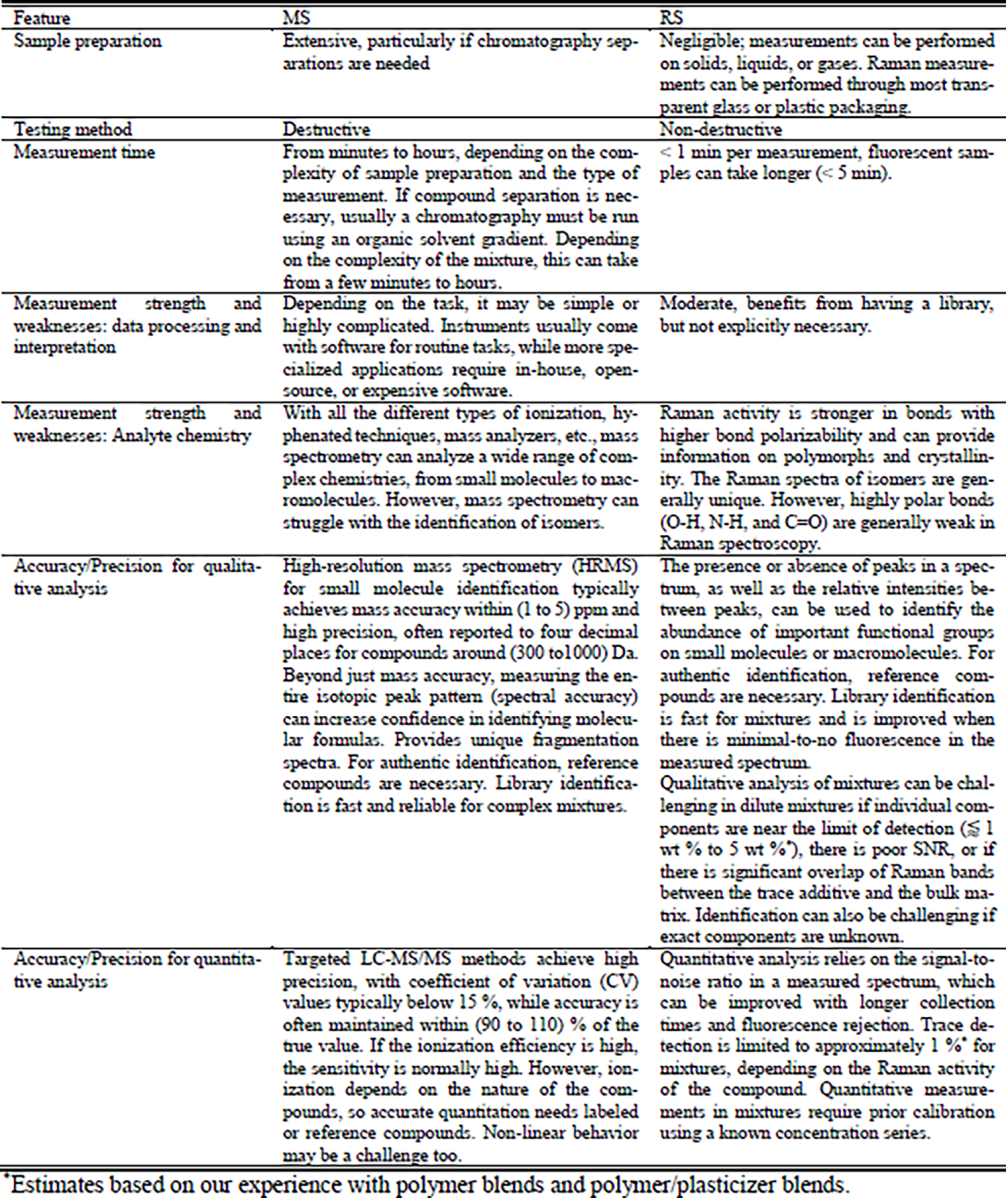
Comparison of RS and MS Methods Across Dimensions of Analytical and Measurement Properties for Polymer Additive Characterization

Both MS and TGRS are powerful analytical techniques capable of qualitative and quantitative analysis, provided they are applied under appropriate conditions, and adequate libraries and calibration standards are available. For example, MS techniques are less appropriate when samples do not readily ionize or distinction between structural isomers is required. RS techniques are less appropriate when samples have low Raman activity, strong fluorescence, are mixtures with several constituents, or require analysis of dilute constituents (≲1%).

The existing literature suggests RS could offer significant value as a process monitoring technique, leveraging its speed and adaptability for in-line monitoring, which could inform the utilization of higher-resolution, off-line techniques like MS.^5,40,41^ However, there may also be value in exploring the synergistic structural information contained in MS and RS spectra. In principle, both RS and MS provide information about the structural connectivity and bond strength of molecules: RS via the relative energy required to induce characteristic vibrational/electronic modes, and MS via the structure of fragmentation products generated under different ionization conditions.

## 5 Conclusions

We have discussed the combined use of time-gated Raman spectroscopy and mass spectrometry in the analysis of neat plasticizers as well as dilute plasticizer loadings in PVC. We present two parallel plasticizer libraries, for both TGRS and APCI–MS, demonstrating that both techniques can discretely identify neat plasticizers as shown by cosine similarity analysis. Both libraries are publicly available through the NIST Public Data Repository, and the APCI–MS data has been included in the NIST Mass Spectral Library (Standard Reference Database 1A), beginning with the NIST26 release.

Highlighting key strengths of the respective techniques, the MS analysis demonstrates highly unique spectral signatures for each plasticizer, strong sensitivity with respect to dilute formulations, and highly accurate qualitative and quantitative characterization when an adequate library is available. TGRS measurements in contrast, tend to have strong “family resemblance” across plasticizers with similar core functional groups, likely enabling the ability to rapidly identify the presence of specific categories of plasticizers in a polymer, provided the plasticizer loading is sufficiently above the limit of detection.

In analysis of NIST SRM 2860, Phthalates in PVC, we compare the measurement capabilities of TGRS and MS measurements. Using APCI–MS, we demonstrate effective qualitative and quantitative characterization within error values reported in the SRM certificate. Applying TGRS in combination with cosine similarity against the plasticizer library, we identified a strong likelihood that the SRM 10% Loading condition contained phthalates. Given the low concentration of plasticizers in the SRM (0.5%, 10%) approaching the expected limit of detection for TGRS methods (≲1%), the positive identification of phthalates is promising; especially considering that commercial PVCs often have plasticizer loadings upward of 50% by weight.

Finally, we consider the application of RS and MS as complementary techniques, comparing their appropriate use cases and relative strengths. As a comparatively rapid technique requiring minimal sample preparation, RS can more readily be introduced onto process-lines for monitoring, and enabling strategic, higher-resolution sampling as needed.

## Supplementary Material











## Footnotes

a Certain commercial equipment, instruments, or materials are identified in this paper in order to adequately specify experimental procedure. Such identification does not imply recommendation or endorsement by the National Institute of Standards and Technology, nor does it imply that the materials or equipment identified are necessarily the best available for the purpose.

## Notes

Supporting Information A: supplementary tables of acronyms, full sample list along with details on data collection conditions, full description of MS and Raman spectroscopy trends associated with core chemical functionality (PDF)
Supporting Information B–D: unprocessed time-gated Raman spectra for SRM 2860 samples (blank, level I, level II) (CSV)
Supporting Information E: processed time-gated Raman spectra for SRM 2860 samples (blank, level I, level II) (CSV)
Rows store Raman shift values (cm^–1^) (CSV)
Columns are the samples (CSV)

## ∥

J.M.R.: Department of Forestry, Michigan State University, East Lansing, Michigan, USA

## References

[1] Wiesinger H., Wang Z., Hellweg S. (2021). Deep Dive
into Plastic Monomers,
Additives, and Processing Aids. Environ. Sci.
Technol..

[2] Wypych, G. Typical Methods of Quality Control of Plasticizers. In Handbook of Plasticizers, 4th ed.; Wypych, G. , Ed.; ChemTec Publishing, 2023; pp 105–130.

[3] Wypych, G. Specialized analytical methods in plasticizer testing. In Handbook of Plasticizers, 4th ed.; Wypych, G. , Ed.; ChemTec Publishing, 2023; Vol. 30, pp 667–677.

[4] Beigzadeh K., Rieland J. M., Eastman C. B., Duffy D. J., Love B. J. (2022). Characterization
of Ingested Plastic Microparticles Extracted from Sea Turtle Post-Hatchlings
at Necropsy. Microplastics.

[5] Birch Q. T., Potter P. M., Pinto P. X., Dionysiou D. D., Al-Abed S. R. (2021). Isotope ratio mass spectrometry and
spectroscopic techniques
for microplastics characterization. Talanta.

[6] da Costa, J. P. ; Duarte, A. C. ; Rocha-Santos, T. A. P. Microplastics—Occurrence, Fate and Behaviour in the Environment. In Comprehensive Analytical Chemistry; Rocha-Santos, T. A. P. , Duarte, A. C. , Eds.; Elsevier, 2017; Vol. 75, pp 1–20.

[7] Gooneie A., Ragaert K. (2026). Additives: The Next
Frontier in Recycling Research. Engineering.

[8] Fremout W., Saverwyns S. (2012). Identification of synthetic organic
pigments:the role
of a comprehensive digital Raman spectral library. J. Raman Spectrosc..

[9] Nava V., Frezzotti M. L., Leoni B. (2021). Raman Spectroscopy for the Analysis
of Microplastics in Aquatic Systems. Appl. Spectrosc..

[10] Rieland, J. M. ; Kotula, A. P. ; Migler, K. B. ; Beers, K. L. Time-Gated Raman Plasticizer Library; NIST Public Data Repository, 2025.

[11] Čerkasova N., Enders K., Lenz R., Oberbeckmann S., Brandt J., Fischer D., Fischer F., Labrenz M., Schernewski G. (2023). A Public Database for Microplastics in the Environment. Microplastics.

[12] De
Frond H., Rubinovitz R., Rochman C. M. (2021). μATR-FTIR
Spectral Libraries of Plastic Particles (FLOPP and FLOPP-e) for the
Analysis of Microplastics. Anal. Chem..

[13] Dong, M. ; Zhang, Q. ; Luo, Z. A Raman Database of Microplastics Wweathered Under Natural Environments, 2 ed.; Mendeley Data, 2020.

[14] Miller E. A., Yamahara K. M., French C., Spingarn N., Birch J. M., Van Houtan K. S. (2022). A Raman spectral reference library of potential anthropogenic
and biological ocean polymers. Sci. Data.

[15] Munno K., De Frond H., O’Donnell B., Rochman C. M. (2020). Increasing the Accessibility
for Characterizing Microplastics: Introducing New Application-Based
and Spectral Libraries of Plastic Particles (SLoPP and SLoPP-E). Anal. Chem..

[16] da
Silva D. J., Wiebeck H. (2022). ATR-FTIR Spectroscopy Combined with
Chemometric Methods for the Classification of Polyethylene Residues
Containing Different Contaminants. J. Polym.
Environ..

[17] Li S., Ivancic R. J. S., Sutliff B. P., Huang D., Blázquez-Blázquez E., Martin T. B., Migler K. B., Audus D. J., Orski S. V. (2026). Predicting
Properties from Near-Infrared Spectra with Machine Learning for Improved
Polyolefin Differentiation. ACS Polym. Au.

[18] Neo E. R. K., Low J. S. C., Goodship V., Coles S. R., Debattista K. (2023). Development
of a Polymer Spectral Database for Advanced Chemometric Analysis. Proced. CIRP.

[19] Kögler M., Heilala B. (2020). Time-gated Raman spectroscopy
- a review. Meas. Sci. Technol..

[20] Kotula A. P., Orski S. V., Brignac K. C., Lynch J. M., Heilala B. M. J. (2022). Time-gated
Raman spectroscopy of recovered plastics. Mar.
Pollut. Bull..

[21] Neale A. R., Sazanovich I. V., Hardwick L. J. (2024). Gating-out emission for fluorescence-free
Raman spectra for the study of electrode interfaces. Curr. Opin. Electrochem..

[22] Guo S., Popp J., Bocklitz T. (2021). Chemometric
analysis in Raman spectroscopy
from experimental design to machine learning-based modeling. Nat. Protoc..

[23] McCreery, R. L. Magnitude of Raman Scattering. In Raman Spectroscopy for Chemical Analysis; Winefordner, J. D. , Ed.; Wiley-Interscience, 2000; Vol. 157, pp 15–34.

[24] Huang D. E., Kotula A. P., Snyder C. R., Migler K. B. (2022). Crystallization
Kinetics in an Immiscible Polyolefin Blend. Macromolecules.

[25] Gross, J. H. Mass Spectrometry: A Textbook, 3 ed.; Springer International Publishing, 2017.

[26] Rahman T., Meadows D., Zhan K., Senthil
Kumar H. K., Smith M., Davis V. A., Beckingham B. S., Peng Y., Davis E. (2026). Detection, identification, and quantification
of polymer additives: a review of techniques, approaches, challenges,
and a possible roadmap in analysis. RSC Appl.
Polym..

[27] Simón-Manso Y., Erisman E. P., Mak T. D., Burke M. C., Zuber A., Yang X., Liang Y., Neta P., Bukhari T., Williams A. J. (2025). NIST Mass Spectral Libraries in the Context
of the Circular Economy of Plastics. J. Am.
Soc. Mass Spectrom..

[28] Wallace W. E., Moorthy A. S. (2023). NIST Mass Spectrometry Data Center standard reference
libraries and software tools: Application to seized drug analysis. J. Forensic Sci..

[29] Wesdemiotis C., Williams-Pavlantos K. N., Keating A. R., McGee A. S., Bochenek C. (2024). Mass spectrometry of polymers: A tutorial review. Mass Spectrom. Rev..

[30] Wypych, G. Plasticizers use and selection for specific polymers. In Handbook of Plasticizers, 4th ed.; Wypych, G. , Ed.; ChemTec Publishing, 2023; pp 313–471.

[31] CPSC . Phthalates Business Guidance; United States Consumer Product Safety Commission, https://www.cpsc.gov/Business--Manufacturing/Business-Education/Business-Guidance/Phthalates (accessed Dec 15, 2025).

[32] ECHA . Phthalates. https://echa.europa.eu/hot-topics/phthalates (accessed Dec 15, 2025).

[33] USDA . Phthalates in Food Packaging and Food Contact Applications, 2024. https://www.fda.gov/food/food-additives-and-gras-ingredients-information-consumers/phthalates-food-packaging-and-food-contact-applications (accessed Dec 15, 2025).

[34] Wypych, G. Plasticizer types. In Handbook of Plasticizers, 4th ed.; Wypych, G. , Ed.; ChemTec Publishing, 2023; pp 9–104.

[35] ICCA Plastic Additives Database. Global Partners For Plastics Circularity, 2026. https://plasticscircularity.org/additives/ (accessed Mar 16, 2026).

[36] Immergut, E. H. ; Mark, H. F. Principles of Plasticization. In Principles of Plasticization; ACS, 1965; Vol. 48, pp 1–26.

[37] Bocqué M., Voirin C., Lapinte V., Caillol S., Robin J. J. (2016). Petro-based
and bio-based plasticizers: Chemical structures to plasticizing properties. J. Polym. Sci., Part A: Polym. Chem..

[38] Nørbygaard T., Berg R. W. (2004). Analysis of Phthalate Ester Content in Poly­(Vinyl Chloride)
Plastics by Means of Fourier Transform Raman Spectroscopy. Appl. Spectrosc..

[39] Saeki K., Funatsu K., Tanabe K. (2003). Discrimination of Poly­(vinyl
chloride)
Samples with Different Plasticizers and Prediction of Plasticizer
Contents in Poly­(vinyl chloride) Using Near-infrared Spectroscopy
and Neural-network Analysis. Anal. Sci..

[40] Bocklitz T. W., Crecelius A. C., Matthäus C., Tarcea N., von Eggeling F., Schmitt M., Schubert U. S., Popp J. (2013). Deeper Understanding
of Biological Tissue: Quantitative Correlation of MALDI-TOF and Raman
Imaging. Anal. Chem..

[41] Das N. K., Dai Y., Liu P., Hu C., Tong L., Chen X., Smith Z. J. (2017). Raman Plus X: Biomedical Applications of Multimodal
Raman Spectroscopy. Sensors.

[42] Klisińska-Kopacz A., Łydżba-Kopczyńska B., Czarnecka M., Koźlecki T., del Hoyo Mélendez J., Mendys A., Kłosowska-Klechowska A., Obarzanowski M., Frączek P. (2019). Raman spectroscopy as a powerful technique for the
identification of polymers used in cast sculptures from museum collections. J. Raman Spectrosc..

[43] Heudt L., Debois D., Zimmerman T. A., Köhler L., Bano F., Partouche F., Duwez A.-S., Gilbert B., De Pauw E. (2012). Raman spectroscopy and laser desorption mass spectrometry
for minimal destructive forensic analysis of black and color inkjet
printed documents. Forensic Sci. Int..

[44] Ghebremedhin M., Heitkamp R., Yesupriya S., Clay B., Crane N. J. (2017). Accurate
and Rapid Differentiation of Acinetobacter baumannii Strains by Raman
Spectroscopy: a Comparative Study. J. Clin.
Microbiol..

[45] Ríos-Reina R., Salatti-Dorado J. Á., Ortiz-Romero C., Cardador M. J., Arce L., Callejón R. (2024). A comparative
study of fluorescence and Raman spectroscopy for discrimination of
virgin olive oil categories: Chemometric approaches and evaluation
against other techniques. Food Control.

[46] McCreery, R. L. Calibration and validation. In Raman Spectroscopy for Chemical Analysis; Winefordner, J. D. , Ed., 2000; Vol. 157, pp 251–291.

[47] Rieland, J. M. TGRS-Plasticizer-Library, 2025. https://github.com/usnistgov/TGRS-Plasticizer-Library/tree/main (accessed Jan 20, 2026).

[48] Simón-Manso, Y. ; Zuber, A. ; Rieland, J. M. ; Wallace, W. E. Plasticizer Tandem Mass Spectral Library: APCI-timsTOF Measurements; NIST Public Data Repository, 2026.

[49] Berthelette, K. ; Walter, T. H. Column Screening for the UPLC Separation of Plastic Additives as Part of Extractables and Leachables Workflows; Waters Corporation: MA, USA, 2020.

[50] Lee, P. J. ; Di Gioia, A. J. Rapid Analysis of 25 Common Polymer Additives; Waters Corporation: Milford, MA, USA, 2008.

[51] Lam H., Deutsch E. W., Eddes J. S., Eng J. K., Stein S. E., Aebersold R. (2008). Building consensus
spectral libraries for peptide identification
in proteomics. Nat. Methods.

[52] Cooper B. T., Yan X., Simón-Manso Y., Tchekhovskoi D. V., Mirokhin Y. A., Stein S. E. (2019). Hybrid Search: A Method for Identifying
Metabolites Absent from Tandem Mass Spectrometry Libraries. Anal. Chem..

[53] Socrates, G. Infrared and Raman Characteristic Group Frequencies; John Wiley & Sons, LTD, 2001.

[54] Daniels P. H. (2005). Hydrocarbon
Secondary Plasticizers as Functional Additives in Flexible PVC Compounds. J. Vinyl Addit. Technol..

[55] Chen W., Liu J., Hou X., Jiang G. (2023). A review on biological occurrence,
bioaccumulation, transmission and metabolism of chlorinated paraffins. Crit. Rev. Environ. Sci. Technol..

[56] EPA . Risk Management for short-chain Chlorinated Paraffins; United States Environmental Protection Agency, https://www.epa.gov/assessing-and-managing-chemicals-under-tsca/risk-management-short-chain-chlorinated-paraffins (accessed Dec 15, 2025).

[57] Guida Y., Capella R., Weber R. (2020). Chlorinated
paraffins in the technosphere:
A review of available information and data gaps demonstrating the
need to support the Stockholm Convention implementation. Emerging Contam..

[58] Chao H.-C., Lee K. W., Shih M., McLuckey S. A. (2022). Characterization
of Homopolymer Distributions via Direct Infusion ESI-MS/MS using Wide
Mass-to-Charge Windows and Gas-Phase Ion/Ion Reactions. J. Am. Soc. Mass Spectrom..

[59] Scionti V., Wesdemiotis C. (2012). Electron transfer dissociation versus collisionally
activated dissociation of cationized biodegradable polyesters. J. Mass Spectrom..

[60] Mostafapour S., Mokari A., Guo S., Popp J., Bocklitz T. (2026). Raman spectra
comparison: cautions and pitfalls of similarity metrics. Spectrochim. Acta, Part A.

[61] Stein S. E., Scott D. R. (1994). Optimization and
testing of mass spectral library search
algorithms for compound identification. J. Am.
Soc. Mass Spectrom..

[62] SRM 2860 Certificate of Analysis: Phthalates in Polyvinyl Chloride; National Institute of Standards and Technology, 2018.

[63] Meher A. K., Chen Y.-C. (2016). Combination of Raman
Spectroscopy and Mass Spectrometry
for Online Chemical Analysis. Anal. Chem..

