# Supplementary material for: A Time-Gated Raman Spectroscopy and Mass Spectrometry Comparative Chemical Analysis of Plasticizers
Source: ACS Polym Au. 2026 Jul 13;6(4):1134–46. doi: 10.1021/acspolymersau.6c00065 (PMC13474348; doi:10.1021/acspolymersau.6c00065)
Supplement: Supplementary file 1 [file lg6c00065_si_001.pdf]

Supplementary Information for:

A time-gated Raman spectroscopy and mass spectrometry comparative chemical analysis of plasticizers

Julie M. Rieland[a*], Yamil Simón-Manso[b*], Adam Zuber[b], Anthony P. Kotula[a], Kalman B. Migler[a], William E. Wallace[b], Kathryn L. Beers[c]

[a]Materials Science and Engineering Division, National Institute of Standards and Technology, Gaithersburg, MD 20899, United States of America

[b]Mass Spectrometry Data Center, Biomolecular Measurement Division, National Institute of Standards and Technology (NIST), Gaithersburg, Maryland, 20899, United States of America

[c]Materials Measurement Lab, National Institute of Standards and Technology, Gaithersburg, MD 20899, United States of America

Table of Contents:

I. List of acronyms S2

II. List of plasticizers S3

III. Plasticizer molecular structures S7

IV. Time-gated Raman library measurement conditions and data quality S9

V. Time-gated Raman data processing S15

VI. LC-MS library preparation....................S17

VII. Instructions for using NIST Mass Spectral Library tools S18

VIII. Mass spectrometry library measurement conditions S20

IX. Mass spectrometry techniques for polymer and plasticizers analysis S47

X. Raman and MS spectral signatures associated with core functional groups S48

XI. Raman peak assignments by core functional family....................S55

XII. Time-gate Raman analysis of SRM 2860 ....S59

XIII. APCI-LC-MS/MS analysis of SRM 2860 ....S61

XIV. SI References S65

I. List of acronyms

Table S1. List of acronyms

Acronym	Meaning
APCI	atmospheric pressure chemical ionization
BBP	benzyl butyl phthalate
CAS	chemical abstracts service
CS	cosine similarity
DBP	di-n-butyl phthalate
DEHA	di (2-ethylhexyl) adipate and bis (2-ethylhexyl) adipate, see also DOA
DEHP	di (2-ethylhexyl) phthalate, industrially referred to as DOP (dioctyl phthalate), which is distinct from "di-n-octyl phthalate" (DNOP). Also bis (2-ethylhexyl) phthalate
DIDP	diisodecyl phthalate
DINP	diisononyl phthalate
DNOP	di-n-octyl phthalate
DOA	bis (2-ethylhexyl) adipate or di (2-ethylhexyl) adipate
FT-ICR	Fourier transform ion cyclotron resonance
FTIR	Fourier transform infrared spectroscopy
GC-MS	gas chromatography mass spectrometry
IR	Infrared (spectroscopy)
IUPAC	International Union of Pure and Applied Chemistry
LC-MS	liquid chromatography mass spectrometry
m/z	mass to charge ratio
MS	mass spectrometry
NIR	near infrared spectroscopy
NIST	National Institute of Standards and Technology
PCA	principal component analysis
PE	polyethylene
PP	polyethylene
PS	polystyrene
SBR	signal to background ratio
SNR	signal to noise ratio
SRM	standard reference material
TCPh	tricresyl phosphate
TGRS	time-gated raman spectroscopy
TOF	time-of-flight

II. List of plasticizers included in this study

Table S2. List of plasticizers

Plasticizer	Acronym	IUPAC Name	Cas #	SMILES
2,2,4-Trimethyl-1,3-pentanediol diisobutyrate	TPDB	[2,2,4-trimethyl-3-(2-methylpropanoyloxy)pentyl] 2-methylpropanoate	6846-50-0	CC(C)C(C(C)(C)COC(=O)C(C)C)OC(=O)C(C)C
2-Ethylhexyl diphenyl phosphate	EHDPh	2-ethylhexyl diphenyl phosphate	1241-94-7	CCCCC(CC)COP(=O)(OC1=CC=CC=C1)OC2=CC=CC=C2
2-Ethylhexyl epoxy tallate	EHET	(3,4,5-trihydroxybenzoyl)oxy 2-(2-ethylhexyl)-3,4,5-trihydroxybenzenecarboperoxoate	61789-01-3	CCCCC(CC)CC1=C(C(=C(C=C1C(=O)OOOC(=O)C2=CC(=C(C(=C2)O)O)O)O)O)O
Acetyl triethyl citrate	ATEC	Triethyl 2-acetyloxypropane-1,2,3-tricarboxylate	77-89-4	CCOC(=O)CC(CC(=O)OCC)(C(=O)OCC)OC(=O)C
Acetyl tri-n-butyl citrate	ATBC	Tributyl 2-acetyloxypropane-1,2,3-tricarboxylate	77-90-7	CCCCOC(=O)CC(CC(=O)OCCCC)(C(=O)OCCCC)OC(=O)C
Bis(2-hydroxyethyl) dimerate	DHOED	Bis(2-hydroxyethyl) dimerate	*68855-78-7*	-
Butyl benzyl phthalate	BBP	2-*O*-benzyl 1-*O*-butyl benzene-1,2-dicarboxylate	85-68-7	CCCCOC(=O)C1=CC=CC=C1C(=O)OCC2=CC=CC=C2
Butyl octyl phthalate	BOP	1-*O*-butyl 2-*O*-octyl benzene-1,2-dicarboxylate	84-78-6	CCCCCCCCOC(=O)C1=CC=CC=C1C(=O)OCCCC
Butyl oleate	BO	Butyl (*Z*)-octadec-9-enoate	142-77-8	CCCCCCCC/C=C\CCCCCCCC(=O)OCCCC
Butyl ricinoleate	BRo	Butyl (*Z*,12*R*)-12-hydroxyoctadec-9-enoate	151-13-3	CCCCCC[C@H](C/C=C\CCCCCCCC(=O)OCCCC)O
Chloroparaffin 41 % Cl	CP-41Cl	4,8,11,14,17,21-hexachlorotetracosane	*63449-39-8*	CCCC(CCCC(CCC(CCC(CCC(CCCC(CCC)Cl)Cl)Cl)Cl)Cl)Cl
Chloroparaffin 50 % Cl	CP-50Cl	4,8,11,14,17,21-hexachlorotetracosane	*63449-39-8*	CCCC(CCCC(CCC(CCC(CCC(CCCC(CCC)Cl)Cl)Cl)Cl)Cl)Cl
Chloroparaffin 60 % Cl	CP-60Cl	2,3,4,6,7,8-hexachlorodecane	108171-26-2	CCCC(CCCC(CCC(CCC(CCC(CCCC(CCC)Cl)Cl)Cl)Cl)Cl)Cl
Chloroparaffin 70 % Cl	CP-70Cl	4,8,11,14,17,21-hexachlorotetracosane	*63449-39-8*	CCCC(CCCC(CCC(CCC(CCC(CCCC(CCC)Cl)Cl)Cl)Cl)Cl)Cl
Di(2-ethylhexyl) adipate	DEHA	Bis(2-ethylhexyl) adipate	103-23-1	CCCCC(CC)COC(=O)CCCCC(=O)OCC(CC)CCCC
Di(2-ethylhexyl) azelate	DEHAz	Bis(2-ethylhexyl) nonanedioate	103-24-2	-
Di(2-ethylhexyl) isophthalate	DEHIP	Bis(2-ethylhexyl) benzene-1,3-dicarboxylate	137-89-3	CCCCC(CC)COC(=O)C1=CC(=CC=C1)C(=O)OCC(CC)CCCC
Di(2-ethylhexyl) maleate	DEHM	Bis(2-ethylhexyl) (*Z*)-but-2-enedioate	142-16-5	CCCCC(CC)COC(=O)/C=C\C(=O)OCC(CC)CCCC
Di(2-ethylhexyl) phthalate	DEHP	Bis(2-ethylhexyl) benzene-1,2-dicarboxylate	117-81-7	CCCCC(CC)COC(=O)C1=CC=CC=C1C(=O)OCC(CC)CCCC
Di(2-ethylhexyl) sebacate	DEHS	Bis(2-ethylhexyl) decanedioate	122-62-3	CCCCC(CC)COC(=O)CCCCCCCCC(=O)OCC(CC)CCCC
Di(n-heptyl, n-nonyl) adipate	DHNA	Di(n-heptyl, n-nonyl) adipate	68515-75-3	CCCCCCCCCOC(CCCCC(OCCCCCCC)=O)=O

Di(tridecyl) adipate	DTDA	Ditridecyl hexanedioate	16958-92-2	CCCCCCCCCCCCCOC(=O)CCCCC(=O)OCCCCCCCCCCCCC
Dibutyl fumarate	DBF	Dibutyl (*E*)-but-2-enedioate	105-75-9	CCCCOC(=O)/C=C/C(=O)OCCCC
Dibutyl sebacate	DBS	Dibutyl decanedioate	109-43-3	CCCCOC(=O)CCCCCCCCC(=O)OCCCC
Dicapryl adipate	DCA	Didecyl hexanedioate	105-97-5	CCCCCCCCCCOC(=O)CCCCC(=O)OCCCCCCCCCC
Dicapryl phthalate	DCP	Dioctan-2-yl benzene-1,2-dicarboxylate	131-15-7	C1(C(OC(C)CCCCCC)=O)=CC=CC=C1C(OC(C)CCCCCC)=O
Dicyclohexyl phthalate	DCHP	Dicyclohexyl benzene-1,2-dicarboxylate	84-61-7	C1CCC(CC1)OC(=O)C2=CC=CC=C2C(=O)OC3CCCCC3
Diethyl phthalate	DEP	Diethyl benzene-1,2-dicarboxylate	84-66-2	CCOC(=O)C1=CC=CC=C1C(=O)OCC
Diethyl succinate	DES	Diethyl butanedioate	123-25-1	CCOC(=O)CCC(=O)OCC
Diethylene glycol dibenzoate	DEGD	2-(2-benzoyloxyethoxy)ethyl benzoate	120-55-8	C1=CC=C(C=C1)C(=O)OCCOCCOC(=O)C2=CC=CC=C2
Dihexyl phthalate	DHP	Dihexyl benzene-1,2-dicarboxylate	84-75-3	CCCCCCOC(=O)C1=CC=CC=C1C(=O)OCCCCCC
Diisobutyl adipate	DIBA	Bis(2-methylpropyl) hexanedioate	141-04-8	CC(C)COC(=O)CCCCC(=O)OCC(C)C
Diisobutyl phthalate	DIBP	Bis(2-methylpropyl) benzene-1,2-dicarboxylate	84-69-5	CC(C)COC(=O)C1=CC=CC=C1C(=O)OCC(C)C
Diisodecyl adipate	DIDA	Bis(8-methylnonyl) hexanedioate	27178-16-1	CC(C)CCCCCCCOC(=O)CCCCC(=O)OCCCCCCCC(C)C
Diisodecyl azelate	DIDAz	Bis(8-methylnonyl) nonanedioate	28472-97-1	CC(C)CCCCCCCOC(=O)CCCCCCCC(=O)OCCCCCCCC(C)C
Diisodecyl phthalate	DIDP	Bis(8-methylnonyl) benzene-1,2-dicarboxylate	26761-40-0	CC(C)CCCCCCCOC(=O)C1=CC=CC=C1C(=O)OCCCCCCCC(C)C
Diisoheptyl phthalate	DIHP	Bis(5-methylhexyl) benzene-1,2-dicarboxylate	41451-28-9	CC(C)CCCCOC(=O)C1=CC=CC=C1C(=O)OCCCCC(C)C
Diisononyl adipate	DINA	Bis(7-methyloctyl) hexanedioate	33703-08-1	CC(C)CCCCCCOC(=O)CCCCC(=O)OCCCCCCC(C)C
Diisononyl phthalate	DINP	Bis(7-methyloctyl) benzene-1,2-dicarboxylate	28553-12-0	CC(C)CCCCCCOC(=O)C1=CC=CC=C1C(=O)OCCCCCCC(C)C
Diisooctyl azelate	DIOAz	Bis(6-methylheptyl) nonanedioate	106-03-6	CC(C)CCCCCOC(=O)CCCCCCCC(=O)OCCCCCC(C)C
Diisooctyl phthalate	DIOP	Bis(6-methylheptyl) benzene-1,2-dicarboxylate	27554-26-3	CC(C)CCCCCOC(=O)C1=CC=CC=C1C(=O)OCCCCCC(C)C
Dimethyl azelate	DMAz	Dimethyl nonanedioate	1732-10-1	COC(=O)CCCCCCCC(=O)OC
Dimethyl isophthalate	DMIP	Dimethyl benzene-1,3-dicarboxylate		
Dimethyl phthalate	DMP	Dimethyl benzene-1,2-dicarboxylate		

1459-93-4 COC(=O)C1=CC(=CC=C1)C(=O)OC

			131-11-3	COC(=O)C1=CC=CC=C1C(=O)OC
Dimethyl sebacate	DMS	Dimethyl decanedioate	106-79-6	COC(=O)CCCCCCCCC(=O)OC
Di-n-butyl maleate	DBM	Dibutyl (*Z*)-but-2-enedioate	105-76-0	CCCCOC(=O)/C=C\C(=O)OCCCC
Di-n-butyl phthalate	DBP	Dibutyl benzene-1,2-dicarboxylate	84-74-2	CCCCOC(=O)C1=CC=CC=C1C(=O)OCCCC
Di-n-hexyl azelate	DHAz	Dihexyl nonanedioate	109-31-9	CCCCCCOC(=O)CCCCCCCC(=O)OCCCCCC

Diphenyl isophthalate	DPIP	Diphenyl benzene-1,3-dicarboxylate	744-45-6	C1=CC=C(C=C1)OC(=O)C2=CC(=CC=C2)C(=O)OC3=CC=CC=C3
Dipropylene glycol dibenzoate	DPGD	2-(1-benzoyloxypropan-2-yloxy)propyl benzoate	27138-31-4	CC(COC(=O)C1=CC=CC=C1)OC(C)COC(=O)C2=CC=CC=C2
Ditridecyl phthalate	DTDP	Ditridecyl benzene-1,2-dicarboxylate	119-06-2	CCCCCCCCCCCCCOC(=O)C1=CC=CC=C1C(=O)OCCCCCCCCCCCCC
Diundecyl phthalate	DUP	Diundecyl benzene-1,2-dicarboxylate	3648-20-2	CCCCCCCCCCCOC(=O)C1=CC=CC=C1C(=O)OCCCCCCCCCCC
Epoxidized linseed oil	ELO	-	8016-11-3	-
Epoxidized soybean oil	ESBO	-	8013-07-8	-
Ethylene glycol monostearate	EGMSt	2-hydroxyethyl octadecanoate	111-60-4	CCCCCCCCCCCCCCCCCC(=O)OCCO
Glycerol monooleate	GMo	2,3-dihydroxypropyl (*Z*)-octadec-9-enoate	111-03-5	CCCCCCCC/C=C\CCCCCCCC(=O)OCC(CO)O
Glycerol monostearate	GMSt	2,3-dihydroxypropyl octadecanoate	123-94-4	CCCCCCCCCCCCCCCCCC(=O)OCC(CO)O
Glycerol trioleate	GTo	2,3-bis[[(*Z*)-octadec-9-enoyl]oxy]propyl (*Z*)-octadec-9-enoate	122-32-7	CCCCCCCC/C=C\CCCCCCCC(=O)OCC(OC(=O)CCCCCCC/C=C\CCCCCCCC)COC(=O)CCCCCCC/C=C\CCCCCCCC
Glyceryl tri-(acetyl ricinoleate)	GTARo	2,3-bis[[(*Z*)-12-acetyloxyoctadec-9-enoyl]oxy]propyl (*Z*)-12-acetyloxyoctadec-9-enoate	*101-34-8*	CCCCCCC(OC(=O)C)C/C=C\CCCCCCCC(=O)OCC(OC(=O)CCCCCCC/C=C\CC(OC(=O)C)CCCCCC)COC(=O)CCCCCCC/C=C\CC(OC(=O)C)CCCCCC
Glyceryl triacetate	GTA	2,3-diacetyloxypropyl acetate	102-76-1	CC(=O)OCC(COC(=O)C)OC(=O)C
Isodecyl diphenyl phosphate	IDDPh	8-methylnonyl diphenyl phosphate	29761-21-5	CC(C)CCCCCCCOP(=O)(OC1=CC=CC=C1)OC2=CC=CC=C2
Isopropyl isostearate	IPISt	Propan-2-yl 16-methylheptadecanoate	31478-84-9	CC(C)CCCCCCCCCCCCCCC(=O)OC(C)C
Isopropyl myristate	IPM	Propan-2-yl tetradecanoate	110-27-0	CCCCCCCCCCCCCC(=O)OC(C)C
Isopropyl palmitate	IPP	Propan-2-yl hexadecanoate	142-91-6	CCCCCCCCCCCCCCCC(=O)OC(C)C
Methyl acetyl ricinoleate	MARo	Methyl (*Z*,12*R*)-12-acetyloxyoctadec-9-enoate	140-03-4	CCCCCC[C@H](C/C=C\CCCCCCCC(=O)OC)OC(=O)C
Methyl laurate	ML	Methyl dodecanoate	111-82-0	CCCCCCCCCCCC(=O)OC
Methyl linoleate	MLo	Methyl (9*Z*,12*Z*)-octadeca-9,12-dienoate	112-63-0	CCCCC/C=C\C/C=C\CCCCCCCC(=O)OC
Methyl oleate	MO	Methyl (*Z*)-octadec-9-enoate	112-62-9	CCCCCCCC/C=C\CCCCCCCC(=O)OC

Methyl palmitate	MP	Methyl hexadecanoate	112-39-0	CCCCCCCCCCCCCCCC(=O)OC
Methyl ricinoleate	MRo	Methyl (*Z*,12*R*)-12-hydroxyoctadec-9-enoate	141-24-2	CCCCCC[C@H](C/C=C\CCCCCCCC(=O)OC)O
Methyl stearate	MSt	Methyl octadecanoate	112-61-8	CCCCCCCCCCCCCCCCCC(=O)OC
N-Butyl acetyl ricinoleate	BARo	Butyl (*Z*,12*R*)-12-acetyloxyoctadec-9-enoate	140-04-5	[C@H](C/C=C\CCCCCCCC(OCCCC)=O)(CCCCCC)OC(C)=O
n-Butyl stearate	BSt	Butyl octadecanoate	123-95-5	CCCCCCCCCCCCCCCCCC(=O)OCCCC

N-Ethyl o,p-toluenesulfonamide	EOPTSN	*N*-ethyl-2-methylbenzenesulfonamide	8047-99-2	C1(C=CC=CC=1C)S(=O)(=O)NCC
N-Propyl oleate	PO	Propyl (*Z*)-octadec-9-enoate	111-59-1	CCCCCCCC/C=C\CCCCCCCC(=O)OCCC
o,p-Toluenesulfonamide	OPTSN	2-methylbenzenesulfonamide	88-19-7	CC1=CC=C(C=C1)S(=O)(=O)N.CC1=CC=CC=C1S(=O)(=O)N
Polyethylene glycol 200 dibenzoate	PEGD	2-benzoyloxyethyl benzoate	9004-86-8	-
Propylene glycol monostearate	PGMSt	2-hydroxypropyl octadecanoate	142-75-6	CCCCCCCCCCCCCCCCCC(=O)OCC(C)O
Propylene glycol ricinoleate	PGRo	2-hydroxypropyl (*Z*,12*R*)-12-hydroxyoctadec-9-enoate	71672-81-6	CCCCCC[C@H](C/C=C\CCCCCCCC(=O)OCC(C)O)O
T-Butylphenyl diphenyl phosphate	BPPPh	(2-*tert*-butylphenyl) diphenyl phosphate	56803-37-3	CC(C)(C)C1=CC=CC=C1OP(=O)(OC2=CC=CC=C2)OC3=CC=CC=C3
Tetrahydrofurfuryl oleate	THFO	Oxolan-2-ylmethyl (*Z*)-octadec-9-enoate	5420-17-7	CCCCCCCC/C=C\CCCCCCCC(=O)OCC1CCCO1
Tri(n-octyl, n-decyl) trimellitate	TODT	Decyl octyl benzene-1,2,4-tricarboxylate	*67989-23-5*	-
Tri-butoxyethyl phosphate	TBOEPh	Tris(2-butoxyethyl) phosphate	78-51-3	CCCCOCCOP(=O)(OCCOCCCC)OCCOCCCC
Tributyl phosphate	TBPh	Tributyl phosphate	126-73-8	CCCCOP(=O)(OCCCC)OCCCC
Tricapryl trimellitate	TCT	Tris(6-methylheptyl) benzene-1,2,4-tricarboxylate	27251-75-8	CC(C)CCCCCOC(=O)C1=CC(=C(C=C1)C(=O)OCCCCCC(C)C)C(=O)OCCCCCC(C)C
Tricresyl phosphate	TCPh	Tris(4-methylphenyl) phosphate	78-32-0	CC1=CC=C(C=C1)OP(=O)(OC2=CC=C(C=C2)C)OC3=CC=C(C=C3)C
Triethyl citrate	TEC	Triethyl 2-hydroxypropane-1,2,3-tricarboxylate	77-93-0	CCOC(=O)CC(CC(=O)OCC)(C(=O)OCC)O
Triisodecyl trimellitate	TIDT	Tris(8-methylnonyl) benzene-1,2,4-tricarboxylate	36631-30-8	CC(C)CCCCCCCOC(=O)C1=CC(=C(C=C1)C(=O)OCCCCCCCC(C)C)C(=O)OCCCCCCCC(C)C
Triisononyl trimellitate	TINT	Tris(7-methyloctyl) benzene-1,2,4-tricarboxylate	53894-23-8	CC(C)CCCCCCOC(=O)C1=CC(=C(C=C1)C(=O)OCCCCCCC(C)C)C(=O)OCCCCCCC(C)C
Tri-n-butyl citrate	TBC	Tributyl 2-hydroxypropane-1,2,3-tricarboxylate	77-94-1	CCCCOC(=O)CC(CC(=O)OCCCC)(C(=O)OCCCC)O
Triphenyl phosphate	TPPh	Triphenyl phosphate	115-86-6	C1=CC=C(C=C1)OP(=O)(OC2=CC=CC=C2)OC3=CC=CC=C3

III. Plasticizer molecular structures

Table S3. Generic plasticizer structures

**Aliphatic Esters**		
Oleates	Monoalkyl esters	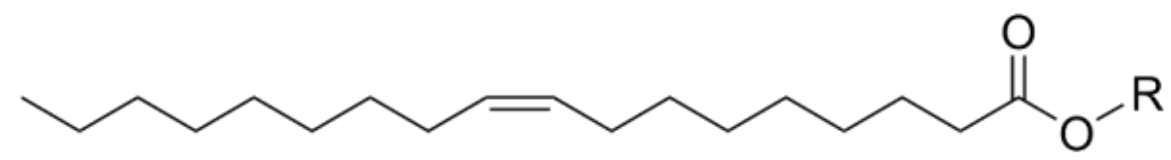
Ricinoleates	Monoalkyl esters	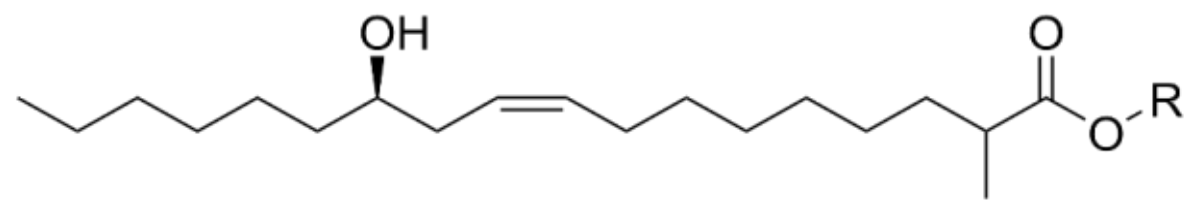
Stearates	Monoalkyl esters	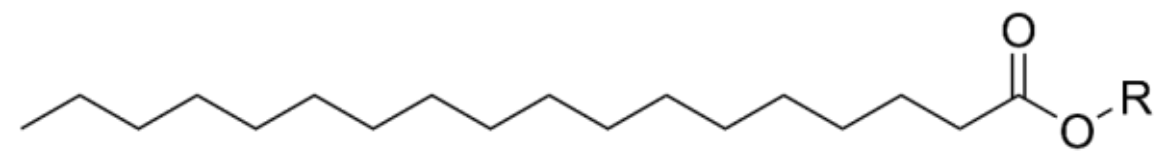
Plamitates	Monoalkyl esters	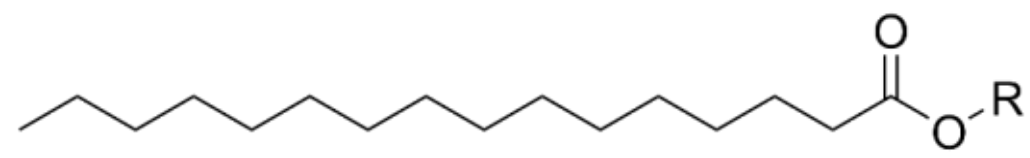
Adipates	Dialkyl esters	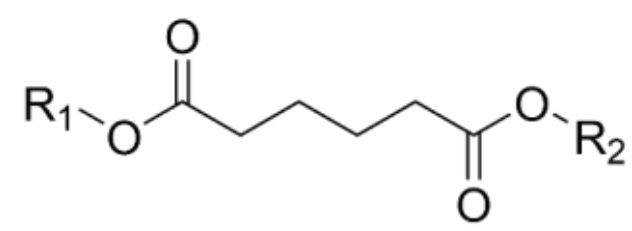
Azelates	Dialkyl esters	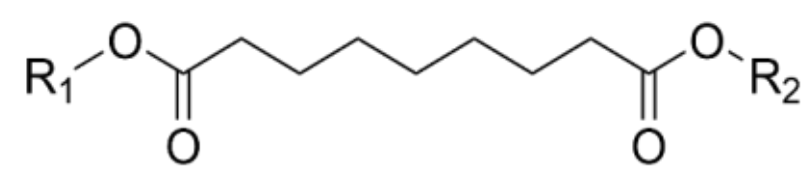
Sebacates	Dialkyl esters	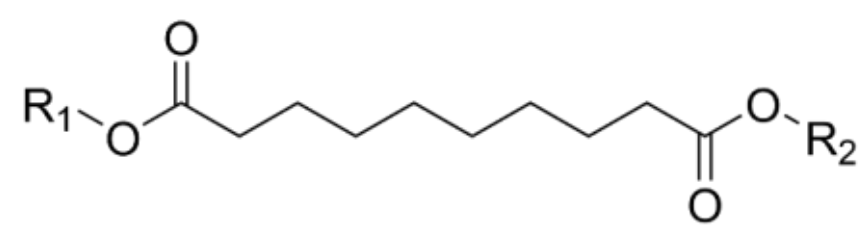
Maleates		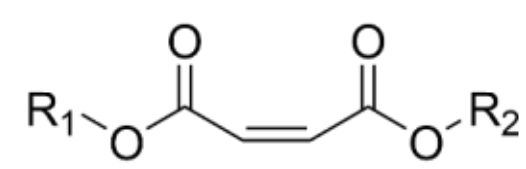
Citrates	Trialkyl esters	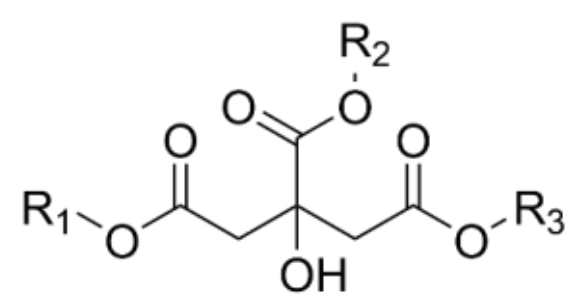
Glycol and glycerol esters	Mono/Di/Tri esters	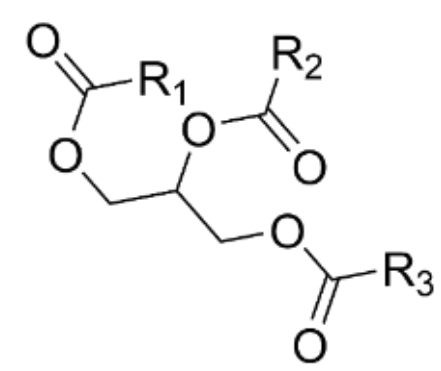
**Aromatic Esters**		
Phthalates		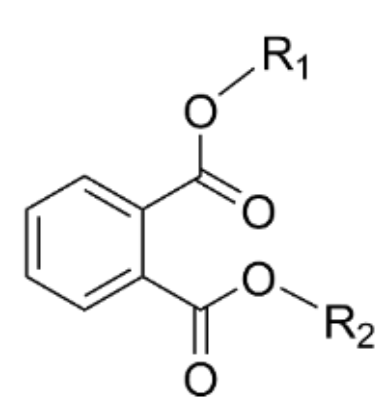

Isophthalates	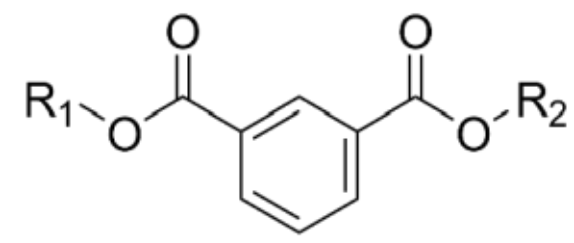
Trimellitates	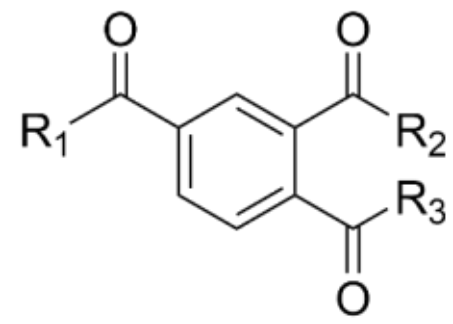
Dibenzoates	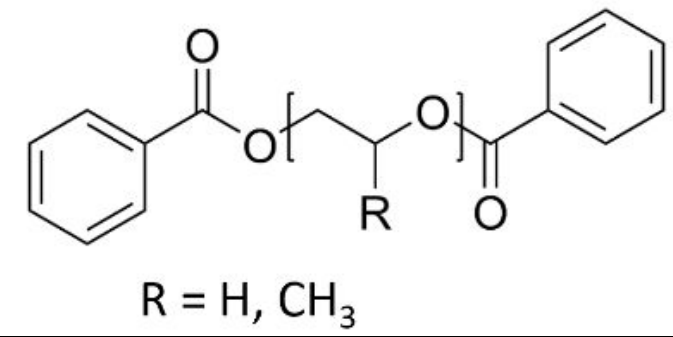
**Non-Esters**	
Organophosphates	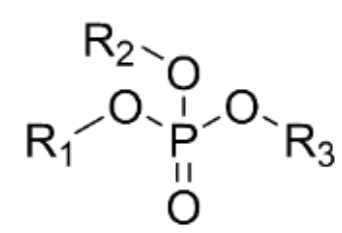
Aromatic sulfonamides	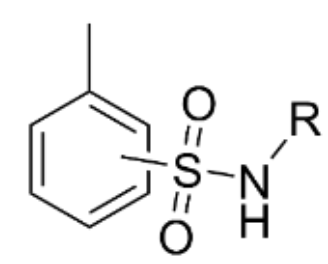
**Oligomeric Plasticizers**	
Chloroparaffins	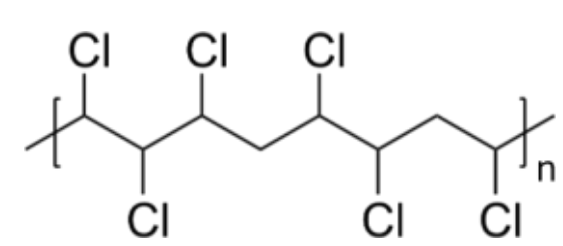
Epoxidized Oils	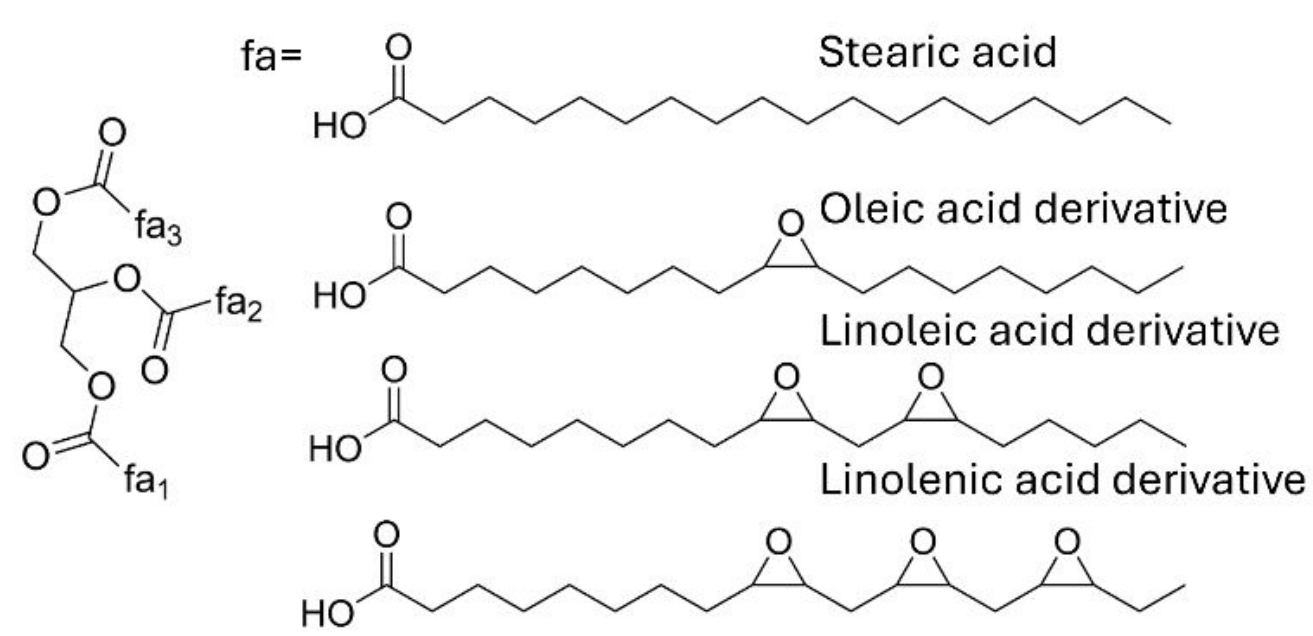

IV. Time-gated Raman measurement conditions and data quality

Table S4. List of time-gated Raman samples with measurement conditions*, Signal-to-Noise ratio (SNR), and Signal-to-Background ratio (SBR)*

**Sample Name**[a]	**Time Gating (ns)**	**Calibration Shift (cm^{-1})**	**Laser Power (mW)**	**Sub Acquisitions -**	**SNR -**	**SBR -**
2,2,4-trimethyl-1,3-pentanediol diisobutyrate_10	5.5-5.8	-0.158	10	300	1105	1073
2,2,4-trimethyl-1,3-pentanediol diisobutyrate_99	5.5-5.8	-0.158	10	300	1105	989
2-ethylhexyl diphenyl phosphate_02	5.5-5.8	-0.343	10	300	1450	1684
2-ethylhexyl diphenyl phosphate_03	5.5-5.8	-0.343	10	300	1450	1556
2-ethylhexyl epoxy tallate_64	5.5-5.8	-0.158	10	300	14	290
2-ethylhexyl epoxy tallate_68	5.5-5.8	-0.158	10	300	14	179
acetyl triethyl citrate_73	5.5-5.8	-0.158	10	300	187	351
acetyl triethyl citrate_74	5.5-5.8	-0.158	10	300	187	359
acetyl tri-n-butyl citrate_18	5.5-5.8	-2.655	10	100	63	186
acetyl tri-n-butyl citrate_19	5.5-5.8	-2.655	10	100	63	185
acetyl tri-n-butyl citrate_71	5.5-5.8	-0.158	10	300	63	312
acetyl tri-n-butyl citrate_72	5.5-5.8	-0.158	10	300	63	294
bis(2-hydroxyethyl) dimerate_10	5.5-5.8	-0.158	15	300	113	154
bis(2-hydroxyethyl) dimerate_98	5.5-5.8	-0.158	15	300	113	137
bis(2-hydroxyethyl) dimerate_99	5.5-5.8	-0.158	15	300	113	159
butyl acetyl ricinoleate_07	5.5-5.8	-0.158	10	300	18	96
butyl acetyl ricinoleate_09	5.5-5.8	-0.158	10	300	18	83
butyl oleate_87	5.5-5.8	-0.158	15	300	188	182
butyl oleate_88	5.5-5.8	-0.158	15	300	188	203
butyl ricinoleate_76	5.5-5.8	-0.158	15	300	68	109
butyl ricinoleate_77	5.5-5.8	-0.158	15	300	68	136
butylbenzyl phthalate_26	5.5-5.8	-2.655	10	100	103	168
butylbenzyl phthalate_27	5.5-5.8	-2.655	10	100	103	178
butyloctyl phthalate_28	5.5-5.8	-2.655	10	100	244	268
butyloctyl phthalate_29	5.5-5.8	-2.655	10	100	244	283
chloroparaffin 41p Cl_91	5.5-5.8	-0.158	10	100	189	294
chloroparaffin 41p Cl_92	5.5-5.8	-0.158	10	300	189	286
chloroparaffin 50p Cl_93	5.5-5.8	-0.158	10	300	305	434
chloroparaffin 50p Cl_94	5.5-5.8	-0.158	10	300	305	441

chloroparaffin 60p Cl_95	5.5-5.8	-0.158	10	300	172	252
chloroparaffin 60p Cl_96	5.5-5.8	-0.158	10	300	172	269
chloroparaffin 70p Cl_80	5.5-5.8	-0.158	15	300	51	42
chloroparaffin 70p Cl_81	5.5-5.8	-0.158	15	300	51	38
di(2-ethylhexyl) adipate_44	5.5-5.8	-0.158	10	300	274	523
di(2-ethylhexyl) adipate_45	5.5-5.8	-0.158	10	300	274	474
di(2-ethylhexyl) azelate_48	5.5-5.8	-0.158	10	300	193	258
di(2-ethylhexyl) azelate_49	5.5-5.8	-0.158	10	300	193	251
di(2-ethylhexyl) isophthalate_11	5.5-5.8	-0.158	10	300	133	787
di(2-ethylhexyl) isophthalate_12	5.5-5.8	-0.158	10	300	133	481
di(2-ethylhexyl) maleate_37	5.5-5.8	-2.655	10	100	27	224
di(2-ethylhexyl) maleate_38	5.5-5.8	-2.655	10	100	27	230
di(2-ethylhexyl) phthalate_34	5.5-5.8	-2.655	10	100	66	193
di(2-ethylhexyl) phthalate_35	5.5-5.8	-2.655	10	100	66	185
di(2-ethylhexyl) sebacate_11	5.5-5.8	-0.158	10	300	120	326
di(2-ethylhexyl) sebacate_12	5.5-5.8	-0.158	10	300	120	259
di(n-heptyl,n-nonyl) adipate_46	5.5-5.8	-0.158	10	100	232	381
di(n-heptyl,n-nonyl) adipate_47	5.5-5.8	-0.158	10	100	232	347
di(tridecyl) adipate_42	5.5-5.8	-0.158	10	300	199	486
di(tridecyl) adipate_43	5.5-5.8	-0.158	10	300	199	505
dibutyl fumerate_69	5.5-5.8	-0.158	10	300	321	797
dibutyl fumerate_70	5.5-5.8	-0.158	10	300	321	700
dibutyl sebacate_16	5.5-5.8	-0.158	10	300	149	367
dibutyl sebacate_17	5.5-5.8	-0.158	10	300	149	378
dicapryl adipate_40	5.5-5.8	-0.158	10	300	151	418
dicapryl adipate_41	5.5-5.8	-0.158	10	300	151	377
dicapryl phthalate_32	5.5-5.8	-2.655	10	100	238	360
dicapryl phthalate_33	5.5-5.8	-2.655	10	100	238	379
dicyclohexyl phthalate_72	5.5-5.8	-0.158	10	300	73	148
dicyclohexyl phthalate_73	5.5-5.8	-0.158	10	300	73	223
diethyl phthalate_36	5.5-5.8	-2.655	10	100	499	317
diethyl phthalate_37	5.5-5.8	-2.655	10	100	499	326
diethyl succinate_61	5.5-5.8	-0.158	10	300	527	492
diethyl succinate_62	5.5-5.8	-0.158	10	300	527	490

diethylene glycol dibenzoate_02	5.5-5.8	-2.655	10	100	78	432
diethylene glycol dibenzoate_03	5.5-5.8	-2.655	10	100	78	526
dihexyl phthalate_38	5.5-5.8	-2.655	10	100	370	586
dihexyl phthalate_39	5.5-5.8	-2.655	10	100	370	637
diisobutyl adipate_38	5.5-5.8	-0.158	10	300	535	617
diisobutyl adipate_39	5.5-5.8	-0.158	10	300	535	597
diisobutyl phthalate_42	5.5-5.8	-2.655	10	100	278	223
diisobutyl phthalate_43	5.5-5.8	-2.655	10	100	278	224
diisodecyl adipate_37	5.5-5.8	-0.158	10	300	145	639
diisodecyl adipate_50	5.5-5.8	-0.158	10	300	145	629
diisodecyl azelate_54	5.5-5.8	-0.158	10	300	26	140
diisodecyl azelate_55	5.5-5.8	-0.158	10	300	26	129
diisodecyl phthalate_44	5.5-5.8	-2.655	10	100	227	165
diisodecyl phthalate_45	5.5-5.8	-2.655	10	100	227	172
diisoheptyl phthalate_46	5.5-5.8	-2.655	10	100	79	148
diisoheptyl phthalate_47	5.5-5.8	-2.655	10	100	79	153
diisononyl adipate_34	5.5-5.8	-0.158	10	300	140	604
diisononyl adipate_35	5.5-5.8	-0.158	10	300	140	556
diisononyl phthalate_48	5.5-5.8	-2.655	10	100	421	481
diisononyl phthalate_49	5.5-5.8	-2.655	10	100	421	456
diisooctyl azelate_56	5.5-5.8	-0.158	10	300	31	144
diisooctyl azelate_57	5.5-5.8	-0.158	10	300	31	138
diisooctyl phthalate_51	5.5-5.8	-2.655	10	100	123	193
diisooctyl phthalate_52	5.5-5.8	-2.655	10	100	123	199
dimethyl azelate_58	5.5-5.8	-0.158	10	300	168	303
dimethyl azelate_59	5.5-5.8	-0.158	10	300	168	292
dimethyl isophthalate_70	5.5-5.8	-0.158	10	300	250	1021
dimethyl isophthalate_71	5.5-5.8	-0.158	10	300	250	1094
dimethyl phthalate_53	5.5-5.8	-2.655	10	100	303	532
dimethyl phthalate_54	5.5-5.8	-2.655	10	100	303	546
dimethyl sebacate_18	5.5-5.8	-0.158	10	300	18	632
dimethyl sebacate_29	5.5-5.8	-2.655	10	100	18	506
di-n-butyl maleate_35	5.5-5.8	-2.655	10	100	22	207
di-n-butyl maleate_36	5.5-5.8	-2.655	10	100	22	214

di-n-butyl phthalate_30	5.5-5.8	-2.655	10	100	162	253
di-n-butyl phthalate_31	5.5-5.8	-2.655	10	100	162	211
di-n-hexyl azelate_50	5.5-5.8	-0.158	10	300	158	269
di-n-hexyl azelate_52	5.5-5.8	-0.158	10	300	158	260
diphenyl isophthalate_06	5.5-5.8	-2.655	10	100	35	418
diphenyl isophthalate_07	5.5-5.8	-2.655	10	100	35	429
dipropylene glycol dibenzoate_05	5.5-5.8	-2.655	10	100	462	1264
dipropylene glycol dibenzoate_06	5.5-5.8	-2.655	10	100	462	1201
ditridecyl phthalate_55	5.5-5.8	-2.655	10	100	172	200
ditridecyl phthalate_56	5.5-5.8	-2.655	10	100	172	184
diundecyl phthalate_58	5.5-5.8	-2.655	10	100	182	215
diundecyl phthalate_59	5.5-5.8	-2.655	10	100	182	217
epoxidized linseed oil_60	5.5-5.8	-0.158	10	300	170	367
epoxidized linseed oil_61	5.5-5.8	-0.158	10	300	170	373
epoxidized soy bean oil_62	5.5-5.8	-0.158	10	300	165	274
epoxidized soy bean oil_63	5.5-5.8	-0.158	10	300	165	259
ethylene glycol monostearate_65	5.5-5.8	-0.158	10	300	356	232
ethylene glycol monostearate_66	5.5-5.8	-0.158	10	300	356	220
glycerol monooleate_82	5.5-5.8	-0.158	15	300	39	113
glycerol monooleate_83	5.5-5.8	-0.158	15	300	39	159
glycerol monooleate_84	5.5-5.8	-0.158	15	300	39	106
glycerol monostearate_17	5.5-5.8	-0.158	15	300	188	213
glycerol monostearate_18	5.5-5.8	-0.158	15	300	188	234
glycerol trioleate_93	5.5-5.8	-0.158	15	300	433	400
glycerol trioleate_94	5.5-5.8	-0.158	15	300	433	408
glyceryl triacetate_97	5.5-5.8	-0.158	10	300	153	489
glyceryl triacetate_98	5.5-5.8	-0.158	10	300	153	336
glyceryl tri-acetyl ricinoleate_10	5.5-5.8	-0.158	10	300	222	226
glyceryl tri-acetyl ricinoleate_11	5.5-5.8	-0.158	10	300	222	211
isodecyl diphenyl phosphate_04	5.5-5.8	-0.343	10	300	635	1946
isodecyl diphenyl phosphate_05	5.5-5.8	-0.343	10	300	635	1988
isopropyl isostearate_08	5.5-5.8	-0.158	10	300	24	339
isopropyl isostearate_09	5.5-5.8	-0.158	10	300	24	311
isopropyl myristate_12	5.5-5.8	-0.158	10	300	281	333

isopropyl myristate_13	5.5-5.8	-0.158	10	300	281	378
isopropyl palmitate_89	5.5-5.8	-0.158	10	300	246	325
isopropyl palmitate_90	5.5-5.8	-0.158	10	300	246	338
methyl acetyl ricinoleate_13	5.5-5.8	-0.158	10	300	156	157
methyl acetyl ricinoleate_49	5.5-5.8	-0.158	10	300	156	144
methyl laurate_30	5.5-5.8	-2.655	10	100	24	144
methyl laurate_31	5.5-5.8	-2.655	10	100	24	138
methyl linoleate_03	5.5-5.8	-0.343	10	300	106	272
methyl linoleate_04	5.5-5.8	-0.343	10	300	106	339
methyl oleate_85	5.5-5.8	-0.158	15	300	174	149
methyl oleate_86	5.5-5.8	-0.158	15	300	174	160
methyl palmitate_13	5.5-5.8	-2.655	10	300	76	87
methyl palmitate_14	5.5-5.8	-2.655	10	300	76	114
methyl ricinoleate_14	5.5-5.8	-0.158	15	300	212	114
methyl ricinoleate_17	5.5-5.8	-0.158	15	300	212	108
methyl stearate_12	5.5-5.8	-0.343	15	300	40	95
methyl stearate_13	5.5-5.8	-0.158	10	300	40	77
methyl stearate_15	5.5-5.8	-0.158	10	300	40	90
n-butyl acetyl ricinoleate_12	5.5-5.8	-0.158	15	300	151	89
n-butyl acetyl ricinoleate_13	5.5-5.8	-0.158	15	300	151	97
n-butyl stearate_63	5.5-5.8	-0.158	10	300	184	212
n-butyl stearate_64	5.5-5.8	-0.158	10	300	184	256
n-ethyl,p-toluene sulfonamide_45	5.5-5.8	-2.655	10	100	119	107
n-ethyl,p-toluene sulfonamide_46	5.5-5.8	-2.655	10	100	119	109
n-propyl oleate_89	5.5-5.8	-0.158	15	300	323	144
n-propyl oleate_90	5.5-5.8	-0.158	15	300	323	150
o,p-toluene sulfonamide_12	5.5-5.8	-2.655	10	100	13	346
o,p-toluene sulfonamide_19	5.5-5.8	-0.158	10	100	13	598
polyethyleneglycol dibenzoate_08	5.5-5.8	-2.655	10	100	503	1825
polyethyleneglycol dibenzoate_09	5.5-5.8	-2.655	10	100	503	2023
propylene glycol monostearate_68	5.5-5.8	-0.158	10	300	219	242
propylene glycol monostearate_69	5.5-5.8	-0.158	10	300	219	281
propylene glycol ricinoleate_17	5.5-5.8	-0.158	10	300	196	279
propylene glycol ricinoleate_18	5.5-5.8	-0.158	10	300	196	269

t-butylphenyl diphenyl phosphate_12	5.5-5.8	-0.343	10	300	326	375
t-butylphenyl diphenyl phosphate_13	5.5-5.8	-0.343	10	300	326	351
tetrahydrofurfuryl oleate_91	5.5-5.8	-0.158	15	300	110	110
tetrahydrofurfuryl oleate_92	5.5-5.8	-0.158	15	300	110	113
tri(n-octyl,n-decyl)) trimellitate_13	5.5-5.8	-0.158	10	300	262	416
tri(n-octyl,n-decyl)) trimellitate_19	5.5-5.8	-0.158	10	300	262	443
tri-butoxyethyl phosphate_06	5.5-5.8	-0.343	10	300	464	398
tri-butoxyethyl phosphate_07	5.5-5.8	-0.343	10	300	464	363
tributyl phosphate_08	5.5-5.8	-0.343	10	300	325	561
tributyl phosphate_09	5.5-5.8	-0.343	10	300	325	557
tricapryl trimellitate_13	5.5-5.8	-0.158	10	300	427	357
tricapryl trimellitate_14	5.5-5.8	-0.158	10	300	427	391
tricresyl phosphate_10	5.5-5.8	-0.343	10	300	464	781
tricresyl phosphate_11	5.5-5.8	-0.343	10	300	464	812
triethyl citrate_77	5.5-5.8	-0.158	10	300	248	295
triethyl citrate_78	5.5-5.8	-0.158	10	300	248	266
triisodecyl trimellitate_15	5.5-5.8	-0.158	10	300	399	555
triisodecyl trimellitate_16	5.5-5.8	-0.158	10	300	399	551
triisononyl trimellitate_17	5.5-5.8	-0.158	10	300	331	318
triisononyl trimellitate_18	5.5-5.8	-0.158	10	300	331	313
tri-n-butyl citrate_52	5.5-5.8	-0.158	10	300	284	204
tri-n-butyl citrate_53	5.5-5.8	-0.158	10	300	284	200
triphenyl phosphate_14	5.5-5.8	-0.343	10	300	391	759
triphenyl phosphate_15	5.5-5.8	-0.343	10	300	391	803

[a]The sample names are presented identical to their naming convention in the TGRS plasticizer library. The number, originally indicating the nth measurement collected during a measurement session was retained to ensure unique distinction between replicates.

V. Time-gated Raman data processing

Calibration

The wavenumber range of the measurements were calibrated using a polystyrene reference sample (PSRS) by calculating a shift factor using a weighted average comparing the locations of 4 primary peaks against documented peak locations and intensities reported in McCreery 2000 (**Table S5**).[1] A piece of PS was measured as the first sample in every measurement sample to serve as the calibration reference.

Table S5. Raman peak locations of polystyrene and normalized intensities used as weights

	Peak 1	Peak 2	Peak 3	Peak 4
Standardized location (cm^{-1})	620.9	1001.4	1031.8	1602.3
Normalized intensity (0:100)	16	100	27	28

The calibration shift factor represents a weighted average of the deviation between the peak locations of the measured PSRS and standardized PS peak locations. Four peaks were assessed because the as-measured peaks presented some variation in their shifted position relative to the standardized peak locations. The average deviation of the peaks was calculated and weighted relative to the corresponding peak intensities as reported in (**Table S5**).[1]

For further details, please see the NIST Public Data Repository page and available spectra files. The as-measured peaks of the PSRS and the calculated shift factors are printed in the header lines of the unprocessed data files. Implementation of the data calibration step can also be observed in the companion data processing code hosted in the usnistgov GitHub: https://github.com/usnistgov/TGRS-Plasticizer-Library.

Calculating Signal-to-Noise Ratio (SNR)

SNR was calculated as specified by McCreery (**eqn. S1**).[2] Briefly, successive replicates of each compound were subtracted to remove contributions from the Raman band and background features to investigate the contribution of noise to the signal. SNR was calculated about the highest intensity Raman peak (Normalized peak height of 1 before background subtraction).

$$SNR = \frac{Y_{avg}}{(\sigma_{noise}/\sqrt{2})} \qquad \text{eqn. S1}$$

Where Y_{avg} is an average of the peak height above background of the highest-intensity Raman band across successive replicates. σ_{noise} is the average of the standard deviation (STD) of the noise calculated under the footprint of the highest-intensity Raman band from each replicate. σ_{noise} was calculated from a 24 wavenumber range about the peak maximum. Before determination of the SNR, each spectra was normalized in intensity between 0 and 1, and a background subtraction was performed using an asymmetric least squares smoothing algorithm.[3]

Calculating Signal-to-Background Ratio (SBR)

The SBR is an indication of peak strength relative to the noise about the spectral background (**eqn. S2**). The calculated SBR is a less rigorous approximation of the SNR that can be calculated using a single spectrum as opposed to the multiple replicates required for proper SNR calculations.[2]

$$SBR = \frac{Y}{(2\sigma_{background:noise})} \qquad \textbf{eqn. S2}$$

Where Y is the height of the tallest Raman peak above the spectral background, and $\sigma_{background:noise}$ is the standard deviation of noise about the longest stretch of background without any peaks. The spectral background was fit using an asymmetric least squares smoothing algorithm[3] and the STD of the background noise was calculated about the flattened background.

VI. LC-MS sample preparation and measurement conditions

A brief overview of the mass spectral library-building process is provided in **Figure S1.** Sample preparation was performed as described in the Materials and Methods section. In addition to what is described in the Methods section, we must clarify that during the generation of raw data for the libraries, many compounds are prepared simultaneously, and the solution concentrations are approximately micromolar rather than exact, which would be very time-consuming. If the ionization or solubility is poor, the solvent or concentration can be varied, and it is usually reported in the library spectrum comment field (See Solutions field in **Table S6**). In general, the plasticizers discussed in this work ionize well by APCI, even at nanomolar concentrations.

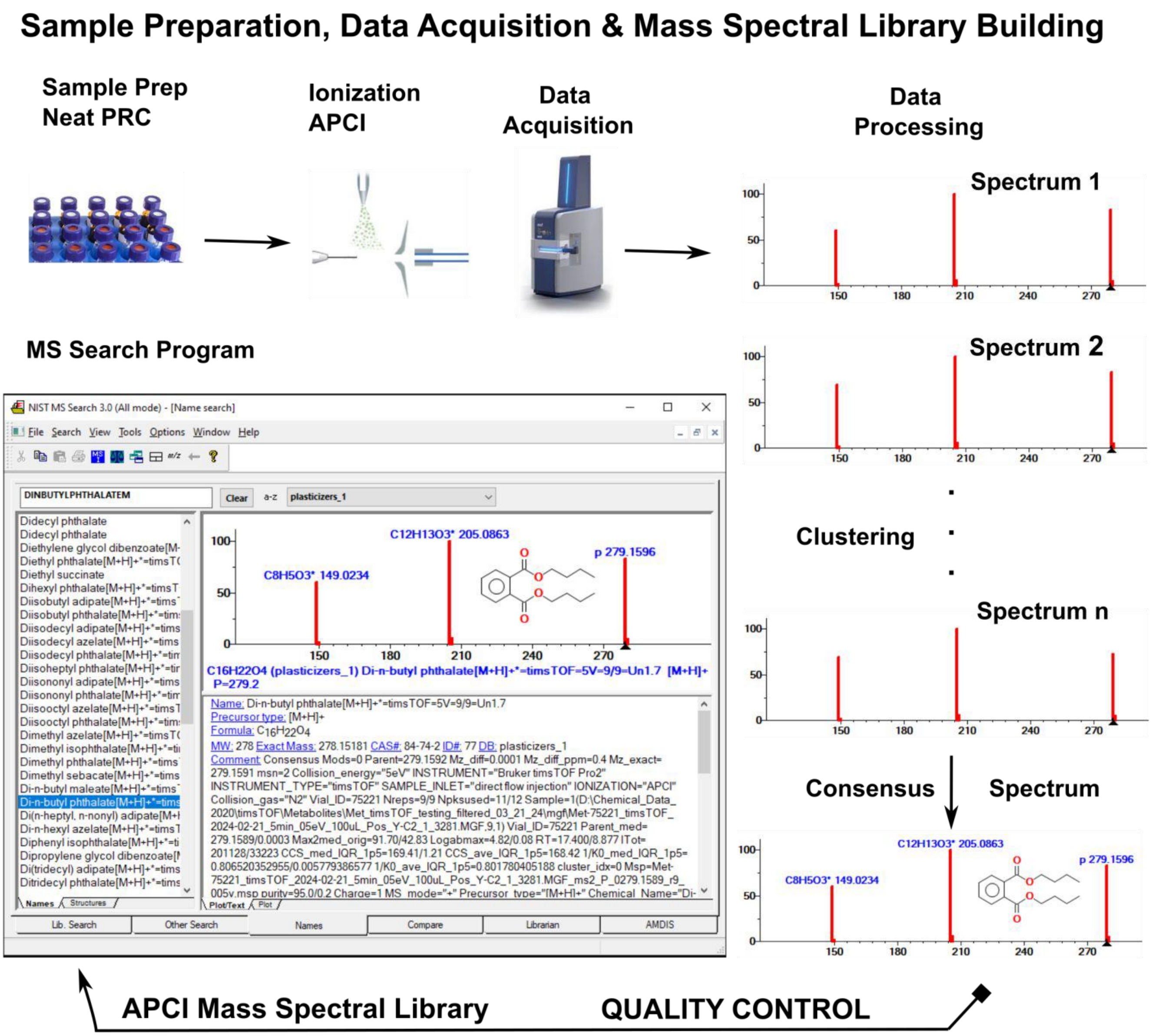

Figure S1. Workflow for building the APCI tandem mass spectral library.

VII. Instructions for using NIST Mass Spectral Library tools

The same data is presented in two ways in the published MS plasticizer dataset in the NIST Data Repository to support different user applications. First, tandem mass spectra and curated chemical structures of individual compounds, obtained using a VIP-HESI-APCI ion source on a Bruker timsTOF Pro, are provided in separate MGF files, along with the corresponding SDF structures. Both formats are ASCII and can be read and parsed with a common editor or managed with the NIST Search Software (NIST browser). This may be useful when the user needs individual spectra or wants to process part of the data using a computer. Second, a zip file (*PlasticizerFiles.zip*) with all the compounds and metadata in one file, containing a searchable library in the NIST format. The latter can only be managed using the NIST browser. To use the library, the NIST browser must be installed first. The installation is very straightforward. Go to:

https://chemdata.nist.gov/dokuwiki/doku.php?id=chemdata:nistlibs

under **NIST Search Software,** download the installation program and the manual. More details can be found in the browser manual. During installation, the user decides where the program is installed. Inside that folder, the installation program creates another folder named MSSEARCH.

Importing plasticizer library into the NIST Search Software

In order to open the plasticizer library files in the NIST Search Software, locate the installation folder. In the MSSEARCH folder, create a new folder with the desired name of the library, and copy the contents of *PlasticizerFiles.zip* into it (**Figure S2**). Reopen the NIST browser (nistms.exe), and the library is ready to access.

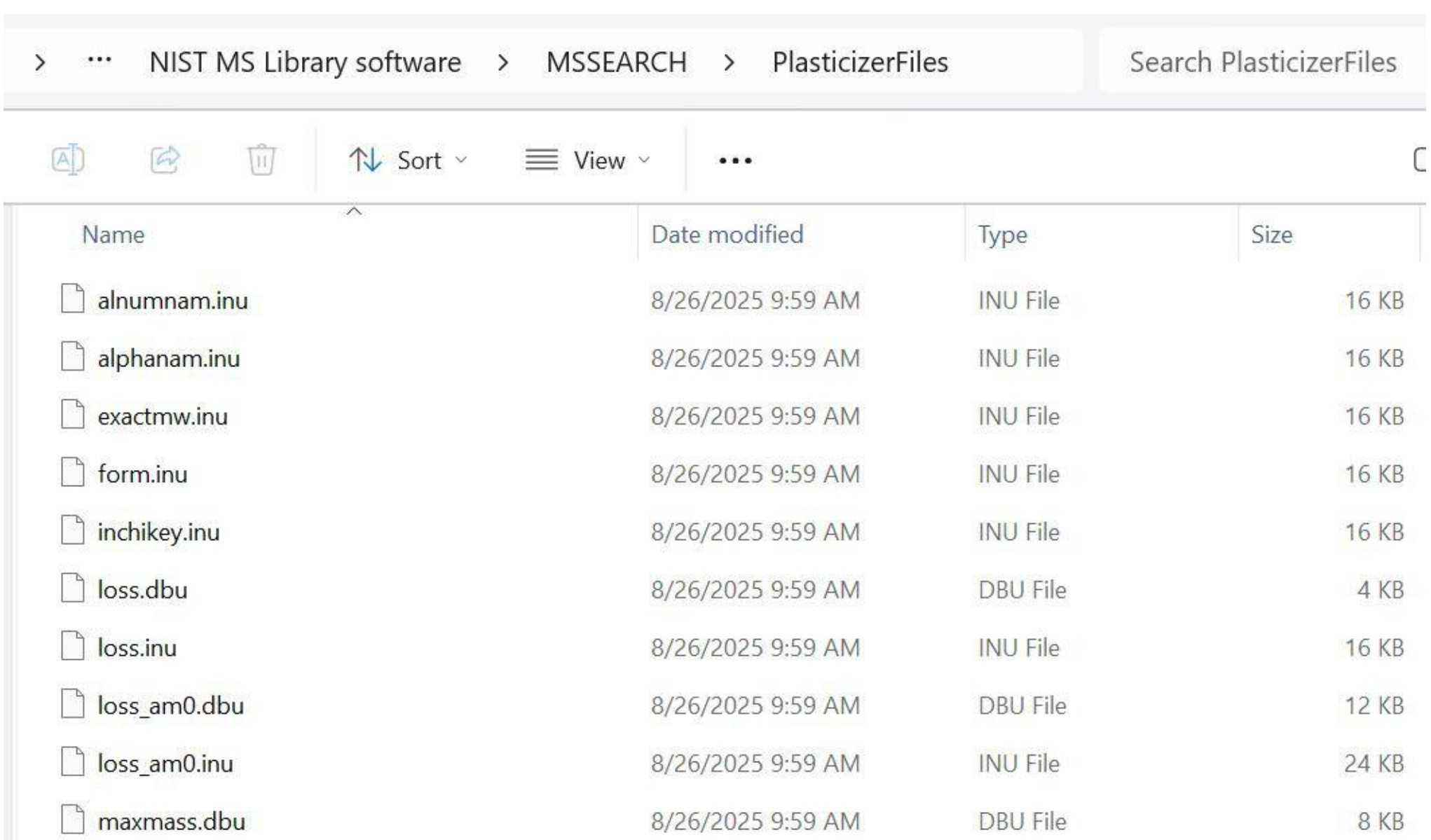

Figure S2. Screenshot demonstrating file organization for uploading a spectral library to the NIST Search Software on Windows OS

To use the library to identify compounds from experimental data, the raw data must be processed and the MS/MS spectra exported in an appropriate format, typically MGF before opening in the NIST Search Software. Perhaps the most straightforward way to do this is to use MSConvert from ProteoWizard: https://proteowizard.sourceforge.io/.

Once the MGF files are available, they can be opened in the NIST browser by selecting File -> Open, and the query spectra appear in the upper-left window (**Figure S3**). Double-click on a particular query spectrum, and the software searches for the best match in the mass spectral library (or libraries) previously defined in the library search options. To find the library search options, make sure the highlighted tab at the bottom of the browser is 'Lib. Search'. Then go to the top and click the icon with the spectrum:

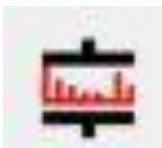

A new pop-up window appears; click the 'Libraries' tab to add or remove libraries for the current search. Most of the other fields have a straightforward meaning, but it is recommended to read the manual for more specific searches. Regarding this work, the most important search types are Identity MS/MS and Similarity: Hybrid MS/MS.

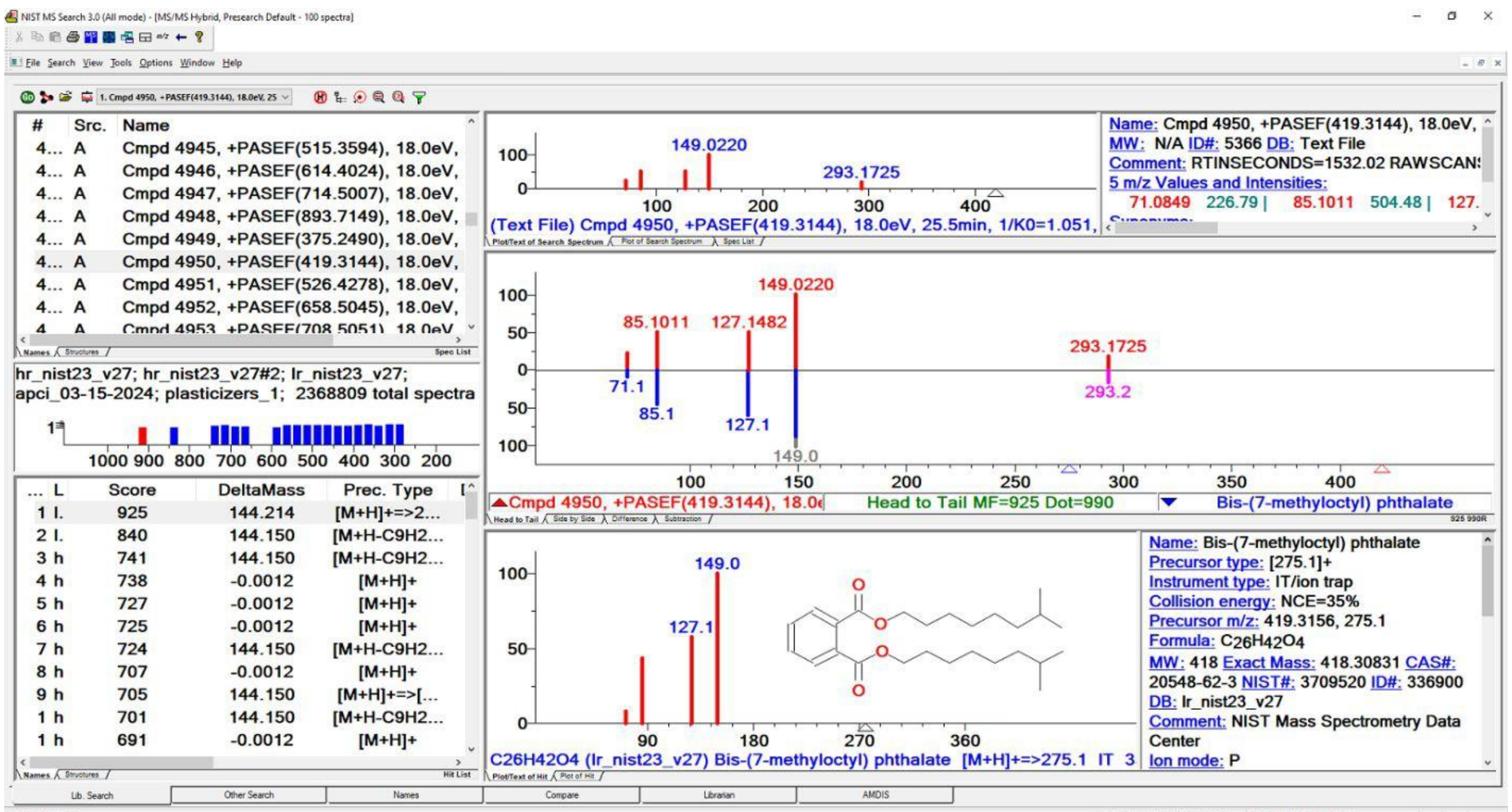

Figure S3. Enhancing the scope of library identification using hybrid searching.

As stated in the manuscript, the identity search uses the full spectrum, and the similarity search, also known as a hybrid search, removes the constraint on the parent ion mass-to-charge ratio (m/z), allowing the query spectrum to match spectra with different parent ion m/z values. The difference between the query parent ion m/z and the reference matched spectra is called *Deltamass* and usually corresponds to a reasonable neutral loss. For example, **Figure S3** shows a hybrid search in which the highlighted experimental spectrum matched, with the highest score, a fragment of Bis(7-methyloctyl) phthalate (m/z 149) with a *Deltamass* of 144 Da and an octyl group. The pink peak in the reference spectrum (head-to-tail comparison at the central window of **Figure S3**) is generated by the sum of 144 and 149. The fragment at m/z 149 is present in all phthalates, so this type of identification usually points to the chemical class of the query spectrum and needs additional information to confirm full identification. For the exact description of the browser and search option, consult the manual.

VIII. Mass spectrometry library measurement conditions

Instruments‡: A – Thermo Finnigan Elite Orbitrap, B – Bruker timsTOF Pro2, C- Agilent QTOF 6530, D- Orbitrap Fusion Lumos Upgrade, E - Thermo Finnigan Velos Orbitrap

Comments: Descriptions of the fields in the Mass Spectral Library comments can be found in the library manual or through the help function in the NIST MS Search browser (installation detailed in **SI VII**)

Table S6. Measurement conditions for plasticizer mass spectral library

Sample Name	Instrument Type	Instrument	Ionization	Col. Gas	Col. Energy (eV)	Precursor Type	Exact Mass (g/mol)	Solution (µmol/L)	Comments
2,2,4-Trimethyl-1,3-pentanediol diisobutyrate	HCD	A	ESI	N2	NCE=50 % 30	[M+Na]+	286.2144	water/acetonitril/ formic acid (50/50/0.1)	Mz_diff=-2.9ppm
2-Ethylhexyl diphenyl phosphate	timsTOF	B	APCI	N2	10	[M+H]+	362.1647	methanol/water/ formic acid (98/2/0.1)	Mods=0 Parent=363.1718 Mz_diff=-0.0002 Mz_diff_ppm=-0.6 Mz_exact=363.1720 msn=2 Nreps=13/13 Npksused=7 Parent_med=363.1716/0.0008 Max2med_orig=174.40/52.38 Logabmax=6.41/0.06 RT=35.490/1.712 ITot=2796959/444312 CCS_med_IQR_1p5=188.90/0.40 CCS_ave_IQR_1p5=188.73 1/K0_med_IQR_1p5=0.908924210898/0.001917867864 1/K0_ave_IQR_1p5=0.908093215215
2-Ethylhexyl diphenyl phosphate	IT/ion trap	A	ESI	He	NCE=35 %	[M+H]+	362.1647	water/acetonitrile/ formic acid (50/50/0.1)	Nreps=22/22
2-Ethylhexyl sebacate	Q-TOF	C	APCI	N2	5	[M+H]+	426.3709	methanol/water/ formic acid (98/2/0.1)	Mz_diff=17.8ppm Nreps=19/19
Acetyl triethyl citrate	timsTOF	B	APCI	N2	10	[M+H]+	318.1315	methanol/water/ formic acid (98/2/0.1)	Mods=0 Parent=319.1388 Mz_diff=0.0001 Mz_diff_ppm=0.3 Mz_exact=319.1387 msn=2 Nreps=6/6 Npksused=41/42 Parent_med=319.1389/0.0003

									Max2med_orig=118.75/27.67<br>Logabmax=6.28/0.29<br>RT=20.530/9.385<br>ITot=8337422/3889568<br>CCS_med_IQR_1p5=167.57/0.14<br>CCS_ave_IQR_1p5=167.57<br>1/K0_med_IQR_1p5=0.802324683<br>670/0.000649953010<br>1/K0_ave_IQR_1p5=0.8023246836<br>70
Acetyl tri-n-butyl citrate	timsTOF	B	APCI	N2	10	[M+H]+	402.2254	methanol/water/<br>formic acid<br>(98/2/0.1)	Mods=0 Parent=403.2328<br>Mz_diff=0.0002 Mz_diff_ppm=0.5<br>Mz_exact=403.2326 msn=2<br>Nreps=9/11 Npksused=46<br>Parent_med=403.2326/0.0003<br>Max2med_orig=172.10/46.76<br>Logabmax=6.57/0.36<br>RT=15.300/9.931<br>ITot=15069535/5286658<br>CCS_med_IQR_1p5=200.54/0.20<br>CCS_ave_IQR_1p5=200.49<br>1/K0_med_IQR_1p5=0.968370708<br>184/0.000976941496<br>1/K0_ave_IQR_1p5=0.9681563120<br>62
Butyl oleate	timsTOF	B	APCI	N2	15	[M+H]+	338.3185	methanol/water/<br>formic acid<br>(98/2/0.1)	Mods=0 Parent=339.3259<br>Mz_diff=0.0001 Mz_diff_ppm=0.3<br>Mz_exact=339.3258 msn=2<br>Nreps=2/20 Npksused=72/118<br>Parent_med=339.3259/0.0005<br>Max2med_orig=25.35/8.25<br>Logabmax=5.27/0.32<br>RT=24.280/15.650<br>ITot=2050220/1296364<br>CCS_med_IQR_1p5=203.57/0.23<br>CCS_ave_IQR_1p5=203.57<br>1/K0_med_IQR_1p5=0.977074288<br>941/0.001091629008<br>1/K0_ave_IQR_1p5=0.9770742889<br>41
Butyl oleate	HCD	A	ESI	N2	10<br>NCE=1<br>5 %	[M+H]+	338.3185	Acetonitrile/Wat<br>er/<br>Formic acid	spectrum mz_diff=-0.0013;<br>Nreps=2/2

Butyl ricinoleate	timsTOF	B	APCI	N2	15	[M+H-H2O]+	354.3134	methanol/water/formic acid (98/2/0.1)	Mods=0 Parent=337.3073 Mz_diff=-0.0028 Mz_diff_ppm=-8.3 Mz_exact=337.3101 msn=2 Nreps=7/12 Npksused=126/127 Parent_med=337.3074/0.0007 Max2med_orig=33.40/7.61 Logabmax=6.00/0.04 RT=20.350/7.831 ITot=6714417/619859 CCS_med_IQR_1p5=197.53/0.91 CCS_ave_IQR_1p5=197.87 1/K0_med_IQR_1p5=0.947861830271/0.004348076658 1/K0_ave_IQR_1p5=0.949479048993
Butylbenzyl phthalate	timsTOF	B	APCI	N2	5	[M+H]+	312.1362	methanol/water/formic acid (98/2/0.1)	Mods=0 Parent=313.1435 Mz_diff=0.0001 Mz_diff_ppm=0.3 Mz_exact=313.1434 msn=2 Nreps=7/7 Npksused=19 Parent_med=313.1435/0.0005 Max2med_orig=109.50/21.06 Logabmax=4.81/0.09 RT=22.500/10.599 ITot=144979/24151 CCS_med_IQR_1p5=178.28/0.08 CCS_ave_IQR_1p5=178.31 1/K0_med_IQR_1p5=0.852975405250/0.000398853422 1/K0_ave_IQR_1p5=0.853120166673
Butyloctyl phthalate	timsTOF	B	APCI	N2	5	[M+H-C4H8]+	334.2144	methanol/water/formic acid (98/2/0.1)	Mods=0 Parent=279.1593 Mz_diff=0.0002 Mz_diff_ppm=0.7 Mz_exact=279.1591 msn=2 Nreps=40/45 Npksused=26/28 Parent_med=279.1596/0.0007 Max2med_orig=207.55/97.79 Logabmax=5.78/0.07 RT=29.630/8.416 ITot=1819897/205347 CCS_med_IQR_1p5=169.21/0.29 CCS_ave_IQR_1p5=169.22 1/K0_med_IQR_1p5=0.805562485786/0.001400428473 1/K0_ave_IQR_1p5=0.805583930904

Di(2-ethylhexyl) adipate	timsTOF	B	APCI	N2	10	[M+H]+	370.3083	methanol/water/ formic acid (98/2/0.1)	Mods=0 Parent=371.3159 Mz_diff=0.0003 Mz_diff_ppm=0.8 Mz_exact=371.3156 msn=2 Nreps=7/7 Npksused=22/23 Parent_med=371.3160/0.0002 Max2med_orig=73.80/10.64 Logabmax=6.60/0.26 RT=21.460/10.776 ITot=13844400/3584102 CCS_med_IQR_1p5=210.64/0.20 CCS_ave_IQR_1p5=210.73 1/K0_med_IQR_1p5=1.014336485 508/0.000953147541 1/K0_ave_IQR_1p5=1.0147647266 04
Di(2-ethylhexyl) adipate	HCD	D	ESI	N2	10 NCE=5 %	[M+H]+	370.3083	water/acetonitril e/ formic acid (50/50/0.1)	Nreps=29/29 Mz_diff=2.7ppm
Di(2-ethylhexyl) azelate	timsTOF	B	APCI	N2	10	[M+H]+	412.3553	methanol/water/ formic acid (98/2/0.1)	Mods=0 Parent=413.3626 Mz_diff=0.0001 Mz_diff_ppm=0.2 Mz_exact=413.3625 msn=2 Nreps=10/11 Npksused=32/35 Parent_med=413.3627/0.0003 Max2med_orig=98.10/54.22 Logabmax=6.52/0.33 RT=18.005/11.443 ITot=12530927/5330946 CCS_med_IQR_1p5=218.52/1.52 CCS_ave_IQR_1p5=217.40 1/K0_med_IQR_1p5=1.056039040 794/0.007326055440 1/K0_ave_IQR_1p5=1.0506194452 82
Di(2-ethylhexyl) isophthalate	timsTOF	B	APCI	N2	5	[M+H]+	390.277	methanol/water/ formic acid (98/2/0.1)	Mods=0 Parent=391.2854 Mz_diff=0.0011 Mz_diff_ppm=2.8 Mz_exact=391.2843 msn=2 Nreps=2/2 Npksused=3/6 Parent_med=391.2854/0.0000 Max2med_orig=3.35/2.25 Logabmax=4.02/0.03 RT=26.185/0.205 ITot=25771/5054 CCS_med_IQR_1p5=219.24/0.00 CCS_ave_IQR_1p5=219.24 1/K0_med_IQR_1p5=1.057645691

									809/0.000000000000<br>1/K0_ave_IQR_1p5=1.0576456918<br>09
Di(2-ethylhexyl) maleate	timsTOF	B	APCI	N2	10	[M+H]+	340.2614	methanol/water/ formic acid (98/2/0.1)	Mods=0 Parent=341.2676<br>Mz_diff=-0.0010 Mz_diff_ppm=-2.9<br>Mz_exact=341.2686 msn=2<br>Nreps=14/14 Npksused=16<br>Parent_med=341.2674/0.0005<br>Max2med_orig=98.20/129.49<br>Logabmax=4.93/0.71<br>RT=25.905/10.752<br>ITot=249126/276285<br>CCS_med_IQR_1p5=199.84/3.78<br>CCS_ave_IQR_1p5=203.07<br>1/K0_med_IQR_1p5=0.959374007<br>301/0.018127904255<br>1/K0_ave_IQR_1p5=0.9748939245<br>55
Di(2-ethylhexyl) phthalate	timsTOF	B	APCI	N2	15	[M+H]+	390.277	methanol/water/ formic acid (98/2/0.1)	Mods=0 Parent=391.2830<br>Mz_diff=-0.0013 Mz_diff_ppm=-3.3<br>Mz_exact=391.2843 msn=2<br>Nreps=7/7 Npksused=25<br>Parent_med=391.2828/0.0004<br>Max2med_orig=457.70/53.37<br>Logabmax=7.17/0.19<br>RT=21.730/11.023<br>ITot=27624931/4746902<br>CCS_med_IQR_1p5=210.96/0.10<br>CCS_ave_IQR_1p5=211.02<br>1/K0_med_IQR_1p5=1.017700311<br>947/0.000474757402<br>1/K0_ave_IQR_1p5=1.0180069186<br>45
Di(2-ethylhexyl) sebacate	timsTOF	B	APCI	N2	15	[M+H]+	426.3709	methanol/water/ formic acid (98/2/0.1)	Mods=0 Parent=427.3740<br>Mz_diff=-0.0042 Mz_diff_ppm=-9.8<br>Mz_exact=427.3782 msn=2<br>Nreps=10/10 Npksused=29<br>Parent_med=427.3741/0.0003<br>Max2med_orig=569.75/253.27<br>Logabmax=7.11/0.48<br>RT=22.745/10.686<br>ITot=22298734/11657818<br>CCS_med_IQR_1p5=219.73/0.22<br>CCS_ave_IQR_1p5=219.74<br>1/K0_med_IQR_1p5=1.063004883

									680/0.001044292046<br>1/K0_ave_IQR_1p5=1.0630553878<br>86
Di(n-heptyl,n-nonyl) adipate	timsTOF	B	APCI	N2	18	[M+H]+	370.3083	methanol/water/ formic acid (98/2/0.1)	Mods=0 Parent=371.3158<br>Mz_diff=0.0002 Mz_diff_ppm=0.5<br>Mz_exact=371.3156 msn=2<br>Nreps=12/14 Npksused=32<br>Parent_med=371.3161/0.0005<br>Max2med_orig=130.75/71.92<br>Logabmax=6.02/0.24<br>RT=22.135/11.250<br>ITot=1971353/1183682<br>CCS_med_IQR_1p5=211.92/1.88<br>CCS_ave_IQR_1p5=212.73<br>1/K0_med_IQR_1p5=1.020494102<br>292/0.009053760830<br>1/K0_ave_IQR_1p5=1.0243972527<br>90
Di(tridecyl) adipate	timsTOF	B	APCI	N2	18	[M+H]+	510.4648	methanol/water/ formic acid (98/2/0.1)	Mods=0 Parent=511.4712<br>Mz_diff=-0.0009 Mz_diff_ppm=-1.8<br>Mz_exact=511.4721 msn=2<br>Nreps=15/17 Npksused=54/61<br>Parent_med=511.4709/0.0007<br>Max2med_orig=44.10/14.06<br>Logabmax=5.04/0.24<br>RT=21.140/10.739<br>ITot=617341/361973<br>CCS_med_IQR_1p5=251.45/1.38<br>CCS_ave_IQR_1p5=251.21<br>1/K0_med_IQR_1p5=1.222674776<br>589/0.006705275650<br>1/K0_ave_IQR_1p5=1.2214873124<br>63
Dibutyl fumarate	timsTOF	B	APCI	N2	10	[M+H]+	228.1362	methanol/water/ formic acid (98/2/0.1)	Mods=0 Parent=229.1429<br>Mz_diff=-0.0005 Mz_diff_ppm=-2.2<br>Mz_exact=229.1434 msn=2<br>Nreps=3/3 Npksused=18/24<br>Parent_med=229.1430/0.0004<br>Max2med_orig=83.10/9.13<br>Logabmax=6.34/0.01<br>RT=25.310/6.720<br>ITot=5798495/337025<br>CCS_med_IQR_1p5=153.20/0.20<br>CCS_ave_IQR_1p5=153.20<br>1/K0_med_IQR_1p5=0.722172407

									754/0.000961431784 1/K0_ave_IQR_1p5=0.722172407754
Dibutyl itaconate	HCD	A	ESI	N2	4 NCE=10 %	[M+H]+	242.1518	water/acetonitrile/ formic acid (50/50/0.1)	Spec= Nreps=24/24 Mz_diff=-0.4ppm
Dibutyl sebacate	timsTOF	B	APCI	N2	10	[M+H]+	314.2457	methanol/water/ formic acid (98/2/0.1)	Mods=0 Parent=315.2505 Mz_diff=-0.0025 Mz_diff_ppm=-7.9 Mz_exact=315.2530 msn=2 Nreps=9/9 Npksused=31 Parent_med=315.2505/0.0002 Max2med_orig=363.50/124.81 Logabmax=6.96/0.45 RT=20.390/10.277 ITot=22529309/7403247 CCS_med_IQR_1p5=183.43/0.11 CCS_ave_IQR_1p5=183.45 1/K0_med_IQR_1p5=0.877856373005/0.000510446382 1/K0_ave_IQR_1p5=0.877968386161
Dicyclohexyl phthalate	timsTOF	B	APCI	N2	15	[M+H]+	330.1831	methanol/water/ formic acid (98/2/0.1)	Mods=0 Parent=331.1893 Mz_diff=-0.0011 Mz_diff_ppm=-3.3 Mz_exact=331.1904 msn=2 Nreps=7/7 Npksused=22 Parent_med=331.1892/0.0003 Max2med_orig=324.90/84.20 Logabmax=6.98/0.25 RT=21.500/10.791 ITot=20722521/4797000 CCS_med_IQR_1p5=186.22/0.15 CCS_ave_IQR_1p5=186.16 1/K0_med_IQR_1p5=0.892976418232/0.000735951421 1/K0_ave_IQR_1p5=0.892680336301
Didecyl phthalate	Q-TOF	C	APCI	N2	20	[M+H]+	446.3396	methanol/water/ formic acid (98/2/0.1)	Mz_diff=2.9ppm Nreps=2/2
Didecyl phthalate	HCD	D	ESI	N2	10 NCE=5 %	[M+H]+	446.3396	water/acetonitrile/ formic acid (50/50/0.1)	Nreps=25/25 Mz_diff=-2.0ppm

Diethyl phthalate	timsTOF	B	APCI	N2	5	[M+H]+	222.0892	methanol/water/ formic acid (98/2/0.1)	Mods=0 Parent=223.0960 Mz_diff=-0.0005 Mz_diff_ppm=-2.2 Mz_exact=223.0965 msn=2 Nreps=38/38 Npksused=14 Parent_med=223.0959/0.0005 Max2med_orig=184.70/64.48 Logabmax=6.78/0.03 RT=26.545/8.194 ITot=8822669/756671 CCS_med_IQR_1p5=143.11/0.48 CCS_ave_IQR_1p5=143.16 1/K0_med_IQR_1p5=0.673646773959/0.002261219793 1/K0_ave_IQR_1p5=0.673852512536
Diethyl succinate	HCD	A	ESI	N2	3 NCE=10 %	[M+H]+	174.0892	water/acetonitrile/ formic acid (50/50/0.1)	Mz_diff=2.3ppm Nreps=17/21
Diethylene glycol dibenzoate	timsTOF	B	APCI	N2	10	[M+H]+	314.1154	methanol/water/ formic acid (98/2/0.1)	Mods=0 Parent=315.1233 Mz_diff=0.0006 Mz_diff_ppm=1.9 Mz_exact=315.1227 msn=2 Nreps=8/8 Npksused=6 Parent_med=315.1231/0.0003 Max2med_orig=408.95/135.11 Logabmax=5.37/0.14 RT=13.410/6.777 ITot=262393/73835 CCS_med_IQR_1p5=167.77/0.31 CCS_ave_IQR_1p5=167.72 1/K0_med_IQR_1p5=0.802864924072/0.001519857390 1/K0_ave_IQR_1p5=0.802665848830
Dihexyl phthalate	timsTOF	B	APCI	N2	10	[M+H]+	334.2144	methanol/water/ formic acid (98/2/0.1)	Mods=0 Parent=335.2193 Mz_diff=-0.0024 Mz_diff_ppm=-7.2 Mz_exact=335.2217 msn=2 Nreps=11/11 Npksused=24/25 Parent_med=335.2192/0.0006 Max2med_orig=128.20/58.44 Logabmax=5.48/0.44 RT=15.520/9.300 ITot=768508/368875 CCS_med_IQR_1p5=191.87/3.02 CCS_ave_IQR_1p5=189.04

									1/K0_med_IQR_1p5=0.920458611<br>401/0.014475952642<br>1/K0_ave_IQR_1p5=0.9069110590<br>46
Diisobutyl adipate	timsTOF	B	APCI	N2	10	[M+H]+	258.1831	methanol/water/<br>formic acid<br>(98/2/0.1)	Mods=0 Parent=259.1906<br>Mz_diff=0.0002 Mz_diff_ppm=0.8<br>Mz_exact=259.1904 msn=2<br>Nreps=7/7 Npksused=21<br>Parent_med=259.1907/0.0004<br>Max2med_orig=52.50/17.96<br>Logabmax=4.96/0.13<br>RT=21.800/10.731<br>ITot=227653/57786<br>CCS_med_IQR_1p5=168.98/0.19<br>CCS_ave_IQR_1p5=168.99<br>1/K0_med_IQR_1p5=0.801632554<br>638/0.000888839603<br>1/K0_ave_IQR_1p5=0.8017019494<br>22
Diisobutyl phthalate	timsTOF	B	APCI	N2	5	[M+H]+	278.1518	methanol/water/<br>formic acid<br>(98/2/0.1)	Mods=0 Parent=279.1589<br>Mz_diff=-0.0002 Mz_diff_ppm=-0.7<br>Mz_exact=279.1591 msn=2<br>Nreps=7/8 Npksused=16<br>Parent_med=279.1589/0.0002<br>Max2med_orig=71.30/19.24<br>Logabmax=4.73/0.08<br>RT=15.560/10.909<br>ITot=178178/26150<br>CCS_med_IQR_1p5=168.88/0.15<br>CCS_ave_IQR_1p5=168.94<br>1/K0_med_IQR_1p5=0.803992495<br>824/0.000703688878<br>1/K0_ave_IQR_1p5=0.8042775736<br>63
Diisodecyl adipate	timsTOF	B	APCI	N2	18	[M+H]+	426.3709	methanol/water/<br>formic acid<br>(98/2/0.1)	Mods=0 Parent=427.3782<br>Mz_diff=0.0000 Mz_diff_ppm=0.0<br>Mz_exact=427.3782 msn=2<br>Nreps=15/16 Npksused=37/38<br>Parent_med=427.3783/0.0006<br>Max2med_orig=162.60/54.63<br>Logabmax=6.37/0.42<br>RT=21.080/10.777<br>ITot=7505673/4699888<br>CCS_med_IQR_1p5=224.52/2.79<br>CCS_ave_IQR_1p5=225.37

									1/K0_med_IQR_1p5=1.086162845<br>008/0.013496496522<br>1/K0_ave_IQR_1p5=1.0902710445<br>60
Diisodecyl azelate	timsTOF	B	APCI	N2	18	[M+H]+	468.4179	methanol/water/<br>formic acid<br>(98/2/0.1)	Mods=0 Parent=469.4242<br>Mz_diff=-0.0009 Mz_diff_ppm=-1.9<br>Mz_exact=469.4251 msn=2<br>Nreps=26/27 Npksused=48<br>Parent_med=469.4241/0.0009<br>Max2med_orig=81.80/51.89<br>Logabmax=5.98/0.36<br>RT=19.740/9.853<br>ITot=3283620/2356561<br>CCS_med_IQR_1p5=238.69/3.48<br>CCS_ave_IQR_1p5=239.11<br>1/K0_med_IQR_1p5=1.157948014<br>998/0.016885675984<br>1/K0_ave_IQR_1p5=1.1599564294<br>55
Diisodecyl phthalate	timsTOF	B	APCI	N2	15	[M+H]+	446.3396	methanol/water/<br>formic acid<br>(98/2/0.1)	Mods=0 Parent=447.3439<br>Mz_diff=-0.0030 Mz_diff_ppm=-6.7<br>Mz_exact=447.3469 msn=2<br>Nreps=14/14 Npksused=35<br>Parent_med=447.3439/0.0002<br>Max2med_orig=117.70/49.05<br>Logabmax=6.43/0.34<br>RT=21.455/10.759<br>ITot=10166273/6571890<br>CCS_med_IQR_1p5=225.25/2.00<br>CCS_ave_IQR_1p5=227.03<br>1/K0_med_IQR_1p5=1.091205209<br>391/0.009706244342<br>1/K0_ave_IQR_1p5=1.0998237601<br>04
Diisoheptyl phthalate	timsTOF	B	APCI	N2	5	[M+H]+	362.2457	methanol/water/<br>formic acid<br>(98/2/0.1)	Mods=0 Parent=363.2529<br>Mz_diff=-0.0001 Mz_diff_ppm=-0.3<br>Mz_exact=363.2530 msn=2<br>Nreps=7/7 Npksused=21<br>Parent_med=363.2529/0.0001<br>Max2med_orig=251.80/34.81<br>Logabmax=6.44/0.40<br>RT=21.100/10.709<br>ITot=4369353/2637248<br>CCS_med_IQR_1p5=204.42/0.11<br>CCS_ave_IQR_1p5=204.42

									1/K0_med_IQR_1p5=0.983617499<br>567/0.000542977351<br>1/K0_ave_IQR_1p5=0.9836083886<br>61
Diisononyl adipate	timsTOF	B	APCI	N2	18	[M+H]+	398.3396	methanol/water/<br>formic acid<br>(98/2/0.1)	Mods=0 Parent=399.3462<br>Mz_diff=-0.0007 Mz_diff_ppm=-1.8<br>Mz_exact=399.3469 msn=2<br>Nreps=13/14 Npksused=36<br>Parent_med=399.3463/0.0007<br>Max2med_orig=165.10/72.84<br>Logabmax=6.55/0.25<br>RT=23.920/9.164<br>ITot=9432210/3802833<br>CCS_med_IQR_1p5=218.48/1.82<br>CCS_ave_IQR_1p5=219.70<br>1/K0_med_IQR_1p5=1.054669162<br>021/0.008778168218<br>1/K0_ave_IQR_1p5=1.0606043490<br>02
Diisononyl phthalate	timsTOF	B	APCI	N2	5	[M+H]+	418.3083	methanol/water/<br>formic acid<br>(98/2/0.1)	Mods=0 Parent=419.3155<br>Mz_diff=-0.0001 Mz_diff_ppm=-0.2<br>Mz_exact=419.3156 msn=2<br>Nreps=12/12 Npksused=25/26<br>Parent_med=419.3154/0.0004<br>Max2med_orig=248.10/81.87<br>Logabmax=6.65/0.26<br>RT=19.735/10.282<br>ITot=6181810/2279319<br>CCS_med_IQR_1p5=220.94/1.57<br>CCS_ave_IQR_1p5=219.90<br>1/K0_med_IQR_1p5=1.068208095<br>853/0.007604523595<br>1/K0_ave_IQR_1p5=1.0632094012<br>38
Diisooctyl azelate	timsTOF	B	APCI	N2	18	[M+H]+	412.3553	methanol/water/<br>formic acid<br>(98/2/0.1)	Mods=0 Parent=413.3618<br>Mz_diff=-0.0007 Mz_diff_ppm=-1.7<br>Mz_exact=413.3625 msn=2<br>Nreps=15/15 Npksused=42<br>Parent_med=413.3619/0.0004<br>Max2med_orig=175.50/64.09<br>Logabmax=6.52/0.35<br>RT=19.070/10.021<br>ITot=7673822/3835138<br>CCS_med_IQR_1p5=221.18/1.93<br>CCS_ave_IQR_1p5=220.82

									1/K0_med_IQR_1p5=1.068909867104/0.009329711674 1/K0_ave_IQR_1p5=1.067169134039
Diisooctyl phthalate	timsTOF	B	APCI	N2	15	[M+H]+	390.277	methanol/water/ formic acid (98/2/0.1)	Mods=0 Parent=391.2822 Mz_diff=-0.0021 Mz_diff_ppm=-5.4 Mz_exact=391.2843 msn=2 Nreps=7/7 Npksused=32 Parent_med=391.2823/0.0002 Max2med_orig=172.10/44.57 Logabmax=6.68/0.25 RT=21.080/10.704 ITot=15739162/3647965 CCS_med_IQR_1p5=212.03/0.11 CCS_ave_IQR_1p5=212.08 1/K0_med_IQR_1p5=1.022840927927/0.000557456495 1/K0_ave_IQR_1p5=1.023090763412
Dimethyl 2-methylidenebutanedioate	HCD	A	ESI	N2	9 NCE=30 %	[M+H]+	158.0579	water/acetonitrile/ formic acid (50/50/0.1)	Spec= Nreps=11/30 Mz_diff=-3.8ppm
Dimethyl azelate	timsTOF	B	APCI	N2	18	[M+H]+	216.1362	methanol/water/ formic acid (98/2/0.1)	Mods=0 Parent=217.1426 Mz_diff=-0.0008 Mz_diff_ppm=-3.7 Mz_exact=217.1434 msn=2 Nreps=8/8 Npksused=58 Parent_med=217.1427/0.0002 Max2med_orig=183.80/56.29 Logabmax=6.79/0.30 RT=20.950/8.332 ITot=21262153/5889624 CCS_med_IQR_1p5=144.28/1.74 CCS_ave_IQR_1p5=145.96 1/K0_med_IQR_1p5=0.678118939245/0.008180244832 1/K0_ave_IQR_1p5=0.685979683552
Dimethyl isophthalate	timsTOF	B	APCI	N2	15	[M+H]+	194.0579	methanol/water/ formic acid (98/2/0.1)	Mods=0 Parent=195.0651 Mz_diff=-0.0001 Mz_diff_ppm=-0.5 Mz_exact=195.0652 msn=2 Nreps=7/10 Npksused=38/39 Parent_med=195.0652/0.0002 Max2med_orig=92.10/35.57 Logabmax=6.10/0.28

									RT=21.550/10.877<br>ITot=5875893/2258322<br>CCS_med_IQR_1p5=139.58/0.34<br>CCS_ave_IQR_1p5=139.58<br>1/K0_med_IQR_1p5=0.651825145866/0.001601516536<br>1/K0_ave_IQR_1p5=0.651812352496
Dimethyl phthalate	timsTOF	B	APCI	N2	30	[M+H]+	194.0579	methanol/water/formic acid (98/2/0.1)	Mods=0 Parent=195.0645<br>Mz_diff=-0.0007 Mz_diff_ppm=-3.6<br>Mz_exact=195.0652 msn=2<br>Nreps=21/21 Npksused=29<br>Parent_med=195.0640/0.0006<br>Max2med_orig=109.20/23.84<br>Logabmax=6.62/0.04<br>RT=34.270/8.260<br>ITot=14271013/1214695<br>CCS_med_IQR_1p5=131.17/0.45<br>CCS_ave_IQR_1p5=130.91<br>1/K0_med_IQR_1p5=0.612522043207/0.002081484966<br>1/K0_ave_IQR_1p5=0.611326573437
Dimethyl sebacate	timsTOF	B	APCI	N2	20	[M+H]+	230.1518	methanol/water/formic acid (98/2/0.1)	Mods=0 Parent=231.1569<br>Mz_diff=-0.0022 Mz_diff_ppm=-9.5<br>Mz_exact=231.1591 msn=2<br>Nreps=7/7 Npksused=83<br>Parent_med=231.1571/0.0003<br>Max2med_orig=188.20/54.91<br>Logabmax=6.73/0.27<br>RT=16.700/8.511<br>ITot=23431508/6060413<br>CCS_med_IQR_1p5=147.49/0.15<br>CCS_ave_IQR_1p5=147.56<br>1/K0_med_IQR_1p5=0.695605175937/0.000716736128<br>1/K0_ave_IQR_1p5=0.695943472142
Di-n-butyl maleate	timsTOF	B	APCI	N2	10	[M+H]+	228.1362	methanol/water/formic acid (98/2/0.1)	Mods=0 Parent=229.1430<br>Mz_diff=-0.0004 Mz_diff_ppm=-1.7<br>Mz_exact=229.1434 msn=2<br>Nreps=2/15 Npksused=5/7<br>Parent_med=229.1430/0.0000<br>Max2med_orig=3.25/0.65<br>Logabmax=5.23/0.10

									RT=26.900/0.210<br>ITot=462390/13470<br>CCS_med_IQR_1p5=140.32/0.00<br>CCS_ave_IQR_1p5=140.32<br>1/K0_med_IQR_1p5=0.661471853303/0.000000000000<br>1/K0_ave_IQR_1p5=0.661471853303
Di-n-butyl phthalate	timsTOF	B	APCI	N2	5	[M+H]+	278.1518	methanol/water/formic acid (98/2/0.1)	Mods=0 Parent=279.1592<br>Mz_diff=0.0001 Mz_diff_ppm=0.4<br>Mz_exact=279.1591 msn=2<br>Nreps=9/9 Npksused=11/12<br>Parent_med=279.1589/0.0003<br>Max2med_orig=91.70/42.83<br>Logabmax=4.82/0.08<br>RT=17.400/8.877<br>ITot=201128/33223<br>CCS_med_IQR_1p5=169.41/1.21<br>CCS_ave_IQR_1p5=168.42<br>1/K0_med_IQR_1p5=0.806520352955/0.005779386577<br>1/K0_ave_IQR_1p5=0.801780405188
Di-n-hexyl azelate	timsTOF	B	APCI	N2	18	[M+H]+	356.2927	methanol/water/formic acid (98/2/0.1)	Mods=0 Parent=357.2990<br>Mz_diff=-0.0009 Mz_diff_ppm=-2.5<br>Mz_exact=357.2999 msn=2<br>Nreps=12/12 Npksused=43<br>Parent_med=357.2989/0.0005<br>Max2med_orig=261.00/72.12<br>Logabmax=6.82/0.20<br>RT=17.280/9.837<br>ITot=12073688/4026986<br>CCS_med_IQR_1p5=203.96/1.48<br>CCS_ave_IQR_1p5=203.11<br>1/K0_med_IQR_1p5=0.980806522059/0.007112363868<br>1/K0_ave_IQR_1p5=0.976739476723
Diphenyl isophthalate	timsTOF	B	APCI	N2	30	[M+H]+	318.0892	methanol/water/formic acid (98/2/0.1)	Mods=0 Parent=319.0964<br>Mz_diff=-0.0001 Mz_diff_ppm=-0.3<br>Mz_exact=319.0965 msn=2<br>Nreps=14/14 Npksused=25<br>Parent_med=319.0965/0.0006<br>Max2med_orig=120.40/168.30<br>Logabmax=5.96/0.67

									RT=20.670/9.475 ITot=1598353/3246808 CCS_med_IQR_1p5=180.33/5.67 CCS_ave_IQR_1p5=185.54 1/K0_med_IQR_1p5=0.863449456237/0.027142733727 1/K0_ave_IQR_1p5=0.888378430593
Dipropylene glycol dibenzoate	timsTOF	B	APCI	N2	18	[M+H-C7H6O2]+	284.1049	methanol/water/formic acid (98/2/0.1)	Mods=0 Parent=163.0752 Mz_diff=-0.0002 Mz_diff_ppm=-1.2 Mz_exact=163.0754 msn=2 Nreps=14/14 Npksused=11 Parent_med=163.0754/0.0002 Max2med_orig=141.15/62.84 Logabmax=6.28/0.20 RT=26.990/11.586 ITot=2407356/783270 CCS_med_IQR_1p5=127.46/3.16 CCS_ave_IQR_1p5=127.60 1/K0_med_IQR_1p5=0.588019290665/0.014570467503 1/K0_ave_IQR_1p5=0.588651848953
Ditridecyl phthalate	timsTOF	B	APCI	N2	20	[M+H]+	530.4335	methanol/water/formic acid (98/2/0.1)	Mods=0 Parent=531.4352 Mz_diff=-0.0056 Mz_diff_ppm=-10.5 Mz_exact=531.4408 msn=2 Nreps=20/21 Npksused=48/50 Parent_med=531.4357/0.0009 Max2med_orig=47.55/20.32 Logabmax=6.03/0.36 RT=22.785/10.719 ITot=7026752/4149103 CCS_med_IQR_1p5=248.75/1.50 CCS_ave_IQR_1p5=249.46 1/K0_med_IQR_1p5=1.210702286464/0.007314066042 1/K0_ave_IQR_1p5=1.214158546969
Diundecyl phthalate	timsTOF	B	APCI	N2	15	[M+H]+	474.3709	methanol/water/formic acid (98/2/0.1)	Mods=0 Parent=475.3740 Mz_diff=-0.0042 Mz_diff_ppm=-8.8 Mz_exact=475.3782 msn=2 Nreps=15/15 Npksused=24 Parent_med=475.3744/0.0008 Max2med_orig=133.90/147.91 Logabmax=6.44/0.47

									RT=14.860/9.887 ITot=4502432/6348576 CCS_med_IQR_1p5=238.60/4.61 CCS_ave_IQR_1p5=237.56 1/K0_med_IQR_1p5=1.157894215287/0.022370847923 1/K0_ave_IQR_1p5=1.152851117819
Glycerol monooleate	timsTOF	B	APCI	N2	5	[M+H]+	356.2927	methanol/water/formic acid (98/2/0.1)	Mods=0 Parent=357.2995 Mz_diff=-0.0004 Mz_diff_ppm=-1.1 Mz_exact=357.2999 msn=2 Nreps=2/20 Npksused=8/9 Parent_med=357.2995/0.0005 Max2med_orig=24.75/6.25 Logabmax=5.04/0.26 RT=5.935/3.125 ITot=169790/88926 CCS_med_IQR_1p5=202.95/0.89 CCS_ave_IQR_1p5=202.95 1/K0_med_IQR_1p5=0.975964619099/0.004305536736 1/K0_ave_IQR_1p5=0.975964619099
Glycerol monostearate	timsTOF	B	APCI	N2	15	[M+H]+	358.3083	methanol/water/formic acid (98/2/0.1)	Mods=0 Parent=359.3117 Mz_diff=-0.0039 Mz_diff_ppm=-10.9 Mz_exact=359.3156 msn=2 Nreps=12/12 Npksused=51/53 Parent_med=359.3117/0.0002 Max2med_orig=41.95/17.50 Logabmax=5.06/0.21 RT=20.035/8.027 ITot=378153/227608 CCS_med_IQR_1p5=205.10/1.94 CCS_ave_IQR_1p5=203.78 1/K0_med_IQR_1p5=0.986480432562/0.009345293492 1/K0_ave_IQR_1p5=0.980146421884
Glycerol trioleate	timsTOF	B	APCI	N2	5	[M+H-C18H34O]+	884.7833	methanol/water/formic acid (98/2/0.1)	Mods=0 Parent=619.5292 Mz_diff=-0.0004 Mz_diff_ppm=-0.6 Mz_exact=619.5296 msn=2 Nreps=4/6 Npksused=4/9 Parent_med=619.5293/0.0002 Max2med_orig=8.20/2.12 Logabmax=4.05/0.15

									RT=32.750/8.440 ITot=18804/6775 CCS_med_IQR_1p5=262.27/2.72 CCS_ave_IQR_1p5=262.27 1/K0_med_IQR_1p5=1.281093488 107/0.013268326372 1/K0_ave_IQR_1p5=1.2810934881 07
Glyceryl triacetate	timsTOF	B	APCI	N2	5	[M+NH4]+	218.079	methanol/water/ formic acid (98/2/0.1)	Mods=0 Parent=236.1133 Mz_exact=236.11286 Mz_diff=0.0004 Mz_diff_ppm=1.7 msn=2 Nreps=2/2 Npksused=7/9 Parent_med=236.1133/0.0000 Max2med_orig=37.70/4.60 Logabmax=5.42/0.01 RT=19.960/0.210 ITot=498706/2868 CCS_med_IQR_1p5=146.73/0.00 CCS_ave_IQR_1p5=146.73 1/K0_med_IQR_1p5=0.692820821 353/0.000000000000 1/K0_ave_IQR_1p5=0.6928208213 53
Glyceryl tri-acetyl ricinoleate	timsTOF	B	APCI	N2	5	[M+H-C18H32 O2]+	1058.8	methanol/water/ formic acid (98/2/0.1)	Mods=0 Parent=779.5660 Mz_diff=-0.0008 Mz_diff_ppm=-1.0 Mz_exact=779.5668 msn=2 Nreps=10/11 Npksused=11/46 Parent_med=779.5663/0.0008 Max2med_orig=43.05/19.60 Logabmax=4.22/0.22 RT=13.680/8.745 ITot=53254/16561 CCS_med_IQR_1p5=284.87/0.31 CCS_ave_IQR_1p5=285.02 1/K0_med_IQR_1p5=1.397693006 351/0.001513937754 1/K0_ave_IQR_1p5=1.3984191460 75
Isodecyl diphenyl phosphate	timsTOF	B	APCI	N2	15	[M+H]+	390.196	methanol/water/ formic acid (98/2/0.1)	Mods=0 Parent=391.2021 Mz_diff=-0.0012 Mz_diff_ppm=-3.1 Mz_exact=391.2033 msn=2 Nreps=9/9 Npksused=12 Parent_med=391.2020/0.0003 Max2med_orig=182.00/118.24 Logabmax=6.51/0.26 RT=22.280/12.249

									ITot=3963996/1446156 CCS_med_IQR_1p5=199.98/0.48 CCS_ave_IQR_1p5=200.22 1/K0_med_IQR_1p5=0.964702892524/0.002328987018 1/K0_ave_IQR_1p5=0.965867223532
Isopropyl isostearate	timsTOF	B	APCI	N2	5	[M+H]+	326.3185	methanol/water/ formic acid (98/2/0.1)	Mods=0 Parent=327.3246 Mz_diff=-0.0012 Mz_diff_ppm=-3.7 Mz_exact=327.3258 msn=2 Nreps=9/9 Npksused=5/6 Parent_med=327.3247/0.0007 Max2med_orig=6.80/2.96 Logabmax=4.48/0.08 RT=20.140/5.799 ITot=34727/5824 CCS_med_IQR_1p5=204.51/1.08 CCS_ave_IQR_1p5=204.62 1/K0_med_IQR_1p5=0.980199944533/0.005172582390 1/K0_ave_IQR_1p5=0.980729317397
Isopropyl myristate	timsTOF	B	APCI	N2	5	[M+H-C3H8O]+	270.2559	methanol/water/ formic acid (98/2/0.1)	Mods=0 Parent=211.2054 Mz_diff=-0.0002 Mz_diff_ppm=-0.9 Mz_exact=211.2056 msn=2 Nreps=20/20 Npksused=10/14 Parent_med=211.2056/0.0004 Max2med_orig=19.45/3.85 Logabmax=4.46/0.07 RT=19.905/7.157 ITot=42192/8419 CCS_med_IQR_1p5=158.72/0.54 CCS_ave_IQR_1p5=158.72 1/K0_med_IQR_1p5=0.744756200282/0.002553398552 1/K0_ave_IQR_1p5=0.744745340980
Isopropyl palmitate	timsTOF	B	APCI	N2	5	[M+H-C3H8O]+	298.2872	methanol/water/ formic acid (98/2/0.1)	Mods=0 Parent=239.2371 Mz_diff=0.0002 Mz_diff_ppm=0.8 Mz_exact=239.2369 msn=2 Nreps=24/24 Npksused=10/22 Parent_med=239.2371/0.0005 Max2med_orig=17.25/4.19 Logabmax=4.73/0.13 RT=15.325/6.452 ITot=77646/19449 CCS_med_IQR_1p5=174.52/0.54

									CCS_ave_IQR_1p5=174.59 1/K0_med_IQR_1p5=0.824598996 020/0.002564994720 1/K0_ave_IQR_1p5=0.8249148759 13
Methyl acetyl ricinoleate	timsTOF	B	APCI	N2	5	[M+H-C3H8O3]+	354.277	methanol/water/ formic acid (98/2/0.1)	Mods=0 Parent=263.2363 Mz_diff=-0.0006 Mz_diff_ppm=-2.3 Mz_exact=263.2369 msn=2 Nreps=32/34 Npksused=17/23 Parent_med=263.2364/0.0004 Max2med_orig=551.20/618.81 Logabmax=5.68/0.29 RT=24.465/10.316 ITot=628175/406125 CCS_med_IQR_1p5=172.02/5.08 CCS_ave_IQR_1p5=172.52 1/K0_med_IQR_1p5=0.816705065 536/0.024121294050 1/K0_ave_IQR_1p5=0.8190413311 59 annotation_threshold=20ppm
Methyl laurate	timsTOF	B	APCI	N2	30	[M+H]+	214.1933	methanol/water/ formic acid (98/2/0.1)	Mods=0 Parent=215.2004 Mz_diff=-0.0002 Mz_diff_ppm=-0.9 Mz_exact=215.2006 msn=2 Nreps=9/9 Npksused=76/77 Parent_med=215.2006/0.0003 Max2med_orig=34.60/18.59 Logabmax=4.82/0.23 RT=15.530/10.422 ITot=335314/187462 CCS_med_IQR_1p5=164.16/0.82 CCS_ave_IQR_1p5=163.74 1/K0_med_IQR_1p5=0.771139877 648/0.003827478280 1/K0_ave_IQR_1p5=0.7691718765 57
Methyl linoleate	timsTOF	B	APCI	N2	5	[M+H]+	294.2559	methanol/water/ formic acid (98/2/0.1)	Mods=0 Parent=295.2626 Mz_diff=-0.0006 Mz_diff_ppm=-2.0 Mz_exact=295.2632 msn=2 Nreps=11/11 Npksused=10/13 Parent_med=295.2627/0.0005 Max2med_orig=29.90/8.41 Logabmax=4.97/0.25 RT=20.500/9.538 ITot=142477/47569 CCS_med_IQR_1p5=184.42/2.02

									CCS_ave_IQR_1p5=183.16 1/K0_med_IQR_1p5=0.880164675 757/0.009632995840 1/K0_ave_IQR_1p5=0.8741503697 40
Methyl linoleate	HCD	A	ESI	N2	8 NCE=1 5 % 8	[M+H]+	294.2559	water/acetonitril e/ formic acid (50/50/0.1)	Nreps=28/28; Mz_diff=-5.1ppm
Methyl oleate	timsTOF	B	APCI	N2	5	[M+H]+	296.2715	methanol/water/ formic acid (98/2/0.1)	Mods=0 Parent=297.2792 Mz_diff=0.0004 Mz_diff_ppm=1.3 Mz_exact=297.2788 msn=2 Nreps=4/7 Npksused=6/11 Parent_med=297.2792/0.0001 Max2med_orig=19.55/5.03 Logabmax=5.07/0.33 RT=28.590/5.345 ITot=165844/92357 CCS_med_IQR_1p5=193.40/0.19 CCS_ave_IQR_1p5=193.40 1/K0_med_IQR_1p5=0.923283309 141/0.000895811975 1/K0_ave_IQR_1p5=0.9232833091 41
Methyl palmitate	timsTOF	B	APCI	N2	30	[M+H]+	270.2559	methanol/water/ formic acid (98/2/0.1)	Mods=0 Parent=271.2629 Mz_diff=-0.0003 Mz_diff_ppm=-1.1 Mz_exact=271.2632 msn=2 Nreps=13/13 Npksused=66/79 Parent_med=271.2627/0.0003 Max2med_orig=17.60/11.95 Logabmax=4.88/0.21 RT=21.610/9.752 ITot=477729/291365 CCS_med_IQR_1p5=186.50/2.16 CCS_ave_IQR_1p5=184.91 1/K0_med_IQR_1p5=0.886675668 542/0.010266843203 1/K0_ave_IQR_1p5=0.8791222333 60
Methyl ricinoleate	timsTOF	B	APCI	N2	5	[M+H-H2O]+	312.2664	methanol/water/ formic acid (98/2/0.1)	Mods=0 Parent=295.2625 Mz_diff=-0.0007 Mz_diff_ppm=-2.4 Mz_exact=295.2632 msn=2 Nreps=14/14 Npksused=23 Parent_med=295.2623/0.0005 Max2med_orig=321.95/139.96

									Logabmax=6.21/0.36 RT=21.125/10.601 ITot=2096252/1534728 CCS_med_IQR_1p5=185.53/4.16 CCS_ave_IQR_1p5=184.33 1/K0_med_IQR_1p5=0.885432082028/0.019867318632 1/K0_ave_IQR_1p5=0.879741299244
Methyl stearate	timsTOF	B	APCI	N2	30	[M+H]+	298.2872	methanol/water/ formic acid (98/2/0.1)	Mods=0 Parent=299.2915 Mz_diff=-0.0030 Mz_diff_ppm=-10.0 Mz_exact=299.2945 msn=2 Nreps=13/14 Npksused=73/86 Parent_med=299.2915/0.0003 Max2med_orig=19.30/6.08 Logabmax=4.55/0.28 RT=22.430/11.297 ITot=254824/209123 CCS_med_IQR_1p5=191.47/1.24 CCS_ave_IQR_1p5=190.89 1/K0_med_IQR_1p5=0.914343467736/0.005920963217 1/K0_ave_IQR_1p5=0.911544875951
Mono-2-ethylhexyl phthalate	Q-TOF	C	APCI	N2	5	[M+H]+	278.1518	methanol/water/ formic acid (98/2/0.1)	Mz_diff=-5.0ppm
Myristic acid ethyl ester	HCD	E	ESI	N2	10 NCE=20 %	[M+H]+	256.2402	Acetonitrile/Water/ formic acid	Mz_diff=0 Nreps=14/14
n-Butyl acetyl ricinoleate	timsTOF	B	APCI	N2	15	[M+H]+	396.324	methanol/water/ formic acid (98/2/0.1)	Mods=0 Parent=397.3273 Mz_diff=-0.0039 Mz_diff_ppm=-9.8 Mz_exact=397.3312 msn=2 Nreps=2/12 Npksused=10/36 Parent_med=397.3273/0.0003 Max2med_orig=10.45/4.25 Logabmax=4.88/0.18 RT=32.060/1.250 ITot=342728/147776 CCS_med_IQR_1p5=210.63/4.70 CCS_ave_IQR_1p5=210.63 1/K0_med_IQR_1p5=1.016638027909/0.022718185983 1/K0_ave_IQR_1p5=1.016638027909

n-Butyl stearate	timsTOF	B	APCI	N2	5	[M+H]+	340.3341	methanol/water/ formic acid (98/2/0.1)	Mods=0 Parent=341.3405 Mz_diff=-0.0009 Mz_diff_ppm=-2.6 Mz_exact=341.3414 msn=2 Nreps=13/14 Npksused=9/10 Parent_med=341.3403/0.0004 Max2med_orig=44.50/21.17 Logabmax=5.14/0.26 RT=22.890/9.889 ITot=237230/135537 CCS_med_IQR_1p5=208.60/1.84 CCS_ave_IQR_1p5=207.79 1/K0_med_IQR_1p5=1.001407889 323/0.008833081585 1/K0_ave_IQR_1p5=0.9975323492 16
N-Ethyl o,p-toluenesulfonamide	timsTOF	B	APCI	N2	15	[M+H]+	199.0667	methanol/water/ formic acid (98/2/0.1)	Mods=0 Parent=200.0724 Mz_diff=-0.0016 Mz_diff_ppm=-8.0 Mz_exact=200.0740 msn=2 Nreps=7/7 Npksused=24 Parent_med=200.0724/0.0001 Max2med_orig=271.60/85.49 Logabmax=6.85/0.08 RT=21.510/10.716 ITot=16359732/3925557 CCS_med_IQR_1p5=138.74/0.09 CCS_ave_IQR_1p5=138.70 1/K0_med_IQR_1p5=0.648906103 744/0.000420403081 1/K0_ave_IQR_1p5=0.6487244508 27
N-Propyl oleate	timsTOF	B	APCI	N2	5	[M+H]+	324.3028	methanol/water/ formic acid (98/2/0.1)	Mods=0 Parent=325.3107 Mz_diff=0.0006 Mz_diff_ppm=1.8 Mz_exact=325.3101 msn=2 Nreps=17/17 Npksused=9/11 Parent_med=325.3108/0.0003 Max2med_orig=101.10/70.15 Logabmax=5.19/0.39 RT=24.990/9.960 ITot=184636/228321 CCS_med_IQR_1p5=202.05/3.66 CCS_ave_IQR_1p5=202.70 1/K0_med_IQR_1p5=0.968190657 803/0.017549467785 1/K0_ave_IQR_1p5=0.9713061708 81

o,p-Toluenesulfonamide	timsTOF	B	APCI	N2	5	[M+H]+	171.0354	methanol/water/formic acid (98/2/0.1)	Mods=0 Parent=172.0417 Mz_diff=-0.0010 Mz_diff_ppm=-5.8 Mz_exact=172.0427 msn=2 Nreps=8/8 Npksused=5/6 Parent_med=172.0419/0.0005 Max2med_orig=9.10/3.30 Logabmax=5.38/0.08 RT=26.375/4.739 ITot=339628/76890 CCS_med_IQR_1p5=140.55/0.76 CCS_ave_IQR_1p5=141.08 1/K0_med_IQR_1p5=0.650905288629/0.003526013974 1/K0_ave_IQR_1p5=0.653362841923
Oleic acid ethyl ester	HCD	E	ESI	N2	18 NCE=30 %	[M+H]+	310.2872	Acetonitrile/Water/ formic acid	Mz_diff=-0.0004; Nreps=15/15
Oleic acid methyl ester	HCD	A	ESI	N2	17 NCE=30 %	[M+H]+	296.2715	micromol/L in water/acetonitrile/ formic acid (50/50/0.1)	Nreps=26/26; Mz_diff=0.7ppm;
Palmitic acid ethyl ester	HCD	E	ESI	N2	17 NCE=30 %	[M+H]+	284.2715	Acetonitrile/Water/ formic acid	Mz_diff=0.0002 Nreps=15/15
Polyethylene glycol dibenzoate	timsTOF	B	APCI	N2	30	[M+H-C7H6O2]+	270.0892	methanol/water/formic acid (98/2/0.1)	Mods=0 Parent=149.0602 Mz_diff=0.0005 Mz_diff_ppm=3.4 Mz_exact=149.0597 msn=2 Nreps=9/9 Npksused=10 Parent_med=149.0601/0.0001 Max2med_orig=27.90/6.86 Logabmax=5.92/0.16 RT=21.880/11.131 ITot=1891760/785806 CCS_med_IQR_1p5=120.69/0.19 CCS_ave_IQR_1p5=120.79 1/K0_med_IQR_1p5=0.552969690527/0.000877290528 1/K0_ave_IQR_1p5=0.553447773581
Propylene glycol monostearate	timsTOF	B	APCI	N2	5	[M+H-C3H8O]+	342.3134	methanol/water/formic acid (98/2/0.1)	Mods=0 Parent=283.2628 Mz_diff=-0.0004 Mz_diff_ppm=-1.4 Mz_exact=283.2632 msn=2 Nreps=17/18 Npksused=2/4 Parent_med=283.2627/0.0008

									Max2med_orig=5.70/2.54 Logabmax=4.65/0.09 RT=19.760/4.924 ITot=52987/10676 CCS_med_IQR_1p5=187.19/0.15 CCS_ave_IQR_1p5=187.20 1/K0_med_IQR_1p5=0.891743580353/0.000706307611 1/K0_ave_IQR_1p5=0.891775253678
Propylene glycol ricinoleate	timsTOF	B	APCI	N2	5	[M+H-H2O]+	356.2927	methanol/water/formic acid (98/2/0.1)	Mods=0 Parent=339.2886 Mz_diff=-0.0008 Mz_diff_ppm=-2.4 Mz_exact=339.2894 msn=2 Nreps=24/24 Npksused=13/14 Parent_med=339.2885/0.0005 Max2med_orig=173.20/134.55 Logabmax=6.36/0.28 RT=21.435/10.981 ITot=2875544/1470323 CCS_med_IQR_1p5=192.16/3.82 CCS_ave_IQR_1p5=193.32 1/K0_med_IQR_1p5=0.922292803117/0.018321704457 1/K0_ave_IQR_1p5=0.927855093849
Ricinoleic acid	HCD	A	ESI	N2	26 NCE=85 %	[M-H]-	298.2508	Acetonitrile/Water/ formic acid	Mz_diff=-0.0001
Ricinoleic acid methyl ester	HCD	A	ESI	N2	12 NCE=20 %	[M+H]+	312.2664	Acetonitrile/Water/ formic acid	Mz_diff=-0.0003 Nreps=7/7
Stearic acid ethyl ester	HCD	E	ESI	N2	12 NCE=20 %	[M+H]+	312.3028	Acetonitrile/Water/ formic acid	Mz_diff=0.0004 Nreps=4/4
t-Butylphenyl diphenyl phosphate	timsTOF	B	APCI	N2	30	[M+H]+	382.1334	methanol/water/formic acid (98/2/0.1)	Mods=0 Parent=383.1382 Mz_diff=-0.0025 Mz_diff_ppm=-6.5 Mz_exact=383.1407 msn=2 Nreps=14/15 Npksused=29/30 Parent_med=383.1395/0.0017 Max2med_orig=214.95/353.84 Logabmax=5.68/0.24 RT=17.685/10.427 ITot=822964/321413 CCS_med_IQR_1p5=196.05/2.56 CCS_ave_IQR_1p5=197.95

									1/K0_med_IQR_1p5=0.945097107878/0.012327770295 1/K0_ave_IQR_1p5=0.954254515768
Tetrahydrofurfuryl oleate	timsTOF	B	APCI	N2	20	[M+H]+	366.3134	methanol/water/ formic acid (98/2/0.1)	Mods=0 Parent=367.3203 Mz_diff=-0.0004 Mz_diff_ppm=-1.1 Mz_exact=367.3207 msn=2 Nreps=12/21 Npksused=79/82 Parent_med=367.3203/0.0005 Max2med_orig=61.80/24.48 Logabmax=5.88/0.35 RT=13.530/10.865 ITot=3132692/2299397 CCS_med_IQR_1p5=206.81/2.47 CCS_ave_IQR_1p5=206.48 1/K0_med_IQR_1p5=0.995503396211/0.011897487067 1/K0_ave_IQR_1p5=0.993908062491
Tri(n-octyl,n-decyl) trimellitate	timsTOF	B	APCI	N2	5	[M+H-C36H74O]+	966.8615	methanol/water/ formic acid (98/2/0.1)	Mods=0 Parent=445.2967 Mz_diff=0.0018 Mz_diff_ppm=4.0 Mz_exact=445.2949 msn=2 Nreps=2/2 Npksused=5/10 Parent_med=445.2967/0.0000 Max2med_orig=9.85/1.05 Logabmax=4.56/0.10 RT=17.895/0.205 ITot=102450/2432 CCS_med_IQR_1p5=227.38/0.00 CCS_ave_IQR_1p5=227.38 1/K0_med_IQR_1p5=1.101377224892/0.000000000000 1/K0_ave_IQR_1p5=1.101377224892
Tri-butoxyethyl phosphate	timsTOF	B	APCI	N2	20	[M+H]+	398.2433	methanol/water/ formic acid (98/2/0.1)	Mods=0 Parent=399.2476 Mz_diff=-0.0030 Mz_diff_ppm=-7.5 Mz_exact=399.2506 msn=2 Nreps=15/15 Npksused=42 Parent_med=399.2481/0.0016 Max2med_orig=112.30/142.06 Logabmax=6.41/0.33 RT=19.390/9.424 ITot=9000767/3202774 CCS_med_IQR_1p5=196.80/1.90 CCS_ave_IQR_1p5=197.19

									1/K0_med_IQR_1p5=0.950018081698/0.009179125541<br>1/K0_ave_IQR_1p5=0.951929920095
Tributyl phosphate	timsTOF	B	APCI	N2	10	[M+H]+	266.1647	methanol/water/formic acid (98/2/0.1)	Mods=0 Parent=267.1717<br>Mz_diff=-0.0003 Mz_diff_ppm=-1.1<br>Mz_exact=267.1720 msn=2<br>Nreps=7/7 Npksused=20<br>Parent_med=267.1717/0.0002<br>Max2med_orig=182.30/81.14<br>Logabmax=6.29/0.10<br>RT=19.360/9.580<br>ITot=6505526/1734922<br>CCS_med_IQR_1p5=165.89/0.29<br>CCS_ave_IQR_1p5=165.88<br>1/K0_med_IQR_1p5=0.788122535431/0.001357432905<br>1/K0_ave_IQR_1p5=0.788080136644
Tricapryl trimellitate	timsTOF	B	APCI	N2	10	[M+H]+	546.392	methanol/water/formic acid (98/2/0.1)	Mods=0 Parent=547.3980<br>Mz_diff=-0.0013 Mz_diff_ppm=-2.4<br>Mz_exact=547.3993 msn=2<br>Nreps=2/15 Npksused=5/10<br>Parent_med=547.3980/0.0002<br>Max2med_orig=7.50/4.00<br>Logabmax=5.43/0.08<br>RT=15.620/6.600<br>ITot=727689/192800<br>CCS_med_IQR_1p5=246.77/0.10<br>CCS_ave_IQR_1p5=246.77<br>1/K0_med_IQR_1p5=1.201938552842/0.000491050632<br>1/K0_ave_IQR_1p5=1.201938552842
Tricresyl phosphate	timsTOF	B	APCI	N2	30	[M+H]+	368.1177	methanol/water/formic acid (98/2/0.1)	Mods=0 Parent=369.1240<br>Mz_diff=-0.0010 Mz_diff_ppm=-2.7<br>Mz_exact=369.1250 msn=2<br>Nreps=7/10 Npksused=74/80<br>Parent_med=369.1239/0.0006<br>Max2med_orig=18.30/26.39<br>Logabmax=5.16/0.17<br>RT=5.110/8.686<br>ITot=699096/162319<br>CCS_med_IQR_1p5=186.21/0.24<br>CCS_ave_IQR_1p5=186.36

									1/K0_med_IQR_1p5=0.896525926<br>828/0.001178532166<br>1/K0_ave_IQR_1p5=0.8972190521<br>20
Triethyl citrate	timsTOF	B	APCI	N2	10	[M+H]+	276.1209	methanol/water/<br>formic acid<br>(98/2/0.1)	Mods=0 Parent=277.1283<br>Mz_diff=0.0001 Mz_diff_ppm=0.4<br>Mz_exact=277.1282 msn=2<br>Nreps=7/7 Npksused=26<br>Parent_med=277.1283/0.0004<br>Max2med_orig=851.70/146.43<br>Logabmax=7.11/0.08<br>RT=22.560/10.727<br>ITot=21854556/3463858<br>CCS_med_IQR_1p5=158.50/0.20<br>CCS_ave_IQR_1p5=158.38<br>1/K0_med_IQR_1p5=0.754296676<br>084/0.000943198339<br>1/K0_ave_IQR_1p5=0.7537654719<br>18
Triisononyl<br>trimellitate	timsTOF	B	APCI	N2	20	[M+H]+	588.439	methanol/water/<br>formic acid<br>(98/2/0.1)	Mods=0 Parent=589.4451<br>Mz_diff=-0.0012 Mz_diff_ppm=-2.0<br>Mz_exact=589.4463 msn=2<br>Nreps=14/17 Npksused=33/35<br>Parent_med=589.4451/0.0006<br>Max2med_orig=39.95/19.36<br>Logabmax=5.66/0.31<br>RT=20.385/11.275<br>ITot=1905914/1576043<br>CCS_med_IQR_1p5=262.19/1.74<br>CCS_ave_IQR_1p5=262.29<br>1/K0_med_IQR_1p5=1.279262587<br>999/0.008465493482<br>1/K0_ave_IQR_1p5=1.2797543606<br>41
Tri-n-butyl citrate	timsTOF	B	APCI	N2	10	[M+H]+	360.2148	methanol/water/<br>formic acid<br>(98/2/0.1)	Mods=0 Parent=361.2215<br>Mz_diff=-0.0006 Mz_diff_ppm=-1.7<br>Mz_exact=361.2221 msn=2<br>Nreps=12/12 Npksused=32<br>Parent_med=361.2219/0.0013<br>Max2med_orig=154.95/57.13<br>Logabmax=6.55/0.20<br>RT=20.605/9.656<br>ITot=9169828/4482074<br>CCS_med_IQR_1p5=193.03/2.15<br>CCS_ave_IQR_1p5=191.59

									1/K0_med_IQR_1p5=0.928626925474/0.010317162597 1/K0_ave_IQR_1p5=0.921671038497
Triphenyl phosphate	timsTOF	B	APCI	N2	30	[M+H]+	326.0708	methanol/water/ formic acid (98/2/0.1)	Mods=0 Parent=327.0765 Mz_diff=-0.0016 Mz_diff_ppm=-4.9 Mz_exact=327.0781 msn=2 Nreps=16/18 Npksused=53 Parent_med=327.0772/0.0013 Max2med_orig=29.20/76.81 Logabmax=5.66/0.21 RT=15.975/10.166 ITot=2416864/2238092 CCS_med_IQR_1p5=172.69/4.26 CCS_ave_IQR_1p5=175.69 1/K0_med_IQR_1p5=0.827683185740/0.020403355856 1/K0_ave_IQR_1p5=0.842067576264

IX. Mass spectrometry techniques for polymer and plasticizers analysis

GC-MS (Gas Chromatography-Mass Spectrometry): a widely used technique for plasticizer analysis, which is more standardized in the industry. Plasticizers are often volatile or can be made volatile through derivatization, allowing them to be separated by GC before entering the MS. The mass spectrum provides a unique "fingerprint" for identification by comparing it to spectral libraries (e.g., NIST library). GC-MS is excellent for both qualitative identification and quantitative analysis of volatile small-molecule plasticizers, such as phthalates (e.g., DEHP, DBP, DINP).

LC-MS (Liquid Chromatography-Mass Spectrometry): The preferred method for less volatile or thermally labile plasticizers. LC separates the plasticizers, and the eluent is then introduced into the MS for detection. This is particularly useful for analyzing mixtures of plasticizers or those with higher molecular weights.

DART-MS (Direct Analysis in Real Time Mass Spectrometry): Certain MS techniques (also, ASAP-MS (Atmospheric Solids Analysis Probe Mass Spectrometry)) enable the direct analysis of plasticizers without requiring extensive sample preparation, thereby providing rapid screening capabilities.

Migration Studies: Most plasticizers are not covalently bound to the polymer matrix and can migrate out of the material. MS can be used to study this migration into food, medical devices, or the environment, which is crucial for regulatory compliance and safety assessments.

Quality Control: MS ensures that the correct type and amount of plasticizer are present in a product, helping manufacturers maintain product quality and avoid the use of restricted or banned substances. In competitive analysis or troubleshooting, MS can help "deformulate" a polymer product to identify the exact plasticizers used in its composition.

MALDI-TOF MS (Matrix-Assisted Laser Desorption/Ionization-Time-of-Flight Mass Spectrometry): This is the go-to technique for polymer molecular weight analysis. A polymer sample is mixed with a matrix compound, co-crystallized, and then irradiated with a laser. The matrix absorbs the laser energy, transferring it to the polymer molecules, causing them to desorb and ionize. TOF analyzers are well-suited for high molecular weight species, providing information on absolute average molecular weights (Mn, Mw), dispersity (Đ), and degree of polymerization.

ESI-MS (Electrospray Ionization Mass Spectrometry): While generally better for smaller, polar molecules, ESI can be used for polymers, especially in conjunction with LC. It often produces multiply charged ions, which can be challenging to interpret for broad polymer distributions. The ends of polymer chains usually contain functional groups that determine their reactivity and properties. MS, particularly MALDI-TOF MS combined with MS/MS (tandem mass spectrometry), can precisely identify and characterize these end groups, providing insights into polymerization mechanisms and reactions. Copolymer Composition and Sequence Distribution: For copolymers (polymers made from two or more different monomers), MS can determine the precise composition of individual chains and even the sequence in which the monomers are arranged along the polymer backbone.

Additive Analysis (beyond plasticizers): Polymers contain various additives (antioxidants, UV stabilizers, flame retardants, colorants, etc.) that affect their properties and performance. MS techniques (GC-MS, LC-MS, MALDI-TOF MS) are widely used to identify and quantify these additives, often after extraction from the polymer matrix. MS is invaluable for understanding how polymers degrade due to heat, light, or environmental factors.

Pyrolysis-GC-MS (Py-GC-MS): This technique involves thermally degrading the polymer in a controlled manner, generating volatile fragments that are then separated by GC and analyzed by MS. The characteristic fragments provide information about the polymer's structure and degradation pathways.

Direct MS of Degradation Products: Some MS techniques can directly analyze degradation products, offering insights into the molecular changes occurring during aging or decomposition.

Surface Analysis and Imaging: Certain MS techniques (e.g., MALDI-MSI - Mass Spectrometry Imaging, DESI - Desorption Electrospray Ionization) can be employed to analyze the chemical composition of polymer surfaces and visualize the distribution of components (polymers, plasticizers, additives) on the surface.

X. Raman and MS spectral signatures associated with core functional groups

We identified that Raman bands associated with core functional features of plasticizer families tend to be consistent within a narrow distribution across family members. Therefore, we present these characteristic family features below, focusing on the unambiguous shared bands associated with the core functional chemistry. Distinct features of individual family members in Raman Spectroscopy tend to occur between (≈ 700 to 900) cm^{-1} associated with C-C backbone of the aliphatic substituent groups. We also consider the characteristic analytical features of each family with respect to both Raman spectroscopy (RS) and mass spectrometry (MS). Raman peak assignments were assigned based on Socrates.[4]

Aliphatic Esters: The most numerous classes of plasticizers in this study include aliphatic esters derived from mono-, di-, and tri-substituted organic acids.

<u>Monoesters</u>

Oleates (N=4)

The esters of oleic acid ($C_{18}H_{33}O_2$), it is classified as a monounsaturated omega-9 fatty acid.

RS: The primary Raman peaks associated with oleates include $(CH_2)_n$ twisting (1299 to 1303) cm^{-1}, CH_2 vibration (1438 to 1441) cm^{-1}, cis-dialkyl alkenes C=C stretching (1653 to 1657) cm^{-1}, carbonyl stretching (1735 to 1742) cm^{-1}.

MS: In tandem mass spectrometry, the fragmentation of oleates depends on the size of the alkyl chain of the alcohol. In short chains, such as those found in methyl and ethyl oleate, the most intense peaks result from the breaking of the ester bond, producing acylium ions at m/z 265 and the loss of the alcohol. The acylium ion undergoes a subsequent loss of water, producing a peak at m/z 247. If the alcohol chain is larger, the oleate usually also undergoes loss of the alkyl chain of the alcohol, producing a peak of protonated oleic acid at m/z 283. These peaks are present at very low collision energies. At higher energies, multiple peaks related to the fragmentation of the oleic acid chain appear that are common to most oleates, e.g., peaks at m/z 223, m/z 177, m/z 163, m/z 135, and many others. However, none of these peaks pinpoint directly to the position of the double, which requires special techniques if necessary.

Ricinoleates (N=7)

The esters of ricinoleic acid ($C_{18}H_{32}O_3$), it is classified as a monounsaturated omega-9 fatty acid and a hydroxy acid. It is a significant component of castor oil and a bio-derived plasticizer.

RS: The primary Raman peaks associated with ricinoleates include secondary-alcohol in-plane O-H deformation vibration (1264 to 1266) cm^{-1}, $(CH_2)_n$ twisting (1301 to 1303) cm^{-1}, CH_2 vibration (1438 to 1441) cm^{-1}, cis-dialkyl alkenes C=C stretching (1655 to 1657) cm^{-1}, carbonyl stretching (1735 to 1742) cm^{-1}.

MS: Esters of Ricinooleic acid and its ester derivatives undergo loss of 18 Da (water) from the hydroxyl group on the alkyl chain of the acid as the lowest energy pathway. Followed by the loss of the corresponding alcohol and followed for another water loss, for example, the parent ion of ricinoleic acid methyl ester shows the most intense peaks, at low collision energy, at m/z 313 (parent ion), m/z 295 (loss of water), m/z 263 (loss of alcohol + water loss), and m/z 245 (a second water loss from the ion at m/z 263).

Stearates (N=6)

The present library contains three forms of stearate compounds: stearates (N = 2), isostearates (N = 1), and monostearates (N = 3). Stearates are a class of bio-derived plasticizers, derived from the fatty acid, stearic acid, consisting of seventeen CH_2 units bonded to the carbonyl carbon of an ester ($C_{18}H_{35}O_2$). Isostearates are derived from isostearic acid, composed of fourteen CH_2 units with a terminal isopropyl group on one side and bonded to the carbonyl carbon of an ester on the other ($C_{18}H_{35}O_2$). Monostearates indicate a single stearic acid esterified to a di- or tri-acid such as glycerol, ethylene glycol, and propylene glycol.

RS: The primary Raman peaks associated with all forms of stearates include straight chain alkane C-C stretching (1062 to 1064, 1098 to 1106) cm^{-1}, $(CH_2)_n$ twisting (1294 to 1301) cm^{-1}, CH_2 vibration (1436 to 1442) cm^{-1}, carbonyl stretching (1722 to 1742) cm^{-1}. Primary distinctions between the three include differences in the observed peak range of the carbonyl stretching (stearates: (1739 to 1742) cm^{-1}, monostearates: (1722 to 1739) cm^{-1}, isostearates: only one example in the library) and the $(CH_2)_n$ twisting. Further details can be found in **Table S7.**

MS: At low collision energies, the most intense product ion of stearic acid ethyl ester is the peak of protonated stearic acid at m/z 285 due to the loss of the alkyl chain of ethene. At higher collision energies, the peak at m/z 267 due to the loss of the alcohol is observed, along with other small fragments at m/z 103, m/z 89, and m/z 85. Still, these other peaks are not particularly helpful in distinguishing between compounds with very long alkyl chains.

Other monoalkyl esters

These compounds are present in the library, including the saturated esters: laurate (N = 1, $C_{12}H_{27}O_2$), myristate (N = 1, $C_{14}H_{27}O_2$), palmitate (N = 2, $C_{16}H_{31}O_2$), and the unsaturated ester: linoleate (N = 1, $C_{12}H_{23}O_2$).

RS: The primary Raman bands shared among these samples include the straight chain alkane C-C stretching (1075 to 1098) cm^{-1}, $(CH_2)_n$ twisting (1294 to 1301) cm^{-1}, CH_2 vibration (1438 to 1440) cm^{-1}, and carbonyl stretching (1735 to 1742) cm^{-1}. The unsaturated methyl linoleate spectra also included the characteristic cis-dialkyl alkenes C=C stretching at 1655 cm^{-1}.

MS: The MS^2 spectrum of myristic acid ethyl ester shows a very intense peak at m/z 229, corresponding to the loss of ethene, and some small peaks at m/z 103, m/z 89, and m/z 85. This fragmentation pattern is similar to the corresponding ester of stearic acid. Ethyl palmitate exhibits a similar fragmentation pattern to ethyl stearate and ethyl myristate, characterized by an intense peak at m/z 257 due to the loss of ethene, as well as small peaks at m/z 103, m/z 89, and m/z 85. The methyl ester of linoleic acid, similar to many other aliphatic esters, exhibits, at low collision energy, a prominent loss of methanol peak at m/z 263 and a minor loss of water peak at m/z 277.

<u>Di-Esters</u>

Adipates (N=7)

The esters of adipic acid consisting of four CH_2 units bound by two substituted ester end groups ($C_6H_8O_4$).

RS: The primary Raman peaks associated with adipates include alkane C-C stretching (1058 to 1064) cm^{-1}, $(CH_2)_n$ twisting (1301 to 1304) cm^{-1}, CH_2 vibration (1440 to 1448) cm^{-1}, and carbonyl stretching (1733 to 1738) cm^{-1}.

MS: The fragmentation of the parent ions of adipates is characterized by major peaks, including loss of alcohol, loss of the hydrocarbon from the alcohol chain, and combinations of both losses. For example, the most intense peaks of Diisobutyl adipate appear at m/z 185, corresponding to the loss of isobutyl alcohol, and the second most intense peak appears at m/z 129, corresponding to the combined loss of alcohol and isobutylene. Based on the

trend of fragmentation with increasing collision energy, it seems that the loss of hydrocarbon follows the loss of alcohol in a consecutive reaction. This fragmentation pattern remains unchanged mainly when hydrocarbon chains are more extended. For example, Diisodecyl adipate shows intense peaks at m/z 269, loss of alcohol, and the same peak at m/z 129, corresponding to the consecutive loss of alcohol and 8-methylnon-1-ene (or a rearranged form of the hydrocarbon).

Azelates (N=5)

The esters of azelaic acid consisting of seven CH_2 units bounded by two substituted ester end groups ($C_9H_{14}O_4$).

RS: The primary Raman peaks associated with azelates include alkane C-C stretching (1064 to 1089) cm^{-1}, $(CH_2)_n$ twisting (1303 to 1304) cm^{-1}, CH_2 vibration (1440 to 1448) cm^{-1}, carbonyl stretching (1734 to 1736) cm^{-1}.

MS: The fragmentation of azelates is more complicated than that of adipates. It seems that the loss of alcohol is followed not only by the loss of the alkene but also, in a competitive pathway, by the loss of the alkylformate. For example, Dimethyl azelate shows intense peaks at m/z 185, m/z 135, and m/z 125.

Other dialkyl esters

The present library also contains several dialkyl esters that didn't have sufficient samples to identify unique characteristic peaks. These included diisobutyrates (N = 1, $(C_4H_7O_2)_2$), succinates (N = 1, $C_4H_4O_4$), sebacates (N = 3, $C_{10}H_{16}O_4$), and the unsaturated esters, fumarates (N = 1, $C_4H_2O_4$) and maleates (N = 2, $C_4H_4O_4$).

RS: The primary Raman bands shared among these samples include the $(CH_2)_n$ twisting (1298 to 1305) cm^{-1}, CH_2 vibration (1440 to 1453) cm^{-1}, and carbonyl stretching (1728 to 1735) cm^{-1}. The unsaturated fatty acids also have a cis-dialkyl alkene C=C stretch at (1645 to 1648) cm^{-1}.

MS: Most di-esters show the breaking of the ester bond as a very intense peak corresponding to the loss of the alcohol. The fragmentation that follows the ester bond breaking depends on the di-ester core structure and the length of the hydrocarbon chains. For example, the parent ion of the protonated Succinic acid, methylene-, dimethyl ester (m/z 159), undergoes loss of the alcohol, producing a peak at m/z 127, and a subsequent loss of carbon monoxide, producing a peak at m/z 99. On the other hand, Succinic acid, methylene-, dibutyl ester (m/z 243) undergoes loss of alcohol, producing a peak at m/z 169, and subsequently loses the remaining hydrocarbon chain instead of CO, producing a peak at m/z 113. Di(2-ethylhexyl) sebacate exhibits peaks at m/z 315 (loss of one of the hydrocarbon chains) and at m/z 297 (a subsequent loss of water). The most intense peak at m/z 185 corresponds to the simultaneous losses of alcohol and the remaining hydrocarbon chain. In contrast, Di(2-ethylhexyl) maleate (parent ion at m/z 341) undergoes simultaneous losses of both hydrocarbon chains, producing the most intense peak at m/z 117. Followed by a loss of water, producing a peak at m/z 99. Dibutyl fumarate (m/z 229) undergoes losses of alcohol and one of the hydrocarbon chains at about the same rate, producing peaks at m/z 155 and m/z 173. We do not have sufficient data at different collision energies to determine whether this is simply a loss of a hydrocarbon chain followed by the loss of water. However, the most intense peak appears at m/z 99, which probably originated from a loss of water from the peak at m/z 117 (the simultaneous losses of both hydrocarbon chains.

<u>Tri-Esters</u>

Citrates (N=4)

A class of biobased plasticizers derived from citric acid, which can be variously substituted to have one to three ester or ether* functional groups (C_6H_4*O_7).

RS: The primary Raman peaks associated with citrates include alcohol C-O stretching vibration, tertiary alcohol (1115 to 1122) cm^{-1}, CH_2 vibration (1450 to 1453) cm^{-1}, carbonyl stretching (1736 to 1741) cm^{-1}.

MS: The protonated Acetyl tri-n-butyl citrate (m/z 403) undergoes loss of ethenone ketene, producing a peak at m/z 361. The most intense appears at m/z 329 and corresponds to the loss of alcohol. Other important fragmentations include the loss of one of the remaining hydrocarbon chains (or alcohol), producing a peak at m/z 273 (m/z 255). This process repeats at higher energies, producing peaks at m/z 217 and m/z 259.

Glycol and glycerol esters (N=11)

Glycol and glycerol esters are multifunctional alcohols, with glycols having two hydroxy groups and glycerol having three. In the esterified form, glycerols and glycols may be functionalized with one or more carboxylic acid groups, as seen in glycerol monooleate and glycerol trioleate. In the library, some plasticizers are referred to as glyceryl, indicating a glycerol that has had one or more of its hydroxy hydrogens removed or substituted, as in glyceryl triacetate. However, the standard naming system is inconsistent, and the terms "glyceryl" and "glycerol" are often used interchangeably. In the library, the common name is presented alongside the CAS numbers and IUPAC names for clarity.

RS: The Raman bands present in these compounds are generally in agreement with their respective carboxylic acid functional groups and can be referenced with the above sections. The expected bands associated with the ether and alcohol bonds tend to sit in the range (800 to 900) cm^{-1} overlapping with aliphatic C-C bonds.

MS: Glycerol and glycol esters undergo loss of water relatively easily, which is not particularly informative. Glycerol esters undergo separate losses of the alcohol and the alcohol hydrocarbon chain. For example, protonated glycerol monooleate (m/z 357) produces peaks at m/z 339 (loss of water), m/z 283 (loss of the alcohol hydrocarbon chain), and m/z 265 (loss of alcohol). Protonated Glycerol monostearate (m/z 359) produces peaks at m/z 341 (loss of water, very intense), loss of the alcohol hydrocarbon chain (m/z 283), and loss of alcohol (m/z 265). The tandem mass spectra of Glycerol monooleate and Glycerol monostearate show more specific differences at higher energies; below m/z 180, they are very distinctive due to the presence of the double bond in the monooleate.

Aromatic Esters

Phthalates (N=16)

The esters of ortho-phthalic acid ($C_8H_4O_4$).

RS: The primary Raman peaks associated with phthalates include ring in-plane bending vibration (649 to 651) cm^{-1}, ring C-H in-plane bending vibration (1038 to 1040) cm^{-1}, ester symmetric COC stretching (1161 to 1165) cm^{-1}, ester asymmetric COC stretching (1271 to 1299) cm^{-1}, CH_2 vibration (1438 to 1451) cm^{-1}, ring stretching (1579 to 1581, 1598 to 1600) cm^{-1}, carbonyl stretching (1721 to 1728) cm^{-1}.

*The dicyclohexyl phthalate carbonyl band was exceptionally low at 1715 cm^{-1}, and the spectra had numerous sharp, medium intensity bands in the range (200 to 1430) cm^{-1} largely attributed to it being a crystalline room temperature solid. It should be noted that spectra of cyclohexyl phthalate as a room-temperature crystalline solid will likely differ from the spectral contributions of cyclohexyl phthalate as a room-temperature amorphous constituent dissolved in a solid substrate.

MS: The fragmentation of phthalates is usually very regular, starting with the loss of alcohol and then the loss of the hydrocarbon chain of the other alcohol, producing the typical peak at m/z 149. For example, protonated Diisobutyl phthalate (m/z 279) shows peaks at m/z 205 (loss of alcohol) and the peak at m/z 149. Similarly, the spectrum of protonated Didecyl phthalate shows intense peaks at m/z 289 and m/z 149. Even the spectrum of protonated Diethyl phthalate (m/z 223) shows the same pattern, peaks at m/z 177 and m/z 149. However, the spectrum of protonated Dimethyl phthalate is slightly different. At higher energies, the loss of alcohol at m/z 163 co-occurs with the loss of methyl formate, resulting in a peak at m/z 135. This is followed by the loss of formaldehyde, producing the acylium ion peak at m/z 105. There is no peak at m/z 149.

Isophthalates (N=3)

The esters of meta-phthalic acid ($C_8H_4O_4$).

RS: The primary Raman peaks associated with isophthalates include ring in-plane bending vibration (655 to 659) cm^{-1}, benzene ring vibration (1003 to 1004) cm^{-1}, ester symmetric COC stretching (1154 to 1161) cm^{-1}, ester asymmetric COC stretching (1238 to 1249) cm^{-1}, (CH2)n twisting (1301 to 1308) cm^{-1}, CH_2 vibration (1438 to 1448) cm^{-1}, Ring stretching (1587 to 1591) cm^{-1}, carbonyl stretching (1719 to 1731) cm^{-1}.

Dibenzoates (N=3)

The esters of benzoic acid ($C_7H_5O_2$) are commonly linked by glycols such as diethylene glycol, dipropylene glycol, and polyethylene glycol. Both benzoates and dibenzoates are used as plasticizers; however, the present library only contains dibenzoates.

RS: The primary Raman peaks associated with dibenzoates include the aromatic in-plane ring deformation vibration 618 cm^{-1}, out-of-plane ring deformation vibration (675 to 676) cm^{-1}, =C-H in-plane ring deformation vibration (1001 to 1002) cm^{-1}, ester asym COC stretching (1273 to 1275) cm^{-1}, CH_2 vibration (1451 to 1452) cm^{-1}, Ring stretching 1602 cm^{-1}, carbonyl stretching (1715 to 1717) cm^{-1}.

Trimellitates (N=4)

The esters of trimellitic acid (Benzene-1,2,4-tricarboxylic acid, $C_9H_3O_6$).

RS: The primary Raman peaks associated with trimelitates include ring in-plane bending vibration (665 to 667) cm^{-1}, ring C-H in-plane bending vibration (1038 to 1040) cm^{-1}, ester symmetric COC stretching (1161 to 1165) cm^{-1}, ester asymmetric COC stretching (1271 to 1299) cm^{-1}, CH_2 vibration (1438 to 1450) cm^{-1}, aromatic ring stretching vibration (1574 to 1577, 1610 to 1612) cm^{-1}, carbonyl stretching (1725 to 1730) cm^{-1}.

MS: The fragmentation pattern of protonated trimellitates is like that of phthalates and diphthalates. The fragmentation is rich due to the presence of three ester bonds, typically with voluminous hydrocarbon chains. For example, protonated Triisononyl trimellitate (m/z 589) undergoes loss of isononyl alcohol (m/z 445), loss of the isononyl chain (m/zf 463), loss of two isononyl alcohol molecules (m/z 319), and the combined losses of two alcohols, the isononyl chain from the third alcohol, and carbon monoxide (m/z 127).

Non-esters

Organophosphates (N=7)

Plasticizers formed by tri-substitution of the phosphate ion with either the same or mixed substituent groups. The present library contains five aryl phosphates and two alkyl phosphates. Aryl phosphates are notable for their enhanced flame-retardant properties and low volatility, and are often incorporated specifically for these properties.[5] The Raman spectra of the aryl and alkyl phosphates have low similarity between the two groups because the contribution of the aromatic bands in the aryl phosphates is significantly stronger than the characteristic phosphate bands. Raman bands associated with phosphate bonds are more pronounced in alkyl phosphates; however, they tend to be weak overall in Raman spectra.

RS: The Raman spectra of the aryl and alkyl phosphates have low similarity between the two groups because the contribution of the aromatic bands in the aryl phosphates is significantly stronger than the characteristic phosphate bands. Phosphate bands are more significant in the alkyl phosphates; however, these bands tend to be weak overall in Raman spectra. Common, but weak bands that are seen across all the phosphate samples include P-O-C stretching vibrations at (1025 to 1029) cm^{-1} and (1222 to 1233) cm^{-1}, P=O stretching vibration (1289 to 1301) cm^{-1}, and at (1445 to 1459) cm^{-1} associated with several alkane C-H deformation vibrations and a weak Raman band for aromatic -C=C- stretching vibration. However, it should be noted that all of these bands are very weak in both the aromatic and alkyl samples.

MS: The most intense peak of the protonated phosphates originates from the breaking of the phosphate ester bond. For example, protonated 2-Ethylhexyl diphenyl phosphate (m/z 363) shows an intense peak at m/z 251, with no other major peaks. MS^3 experiments on an Orbitrap show that the peak at m/z 251 undergoes two consecutive water losses at m/z 233 and m/z 215, and the loss of phosphoric acid (m/z 153) is the major peak.

Aromatic sulfonamides (N=2)

A highly polar class of plasticizers consisting of a sulfonamide functional group linked to an aromatic ring (C_6H_5*NO_2S). The present library contains two aromatic sulfonamides, both of which are isomeric mixtures: o-, p-toluene sulfonamide and n-ethyl o-, p-toluene sulfonamide.

RS: significant Raman bands associated with sulfonamide functional groups observed in these two aromatic sulfonamides samples included SO_2 wagging vibration (542 to 544) cm^{-1}, N-H out-of-plane bending vibration (687 to 693) cm^{-1}, C-C stretching associated with o-substituted benzene (805 to 809) cm^{-1}, SO_2 symmetric stretching vibration (1147 to 1155) cm^{-1}, and overlapping bands at (1595 to 1598) cm^{-1} accounting for aromatic ring stretching and NH scissor vibration.

Oligomeric plasticizers

MS, polymeric plasticizers: Tandem mass spectral libraries are usually restricted to small molecules. However, this does not mean that mass spectrometry cannot be used to analyze polymeric plasticizers; in fact, MS is very popular for characterizing macromolecules, such as molecular weight distributions, identifying end groups, and characterizing repeating monomer units. However, applying it to complex mixtures of natural or industrial polymers requires component separation work that is beyond the purpose of this paper.

Chloroparaffins (CPs, N=4)

Oligomeric plasticizers consisting of chlorinated, short chain paraffins ranging from about 10-30 Carbon atoms. CPs are divided by their chain lengths (short C10-13, intermediate C14-17, long C18-30) and their chlorine concentration; however, any given batch of CP plasticizer is polydisperse about the expected range and contains a large number of isomers.[5] While CPs are regulated in several regions for documented toxicity and bioaccumulation,[6-8] they are still generated in high volume and used for applications such as flame retardants, metallurgy, and some plastics applications (e.g., PVC, PU, and rubbers).[5, 6] The chloroparaffin samples yielded significant fluorescence interference under Raman measurement, with the 70 % chloride sample exhibiting the most fluorescence interference among all samples in the library.

RS: The primary Raman bands associated with chloroparaffins include the C-Cl in-plane deformation vibration (279 to 307) cm^{-1}, C-Cl deformation vibration (356 to 363) cm^{-1}, C-Cl stretching bands at (612 to 620) cm^{-1} and (671 to 699) cm^{-1}, and CH_2 deformation vibration (1426 to 1435) cm^{-1}. The chloroparaffin samples yielded significant fluorescence interference, with the 70 % chloride sample exhibiting the most fluorescence interference among all samples in the library.

Epoxidized Oils (N=3)

Three primary bio-based oils are used to produce epoxidized oil plasticizers.[5] These include soybean oil, linseed oil, and tall oil, which are derived from soybeans, flax seeds, and a byproduct of the lumber industry, respectively. These oils are composed of mixtures of fatty acids, including linoleic, linolenic, oleic, stearic, and palmitic in different ratios (**Figure 1**).

RS: The primary bands in epoxidized oil plasticizers include epoxide ring vibration (891 to 895) cm^{-1}, epoxide C-O stretching (1268 to 1270) cm^{-1}, $(CH_2)_n$ twisting (1301 to 1303) cm^{-1}, CH_2 vibration (1440 to 1445) cm^{-1}, and carbonyl stretching (1735 to 1744) cm^{-1}. A band at 1659 cm^{-1} in the epoxidized linseed oil indicates that the studied sample retained some unsaturation after the epoxidation reaction.

XI. Raman peak assignments by core functional family

Peak intensity descriptors: vw – very weak, w – weak, m – medium, s – strong, vs – very strong

Fluorescence descriptors: Assigned qualitatively where-in, *minimal* – little to no background shape due to fluorescence, *moderate* – some visible contributions to the background from fluorescence although spectra is readily interpretable, *high* – these samples had large background contributions from fluorescence and may yield poor spectra on a continuous wave Raman setup.

Spectra of the data may be plotted using different time gatings to visualize fluorescence contributions via the Google Colab link in the companion GitHub: https://colab.research.google.com/github/usnistgov/TGRS-Plasticizer-Library/blob/main/TG%20Raman%20Library%20work%20process.ipynb

Table S7. Raman peak assignments and relative strengths by core functional group

Peaks (cm^{-1})	Assignment	Strength
Adipate (N = 7)		
	fluorescence	minimal
1058 to 1064	straight chain alkane C-C stretching	w-m
1301 to 1304	$(CH_2)_n$ twisting	m
1440 to 1448	CH_2 vibration	vs
1733 to 1738	C=O stretch	w
Azelate (N=5)		
	fluorescence	moderate
1064 to 1089	straight chain alkane C-C stretching	vw-w
1303 to 1304	$(CH_2)_n$ twisting	m
1440 to 1448	CH_2 vibration	vs
1734 to 1736	C=O stretch	w (m in DMAz)
Chloroparaffins (N=4)		
	fluorescence	moderate to high
279 to 307	C-Cl in-plane deformation vibration	w
356 to 363	C-Cl deformation vibration	vw-w
612 to 620	C-Cl stretching band	s
671 to 699	C-Cl stretching band	m-s
1426 to 1435	CH_2 vibration	w-m (vw in 70 % Cl)
Citrates (N=4)		
	fluorescence	moderate
1115 to 1122	tert-alcohol C-O stretching vibration	vw-w
1450 to 1453	CH_2 vibration	m
1736 to 1741	C=O stretch	w
Dibenzoates (N=3)		
	fluorescence	minimal
616 to 618	aromatic in-plane ring deformation vibration	w
675 to 676	out-of-plane ring deformation vibration	vw
1001 to 1002	=C-H in-plane ring deformation vibration	vs
1160	ester symmetric COC stretching	vw
1273 to 1275	ester asymmetric COC stretching	vw-w

1451 to 1452	CH_2 vibration	vw
1715 to 1717	C=O stretch	w
1602	Ring stretching	m
**Epoxidized oils (N=3)**		
	fluorescence	moderate
891 to 895	epoxide ring vibration	w-m
1268 to 1270	epoxide C-O stretching (strong overlap with C-C stretching)	w-m
1301 to 1304	$(CH_2)_n$ twisting	m
1440 to 1445	CH_2 vibration	s
1735 to 1744	C=O stretch	vw
**Isophthalates (N=3)**		
	fluorescence	minimal
655 to 659	ring in-plane bending vibration	w
1003 to 1004	=C-H in-plane ring deformation vibration	vs
1154 to 1161	ester symmetric COC stretching	vw
1301 to 1308	$(CH_2)_n$ twisting	w
1438 to 1448	CH_2 vibration	vw-w
1587 to 1591	Ring stretching	w
1719 to 1731	C=O stretchghing	m (s in DMIP)
**Oleates (N = 4)**		
	fluorescence	moderate
1299 to 1303	$(CH_2)_n$ twisting	w-m
1438 to 1441	CH_2 vibration	m-s
1653 to 1657	Cis-dialkyl alkenes C=C stretching	w
1735 to 1742	C=O stretching	vw
**Phosphates (aliphatic) (N=2)**		
	fluorescence	minimal
1025 to 1029	P-O-C stretching	vw
1222 to 1233	P-O-C stretching	vw
1289 to 1301	P=O stretching	s
1445 to 1458	P-CH_2 deformation vibration	vs
**Phosphates (aryl) (N = 5)**		
	fluorescence	minimal
614 to 616	aromatic in-plane ring deformation vibration	w
1007	=C-H in-plane ring deformation vibration	vs
1025 to 1029	P-O-C stretching	vw
1156 to 1165	aromatic P-C stretching	vw
1222 to 1233	P-O-C stretching	vw
1289 to 1301	P=O stretching	vw
1445 to 1458	P-CH_2 deformation vibration	vw
1592 to 1597	Ring stretching	vw-w
**Phthalates (N = 16)**		
	fluorescence	minimal
649 to 651	ring in-plane bending vibration	w-m
1038 to 1040	ring C-H in-plane bending vibration	m-s

1161 to 1165	ester symmetric COC stretching	vw
1271 to 1299	ester asymmetric COC stretching	w-m
1438 to 1451	CH_2 vibration	w-m
1579 to 1581	Ring stretching	w
1598 to 1600	Ring stretching	m
1721 to 1728	C=O stretching	m
Ricinoleates (N = 6)		
	fluorescence	moderate to high
1264 to 1266	secondary-alcohol in-plane O-H deformation vibration	w-m
1301 to 1303	$(CH_2)_n$ twisting	m
1438 to 1441	CH_2 vibration	s-vs
1655 to 1657	Cis-dialkyl alkenes C=C stretching	m-s
1735 to 1742	C=O stretching	vw-w
Sebacates (N = 3)		
	fluorescence	minimal
1064 to 1066	straight chain alkane C-C stretching	w-m
1301 to 1304	$(CH_2)_n$ twisting	m-s (vw in DMS)
1442 to 1447	CH_2 vibration	vs (w in DMS)
1735 to 1739	C=O stretching	vw-w
Stearates (N = 2)		
	fluorescence	moderate
1062 to 1066	straight chain alkane C-C stretching	m-s
1124 to 1129	straight chain alkanes C-C doublet	w-s
1294 to 1301	$(CH_2)_n$ twisting	s-vs
1438 to 1442	CH_2 vibration	vs
1739 to 1742	C=O stretching	vw
Monostearates (N=3)		
	fluorescence	minimal
1129	straight chain C-C skeletal vibration	s-vs
1296	$(CH_2)_n$ twisting	vs
1100 to 1106	straight chain alkane C-C stretching	vw-w
1438 to 1440	CH_2 vibration	m-s
1460 to 1462	CH_2 vibration	m
1722 to 1739	C=O stretching	vw
Sulfonamides (N = 2)		
	fluorescence	minimal
287 to 292	SO_2 rocking	m-s
542 to 544	SO_2 wagging	w-m
687 to 693	N-H out-of-plane bending vibration	w-m
805 to 809	C-C stretching	w-m
1046 to 1049	ring C-H in-plane bending vibration	w-s
1147 to 1155	symmetric SO_2 stretching	s-vs
1595 to 1598	Ring str, NH scissor vibration	w
Trimellitates (N=4)		
	fluorescence	minimal
665 to 667	ring in-plane bending vibration	m

1066 to 1069	straight chain alkane C-C stretching	w
1116 to 1135	aromatic =C-H in-plane deformation vibrations	w
1303 to 1306	$(CH_2)_n$ twisting	m
1438 to 1450	CH_2 vibration	m
1574 to 1577	Ring stretching	w
1610 to 1612	Ring stretching	vs
1725 to 1730	C=O stretching	s

XII. Time-gated Raman analysis of SRM 2860

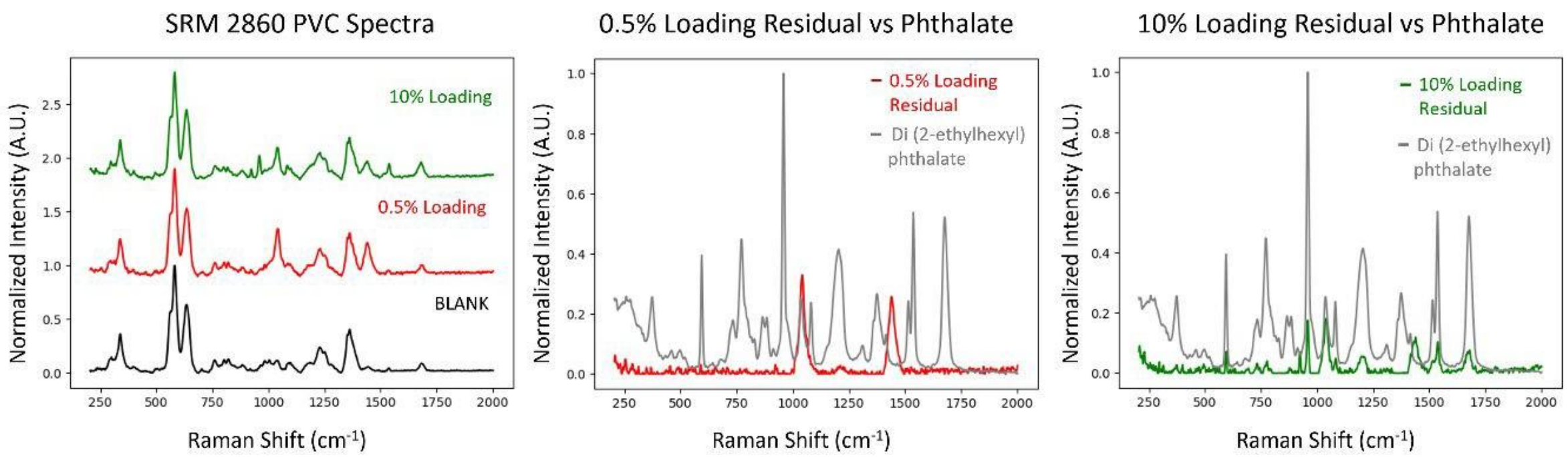

Figure S4. Plotted left to right: 1) pre-processed TGRS spectra of samples contained in NIST SRM 2860 (Phthalates in Polyvinyl Chloride). 2) The residual of the 0.5 % Loading plotted against DEHP. 3) The residual of the 10 % Loading plotted against DEHP.

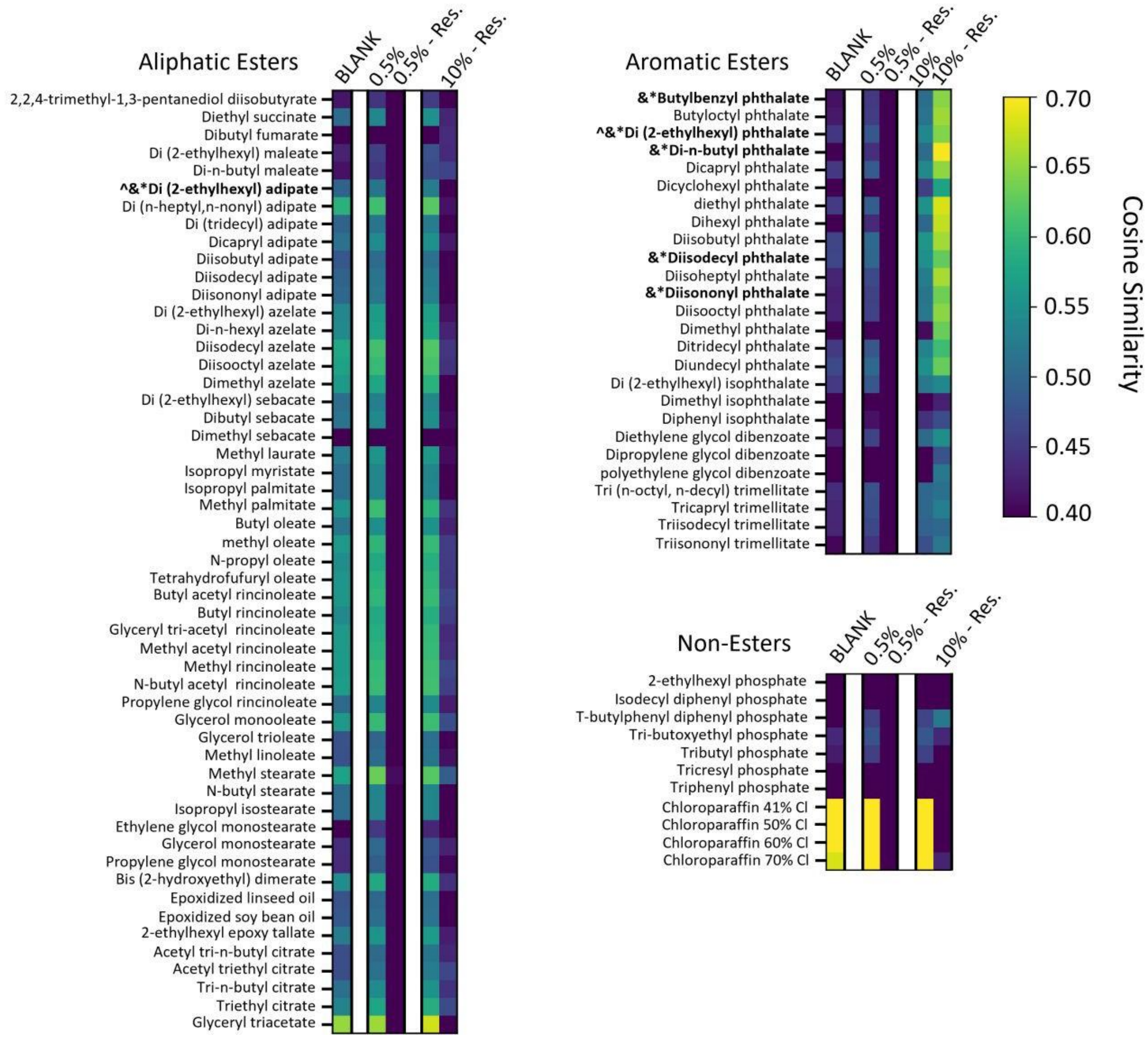

Figure S5. TGRS cosine similarity heatmaps comparing the SRM 2860 BLANK, 0.5% Loading, 0.5% Residual, 10% Loading, and 10% Residual against every plasticizer in the TGRS library along the wave number region of (200 – 2000) cm^{-1}. Scale bar set to maximize contrast. Key: symbols indicate presence in the (^) Blank, (&) 0.5%, and (*) 10%.

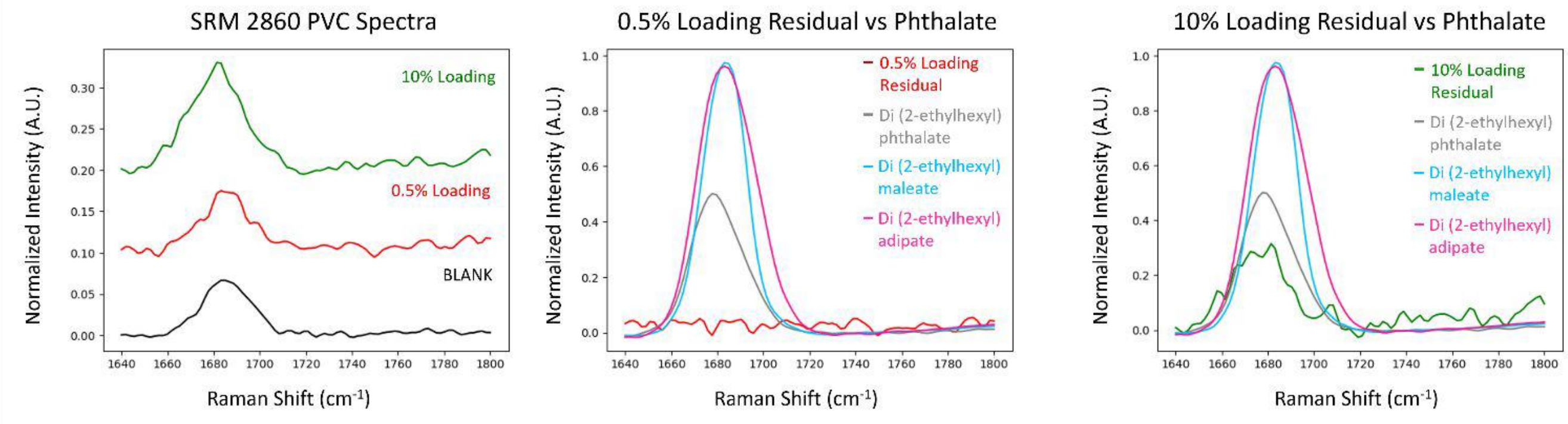

Figure S6. Plotted left to right: 1) pre-processed TGRS spectra of samples contained in NIST SRM 2860 (Phthalates in Polyvinyl Chloride) focused about the carbonyl band (1640 to 1800) cm^{-1}. 2) The residual of the 0.5 % Loading plotted against a selection of plasticizers. 3) The residual of the 10 % Loading plotted against a selection of plasticizers. Note that the absence of a carbonyl band in the 0.5 % residual is indicative of trace presence of DEHP and DEHA in the BLANK.

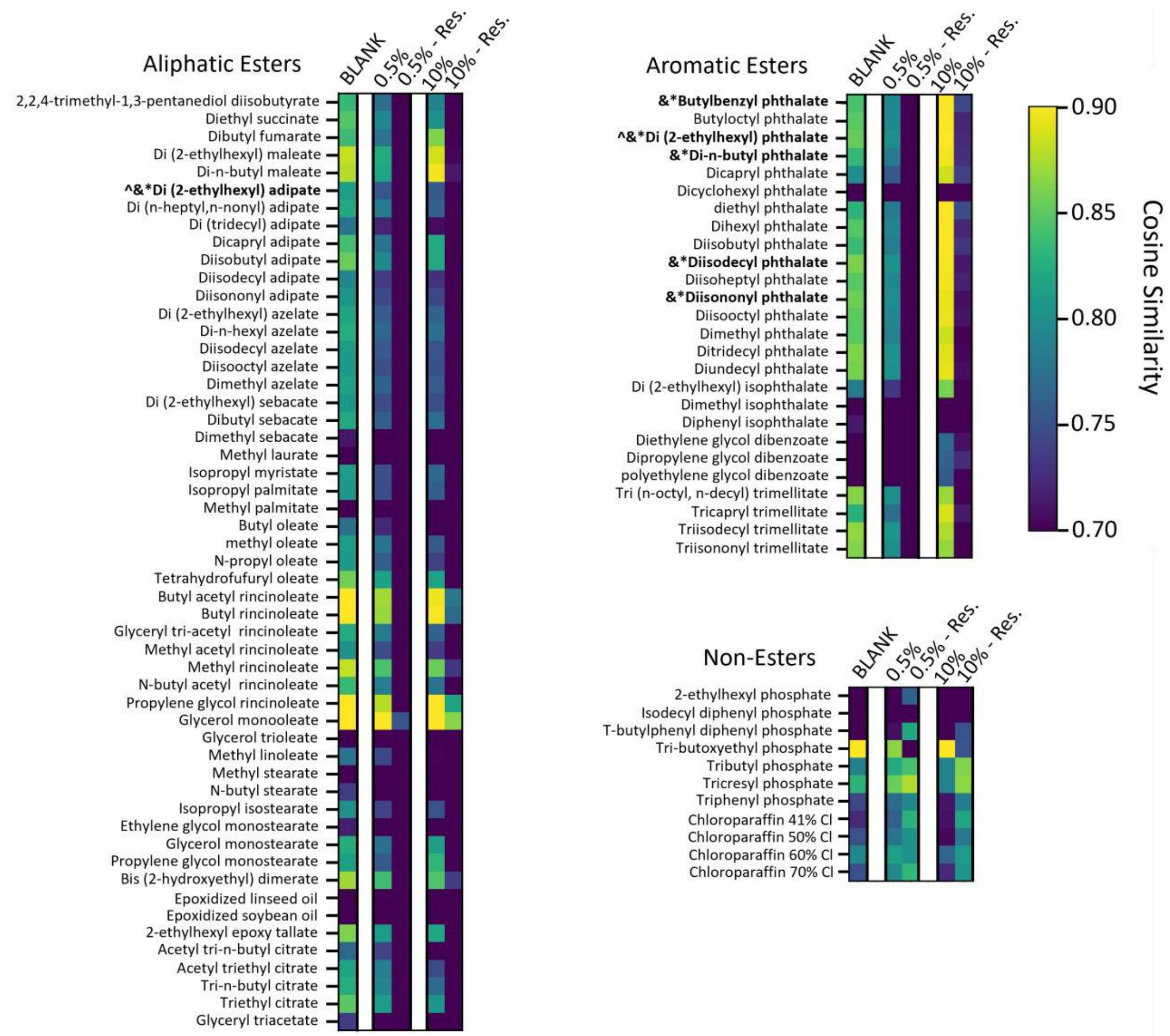

Figure S7. TGRS cosine similarity heatmaps comparing the SRM 2860 BLANK, 0.5% Loading, 0.5% Residual, 10 % Loading, and 10 % Residual against every plasticizer in the TGRS library along the wave number region of (1640 to 1800) cm^{-1}. Scale bar set to maximize contrast. Key: symbols indicate presence in the (^) Blank, (&) 0.5 %, and (*) 10 %.

XIII. APCI-LC-MS/MS analysis of SRM 2860

The purpose of this experiment was to demonstrate that APCI-LC-MS/MS is appropriate for quantifying plasticizers. However, the chromatographic separation has not been optimized. We believe that in the future it should be performed with different instrumentation, specifically a Triple Quad, and using MRM transitions instead of a timsTOF for better accuracy and precision.

The additive extraction from the SRM 2860 sample (PVC containing 0.5 % of phthalates) was performed as explained in the Methods section of the manuscript and analyzed by LC-MS/MS using a Thermo Fisher Scientific Vanquish UHPLC liquid chromatography instrument coupled to a Bruker timsTOF Pro mass spectrometer.

The conditions for the reversed-phase chromatography are described in **Figure S7**. The gradient runs for 15 minutes using the timetable shown in **Figure S7**. and mobile phase A: Water (0.1 % formic acid) and mobile phase B: Acetonitrile (0.1 % formic acid). The chromatography column was an ACQUITY UPLC CSH C18 Column, 130Å, 1.7 µm, 2.1 mm X 100 mm, and the flow rate was 0.3 mL/min.

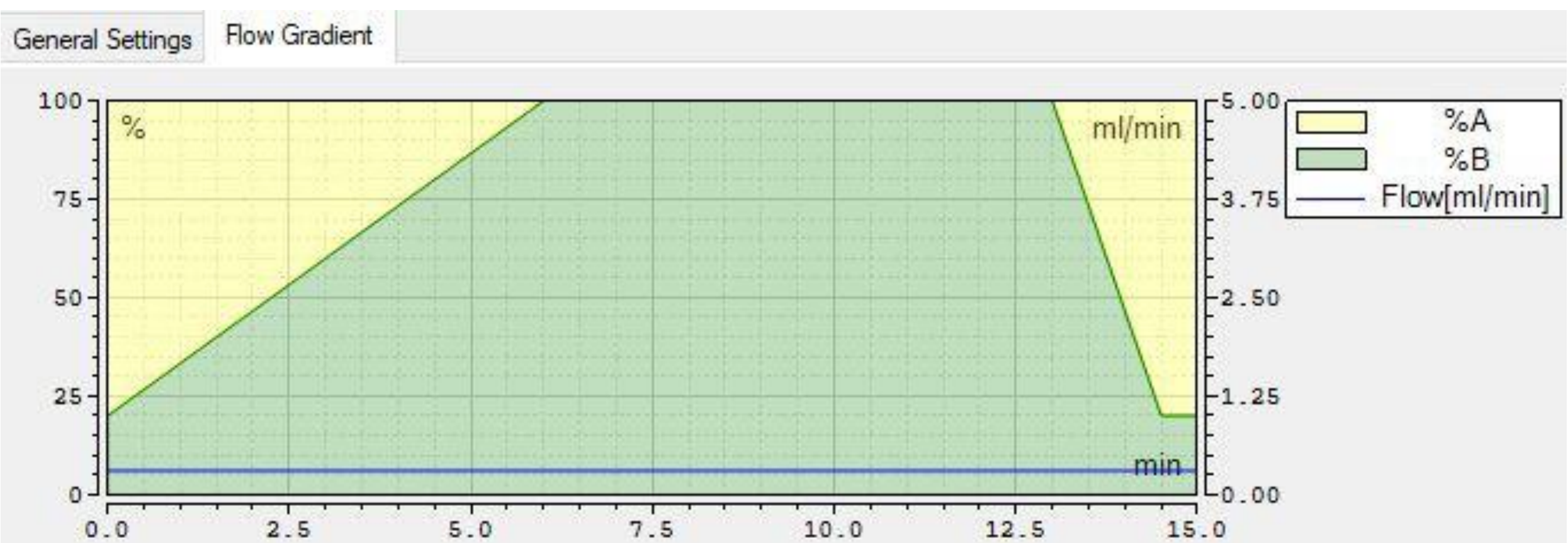

No	Time	Flow [ml/min]	%B	Curve
2	0.000	0.300	20.0	5
3	*New Row*			
4	0.000	Run		
5	0.000	0.300	20.0	5
6	6.000	0.300	100.0	5
7	13.000	0.300	100.0	5
8	14.500	0.300	20.0	5
9	15.000	0.300	20.0	5
10	*New Row*			
11	15.000	Stop Run		

Insert

Delete

Figure S7. Timetable for the chromatography of the SRM 2860 (PVC 0.5 % content of phthalates). Mobile phase A is Water (0.1 % formic acid), and mobile phase B is Acetonitrile (0.1 % formic acid).

The MS setting for the timsTOF is given below:

Chromatogram

Chromatogram Traces

Enabled	Color	Type	Masses	Width	Polarity	Filter	Mobility
On	Red	BPC			±	ALL	
Off	unknown	EIC	322.06	±0.5	±	ALL	
On	unknown	EIC	427.38	±0.5	±	ALL	

Mobilogram

Mobilogram Traces

Enabled	Color	Type	Masses	Width	Polarity	Filter
Off	Red	TIM			±	MS
On	Black	EIM	118.08	±0.5	+	MS
On	Dark Red	EIM	322.05	±0.5	+	MS
On	Blue	EIM	622.03	±0.5	+	MS
On	Magenta	EIM	922.01	±0.5	+	MS
On	unknown	EIM	118.09	±0.5	±	ALL

Calibration - m/z

**Ion Polarity:**	Positive	**Reference List:**	Tuning Mix APCI-TOF_TuneMix_Test
**Ion Polarity:**	Negative	**Reference List:**	Tuning Mix APCI-TOF

Calibration - Mobility

**Ion Polarity:**	Positive	**Reference List:**	Tuning Mix APCI-TOF_TuneMix_Test
**Ion Polarity:**	Negative	**Reference List:**	Tuning Mix ES-TOF (ESI)

Calibration - Mobility - In-Batch

**In-Batch Calibration:**	Off	**Reference List:**	Tuning Mix ES-TOF CCS (In-Batch)

Time Table Overview

Segment List

Segment	End Time [min]	Scan Mode
1	31.0	MS

Main

**Polarity:**	Positive	**TIMS enable:**	On
		**Scan Mode:**	MS
**Mass Range from:**	20 m/z	**Mass Range to:**	1200 m/z
**1/K0 Start:**	0.10 Vs/cm²	**1/K0 End:**	1.48 Vs/cm²
**Rolling Average:**	Off	**Rolling Average No.:**	3
**View:**	Expert		

Mode - General

**Enable TIMS:**	On	**Mark as Calibration Segment:**	Off
**Save Spectra:**	Save Frame	**Mass Spectra Peak Detection:**	Use Max. Intensity
**Absolute Threshold:**	100	**Intensity Threshold:**	Absolute
**Intensity Threshold:**	100000.00		

Mode - TIMS

**ICC Mode:**	ICC	**Target:**	7.5 Mio.
**imeX mode:**	Custom	**Resolution:**	Custom
**1/K0 Start:**	0.10 Vs/cm²	**1/K0 End:**	1.48 Vs/cm²
**Ramp Time:**	100.0 ms	**Spectra Rate:**	n/a Hz
**Advanced Parameters:**	On		
**Lock accumul. to mobility range:**	On	**Lock Duty Cycle to 100 %:**	Off
**Accumulation Time:**	100.0 ms	**Duty Cycle:**	n/a %
**Cycle Time:**	n/a ms		

Source

**Source:**	VIP-HESI-APCI		
**End Plate Offset:**	500 V	**Capillary:**	3500 V
**Corona:**	3500 nA		
**Nebulizer:**	3.0 Bar		
**Dry Gas:**	8.0 l/min	**Dry Temp:**	250 °C
**Probe Gas Temp:**	250 °C		
**Probe Gas:**	3.0 l/min	**Exhaust:**	On

Tune - General

**Deflection 1 Delta:**	60.0 V		
**Funnel 1 RF:**	250.0 Vpp	**Funnel 2 RF:**	200.0 Vpp
**isCID Energy:**	0.0 eV	**Multipole RF:**	200.0 Vpp
**Ion Energy:**	5.0 eV	**Low Mass:**	60.00 m/z
**Collision Energy:**	10.0 eV	**Collision RF:**	450.0 Vpp
**Transfer Time:**	65.0 µs	**Pre Pulse Storage:**	3.0 µs
**High Sensitivity Detection:**	Off	**Stepping:**	Off

Tune - TIMS

**Dt1 (Defl. Transfer -> Capillary Exit):**	-20.0 V	**Dt2 (Defl. Discard -> Defl. Transfer):**	-120.0 V
**Dt3 (Funnel 1 In -> Defl. Transfer):**	50.0 V	**Dt4 (Accu. Trap -> Funnel 1 In):**	50.0 V
**Dt5 (Accu. Exit -> Accu. Transfer):**	0.0 V	**Dt6 (Ramp Start -> Accu. Exit):**	20.0 V
**Funnel 1 RF:**	300.0 Vpp	**Collision Cell In:**	140.0 V

MS/MS

**TIMS Stepping:**	On	**Profile:**	3c903ccc-<br>fa78-40be-8c49-395

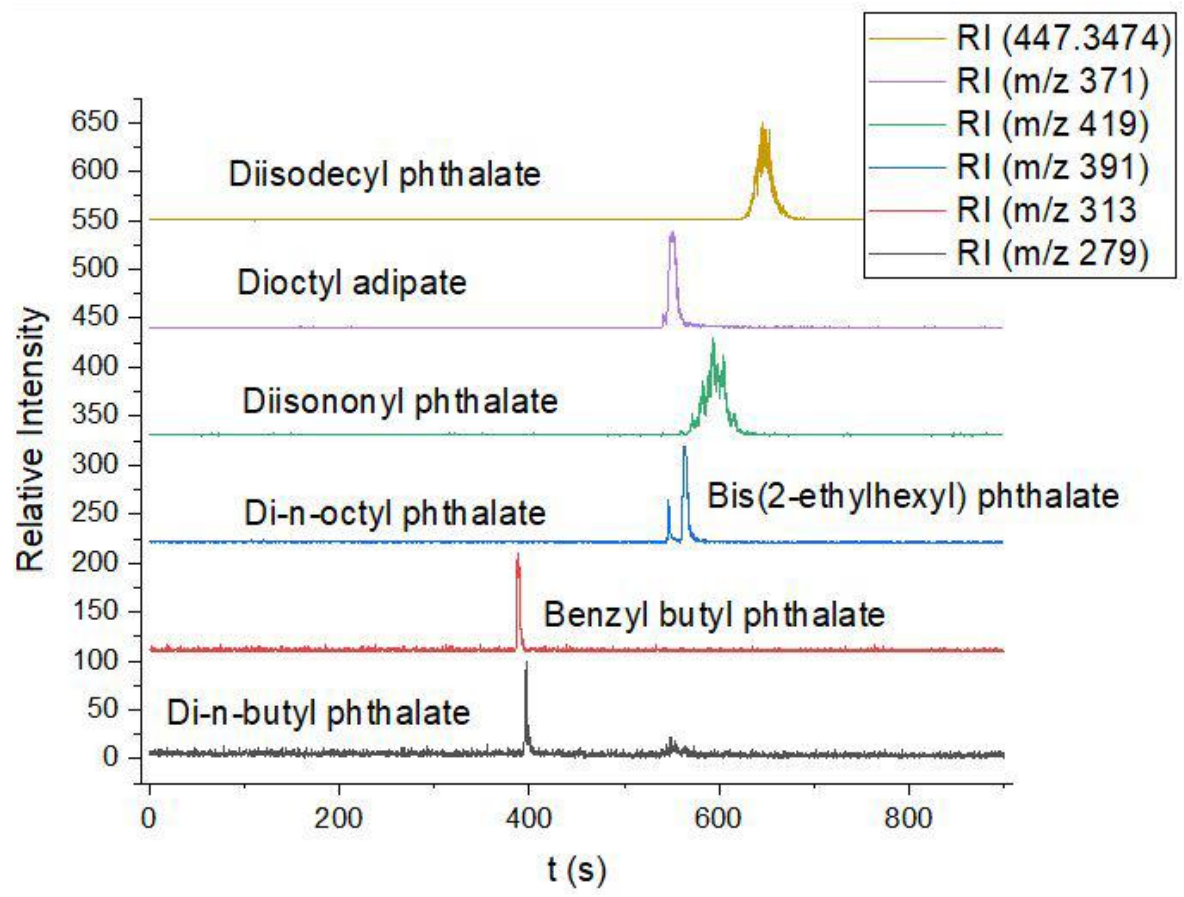

Figure S8. APCI-LC-MS/MS separation of the SRM 2860 sample (PVC containing 0.5 % of phthalates).

The chromatographic separation of all additives is good, particularly for two compounds with similar m/z values, di-n-octyl phthalate (m/z 391.2843) and Bis(2-ethylhexyl) phthalate (m/z 391.2848). Both compounds are included in the same trace (the blue one in **Figure S8**). Also, the plasticizer used as the solvent for the other plasticizers, dioctyl adipate, elutes at retention times between the previously mentioned compounds: 9.1 min, 9.2 min, and 9.4 min, respectively. In addition, diisononyl phthalate and diisodecyl phthalate show relatively broad peaks, but still, the separation is good. None of these issues affect the quantitation of Bis(2-ethylhexyl) phthalate, one of the two certified additive-concentration values. Unfortunately, we were unable to acquire a sample of di-n-octyl phthalate to assess the NIST-certified concentration value.

The calibration was done with a pure external standard of Bis(2-ethylhexyl) phthalate. The calibration curve is shown below (**Figure S9**). An appropriate analyte loading for the column we are using in this experiment is in the range from a few nanograms to less than 500 nanograms. Concentration values of the standard are expressed in ng/µL to keep track of maintaining an appropriate load of the chromatographic column. Although the ionization efficiency is high, injections below 2 ng/µL or above 900 ng/µL fall outside the linear response range.

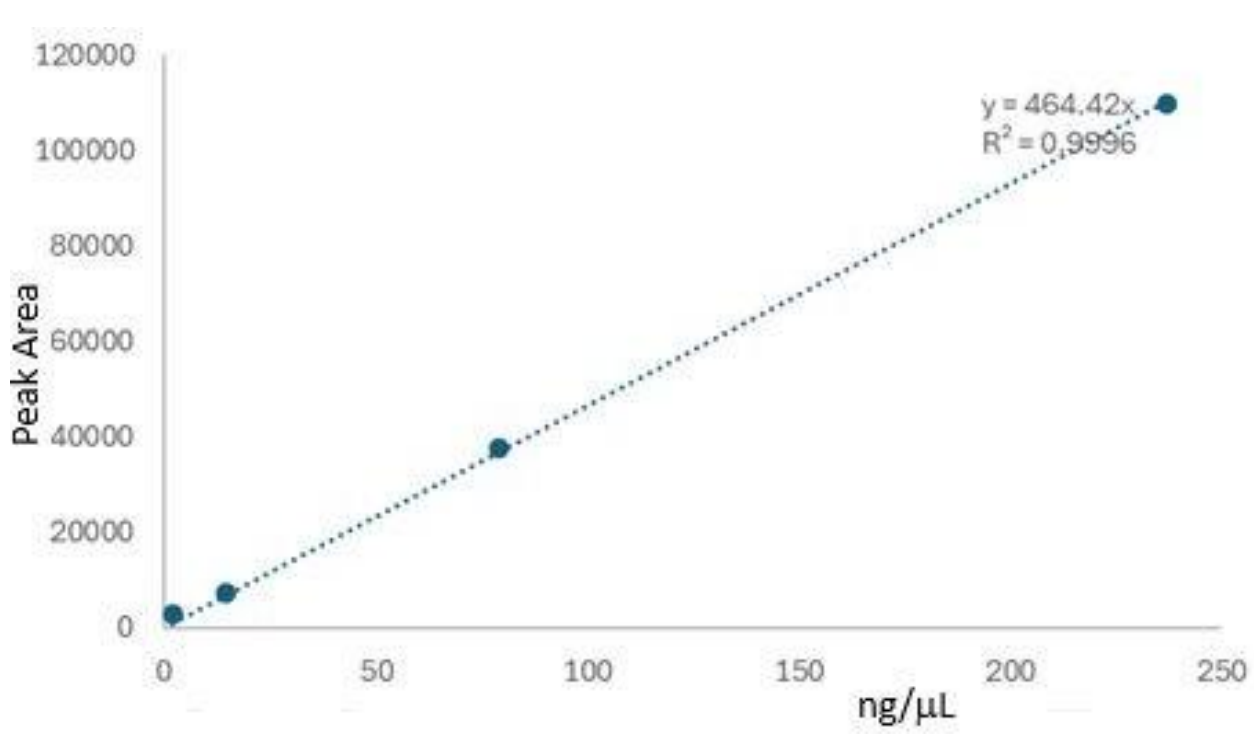

Figure S9. SRM 2860 (0.5 % Phthalates in PVC). Calibration curve of an external standard of Bis(2-ethylhexyl) phthalate.

XIV. References

(1) McCreery, R. L. Calibration and validation. In *Raman Spectroscopy for Chemical Analysis*, Winefordner, J. D. Ed.; Vol. 157; 2000; pp 251–291.
(2) McCreery, R. L. Signal To Noise in Raman Spectroscopy. In *Raman Spectroscopy for Chemical Analysis*, Winefordner, J. D. Ed.; Vol. 157; 2000; pp 49–71.
(3) Eilers, P. H. C.; Boelens, H. F. M. Baseline Correction with Asymmetric Least Squares Smoothing. *Leiden University Medical Centre report* **2005**, *1* (1).
(4) Socrates, G. *Infrared and Raman Characteristic Group Frequencies*; John Wiley & Sons, LTD, 2001.
(5) Wypych, G. Plasticizer types. In *Handbook of Plasticizers*, 4 ed.; G., W. Ed.; ChemTec Publishing, 2023; pp 9–104.
(6) Chen, W.; Liu, J. A review on biological occurrence, bioaccumulation, transmission and metabolism of chlorinated paraffins. *Critical Reviews in Environmental Science and Technology* **2023**, *54* (5). DOI: https://doi.org/10.1080/10643389.2023.2246615.
(7) EPA. *Risk management for short-chain chlorinated paraffins*. United States Environmental Protection Agency, https://www.epa.gov/assessing-and-managing-chemicals-under-tsca/risk-management-short-chain-chlorinated-paraffins (accessed 2025 December 15).
(8) Guida, Y.; Capella, R.; Weber, R. Chlorinated paraffins in the technosphere: A review of available information and data gaps demonstrating the need to support the Stockholm Convention implementation. *Emerging Contaminants* **2020**, *6*, 143–154. DOI: https://doi.org/10.1016/j.emcon.2020.03.003.
